# RANK is a poor prognosis marker and a therapeutic target in ER‐negative postmenopausal breast cancer

**DOI:** 10.15252/emmm.202216715

**Published:** 2023-03-07

**Authors:** Marina Ciscar, Eva M Trinidad, Gema Perez‐Chacon, Mansour Alsaleem, Maria Jimenez, Maria J Jimenez‐Santos, Hector Perez‐Montoyo, Adrian Sanz‐Moreno, Andrea Vethencourt, Michael Toss, Anna Petit, Maria T Soler‐Monso, Victor Lopez, Jorge Gomez‐Miragaya, Clara Gomez‐Aleza, Lacey E Dobrolecki, Michael T Lewis, Alejandra Bruna, Silvana Mouron, Miguel Quintela‐Fandino, Fatima Al‐Shahrour, Antonio Martinez‐Aranda, Angels Sierra, Andrew R Green, Emad Rakha, Eva Gonzalez‐Suarez

**Affiliations:** ^1^ Molecular Oncology, Spanish National Cancer Research Centre (CNIO) Madrid Spain; ^2^ Oncobell, Bellvitge Biomedical Research Institute (IDIBELL) Barcelona Spain; ^3^ Nottingham Breast Cancer Research Centre, Academic Unit for Translational Medical Sciences, School of Medicine University of Nottingham Biodiscovery Institute, University Park Nottingham UK; ^4^ Bioinformatics Unit, Structural Biology, Spanish National Cancer Research Centre (CNIO) Madrid Spain; ^5^ Medical Oncology, Breast Unit, Catalan Institute of Oncology (ICO) University Hospital of Bellvitge Barcelona Spain; ^6^ Pathology Department University Hospital of Bellvitge, IDIBELL Barcelona Spain; ^7^ Molecular and Cellular Biology and Radiology The Lester and Sue Smith Breast Center, Baylor College of Medicine Houston Texas USA; ^8^ Cancer Research UK Cambridge Centre Cambridge UK; ^9^ Breast Cancer Clinical Research Unit, Clinical Research Program Spanish National Cancer Research Centre (CNIO) Madrid Spain; ^10^ Present address: Department of Applied Medical Science, Applied College Qassim University Unayzah Saudi Arabia; ^11^ Present address: Molecular Pathology Division Centre for Paediatric Oncology Experimental Medicine Centre for Cancer Evolution The Institute of Cancer Research London UK; ^12^ Present address: Laboratory of Experimental Oncological Neurosurgery, Neurosurgery Service Hospital Clinic de Barcelona‐FCRB Barcelona Spain

**Keywords:** breast cancer patient‐derived xenografts, ER negative breast cancer, menopause, pharmacological RANKL inhibitors, RANK‐RANKL, Biomarkers, Cancer

## Abstract

Despite strong preclinical data, the therapeutic benefit of the RANKL inhibitor, denosumab, in breast cancer patients, beyond the bone, is unclear. Aiming to select patients who may benefit from denosumab, we hereby analyzed RANK and RANKL protein expression in more than 2,000 breast tumors (777 estrogen receptor‐negative, ER^−^) from four independent cohorts. RANK protein expression was more frequent in ER^−^ tumors, where it associated with poor outcome and poor response to chemotherapy. In ER^−^ breast cancer patient‐derived orthoxenografts (PDXs), RANKL inhibition reduced tumor cell proliferation and stemness, regulated tumor immunity and metabolism, and improved response to chemotherapy. Intriguingly, tumor RANK protein expression associated with poor prognosis in postmenopausal breast cancer patients, activation of NFKB signaling, and modulation of immune and metabolic pathways, suggesting that RANK signaling increases after menopause. Our results demonstrate that RANK protein expression is an independent biomarker of poor prognosis in postmenopausal and ER^−^ breast cancer patients and support the therapeutic benefit of RANK pathway inhibitors, such as denosumab, in breast cancer patients with RANK^+^ ER^−^ tumors after menopause.

## Graphical abstract

The analyses of RANK and RANKL expression in large cohorts of breast cancer samples and functional studies in RANK+ breast cancer patient‐derived xenografts (PDXs) revealed a key role for RANK in postmenopausal women with estrogen receptor negative ER^‐^ breast cancer.

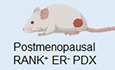

## Introduction

Despite recent advances in treatment, breast cancer (BC) is the main cause of mortality by cancer in women, highlighting the unmet need of identifying new prognosis markers and personalized treatments. BC shows a high pathological and biological heterogeneity in histology, genetics, and sensitivity to therapies. The expression of estrogen and progesterone receptor (ER, PR), human epidermal growth factor receptor 2 (HER2) and KI67 are determinant for BC prognosis and treatment (Perou *et al*, 2000; Cheang *et al*, 2009). Tumors lacking ER, PR, and HER2 (triple negative BC, TNBC) have the worst outcome among BC subtypes in part due to limited therapeutic options (Dent *et al*, 2007).

RANKL and its receptor RANK are potential predictor biomarkers in BC. RANK is expressed on tumor cells in 40% of hormone receptor‐negative tumors and 20% of the luminal tumors (Palafox *et al*, 2012). RANK expression is associated with a higher risk of relapse and death (Pfitzner *et al*, 2014). By contrast, RANKL is rarely found in tumor cells, being mostly restricted to the luminal A‐like subset (Pfitzner *et al*, 2014; Azim *et al*, 2015).

Preclinical studies support RANK signaling as a therapeutic target in BC; it regulates mammary tumor initiation mediating the proliferative response to progesterone and the expansion of mammary stem cells and progenitors (Gonzalez‐Suarez *et al*, 2010; Joshi *et al*, 2010; Schramek *et al*, 2010). Loss of RANK signaling prevents or attenuates mammary tumorigenesis, induces tumor cell apoptosis and/or differentiation, reduces recurrence and metastasis in Rank^+^ mouse mammary tumors, and enhances tumor immunity (Gonzalez‐Suarez *et al*, 2010; Schramek *et al*, 2010; Nolan *et al*, 2016; Yoldi *et al*, 2016; Gómez‐Aleza *et al*, 2020).

Denosumab, a fully human monoclonal antibody against RANKL, is currently used for the treatment of osteoporosis and skeletal‐related events arising from bone metastases (Miyazaki *et al*, 2014). In BC, adjuvant denosumab improved the disease‐free survival (DFS) in postmenopausal women with luminal BC (ABCSG‐18 trial/NCT00556374; Gnant *et al*, 2018), but in the D‐CARE study/NCT01077154, no benefit in survival upon denosumab was found in any subgroup (Coleman *et al*, 2020). These conflicting results highlight the need of further knowledge in the understanding of RANK biology in BC and its therapeutic potential.

In this study, we have evaluated the potential value of RANK and RANKL protein expression as clinical predictors of BC prognosis and the therapeutic value of targeting RANK signaling in human BC.

## Results

### RANK expression in tumor cells associates with ER/PR‐negative tumors

**Figure 1:**
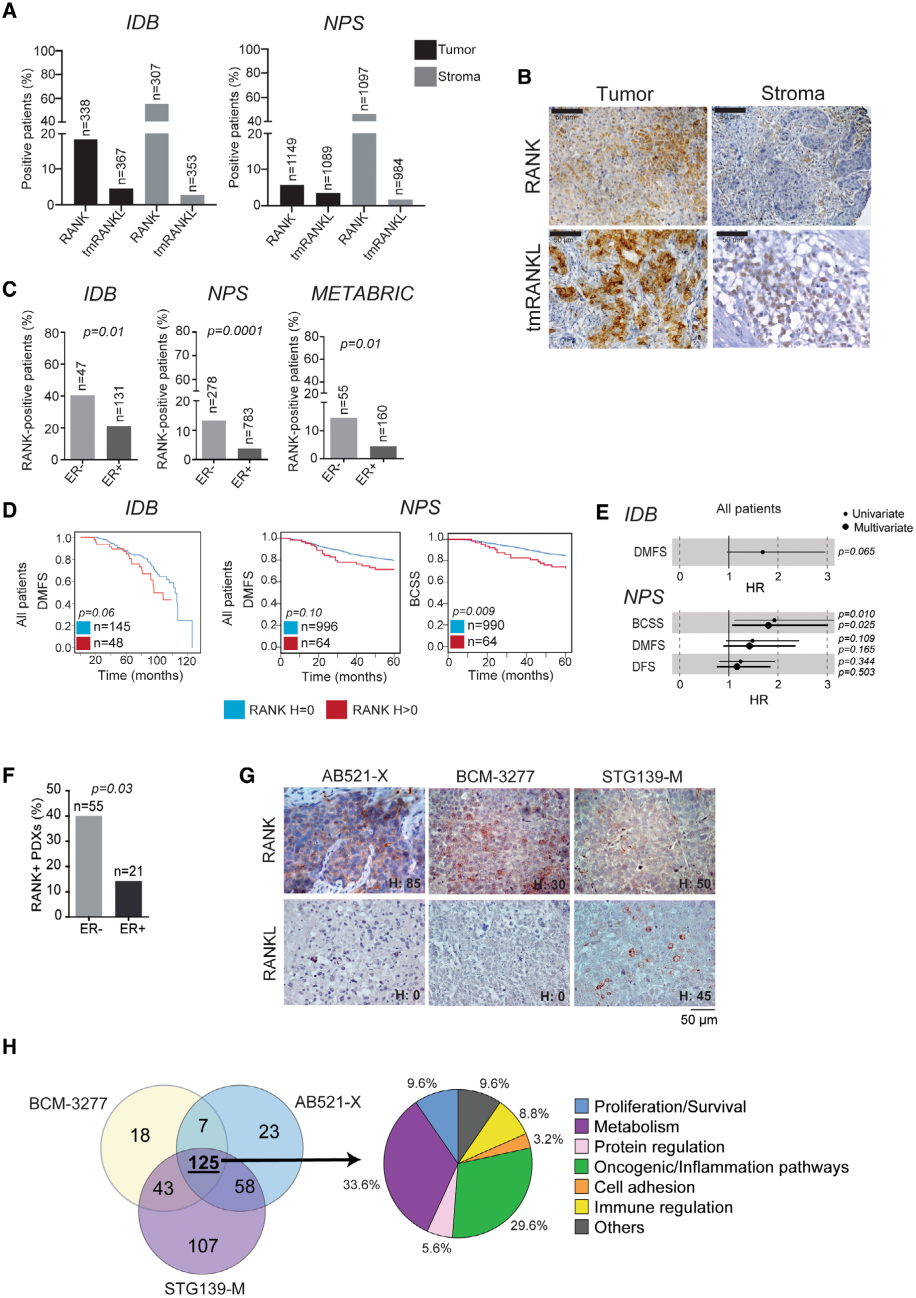
Tumor RANK is expressed and active in human BC and it associates with ER^−^ BC subtype and poor survival Percentage of patients expressing tumor and stromal RANK or tmRANKL (H > 0) in BC samples.Representative images showing RANK and tmRANKL protein expression in tumor and stromal cells in human BC determined by IHC.Percentage of BC patients with RANK^+^ tumors according to ER expression, *p*‐values (Pearson's ChiSquare test (Exact Sig. 2‐Side)).DMFS and BCSS according to RANK expression. *p*‐values (Log‐rank test (Mantel‐Cox)).Forest plots showing HR, 95% CI and *p*‐values from uni or multivariate regression analyses for the indicated survival parameters.Percentage of PDXs expressing RANK protein according to ER expression. *p*‐value (Two tailed *t*‐test).Representative images of RANK and RANKL protein expression in BC PDXs. H‐Score (H) of each PDX model is indicated.Venn diagram (left) shows the pathways (FDR < 0.25) modulated by RANKL in each PDX and those shared. Pie chart (right) represents the percentage of pathways involved in cited biological processes. Data information: (A, C, D, F) The total number of patients or PDX is shown.

**Figure EV1:**
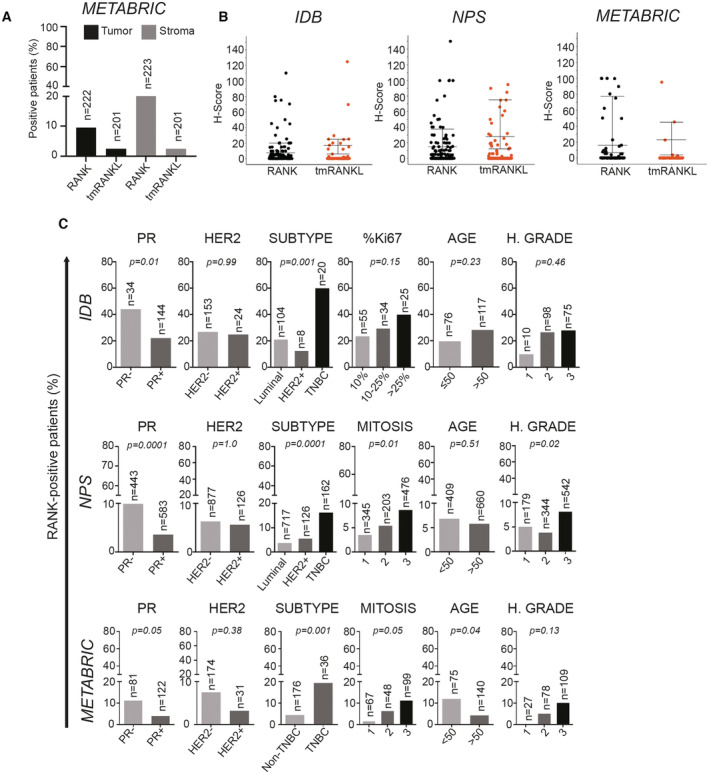
RANK is expressed in tumor and stromal cells of human BC and associates with ER/PR‐negative tumors Percentage of patients expressing RANK or tmRANKL in tumor and stromal cells in BC samples. The total number of patients is indicated.H‐Score values of tumor RANK and tmRANKL from *IDB*, *NPS* collections and the *METABRIC* dataset. Mean ± SEM is shown.Percentage of BC patients with RANK^+^ tumors according to the indicated clinicopathologic parameters in the *METABRIC* cohort. Total number of analyzed patients per parameter and *p*‐values (Pearson's Chi‐Square test (Exact Sig. 2‐ Side)) are indicated.

We analyzed the expression of RANK and RANKL proteins in two independent tissue‐microarray (TMA) collections containing all BC subtypes: *IDIBELL* (*IDB*) (*n* = 404; Martínez‐Aranda *et al*, 2015) and *Nottingham Primary Series* (*NPS*) (*n* = 1,895 samples, 298 included in the Molecular Taxonomy of Breast Cancer International Consortium (*METABRIC*); Curtis *et al*, 2012; Green *et al*, 2013). RANK protein was detected in the tumor compartment in 18.3 and 5.7% of samples from *IDB* and *NPS*, respectively, as well as in the stroma of half of the samples. Tumor expression of transmembrane RANKL (tmRANKL) was found in only 4.6% (*IDB*) and 3.5% (*NPS*) of adenocarcinomas and rarely observed in the stroma (< 3%; Fig 1A and B). Similar expression patterns for RANK and RANKL were found in the *METABRIC* subset (Fig EV1A). Fig EV1B shows the H‐Score (H) for tumor RANK and tmRANKL in the three collections.

RANK was more frequently found in the ER^−^ compared with the ER^+^ subsets of *IDB* (40.4% vs. 21.3%) and *NPS* (13% vs. 3.7%) samples (Fig 1C). In line with previous results (Gonzalez‐Suarez *et al*, 2010; Pfitzner *et al*, 2014), RANK expression (H > 0) was associated with ER/PR negativity and TNBC subtype, but not with HER2, age, tumor size or grade in all cohorts (Figs 1C and EV1C). In the *NPS*, RANK expression was also associated with a higher mitosis rate and grade (Fig EV1C; Dataset EV1). The low frequency of tmRANKL^+^ samples prevented associations with clinicopathologic parameters.

Patients with RANK^+^ tumors tended to have a poorer distant metastasis‐free survival (DMFS) (*IDB* and *NPS*) and BC‐specific survival (BCSS) (*NPS*) compared with those with RANK^−^ tumors (Fig 1D; Dataset EV1). Moreover, RANK expression (*NPS*) was associated with shorter BCSS, independent of ER, tumor grade, stage and size (Fig 1E; Dataset EV1). Altogether, our results show that RANK protein expression associates with ER^−^/PR^−^ tumors and poor outcome.

### RANK is expressed in ER ^−^ BC patient‐derived orthoxenografts (PDXs) and it is responsive to RANKL

**Figure EV2:**
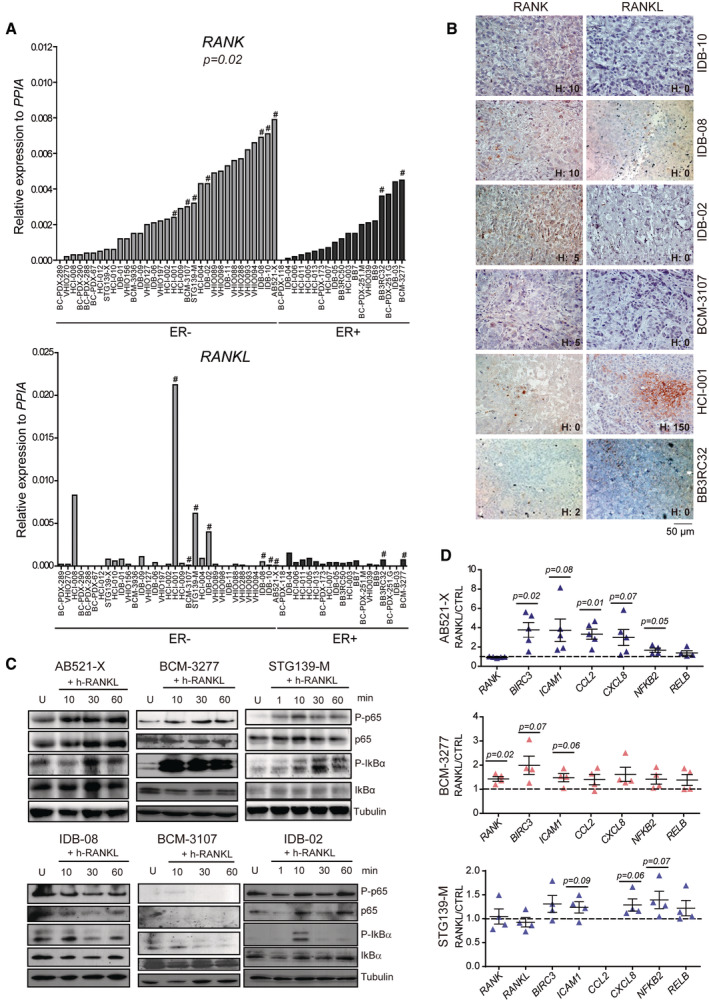
RANK is expressed and active in a subset of BC PDXs RANK and RANKL mRNA expression levels relative to *PPIA* in the indicated BC PDXs, organized according to ER status in the human tumor of origin and *RANK* mRNA expression. Two‐tailed *t*‐student test was used to evaluate the RANK/RANKL differential expression between ER^−^ and ER^+^ BC PDXs. # Indicates models where RANK and RANKL expression were analyzed by IHC.Representative images of RANK and RANKL protein expression in BC PDXs detected by IHC. H‐Score (H) of the models (and not of the picture) are shown. A total of 3–5 independent tumors per PDX were scored for RANK.Western blot analyses of P‐p65, P‐IKBα and corresponding total proteins after RANKL stimulation in the indicated PDXs. Tubulin was used as a loading control.Gene expression analyses of the indicated NFKB target genes in PDX tumor organoids after 24 h of RANKL stimulation. Expression levels relative to the untreated controls are shown. Each dot represents organoids from an independent BC PDX tumor. Mean ± SEM and *p*‐value of two‐tailed *t*‐student test are shown.

Despite encouraging results in BC mouse models and cell lines, RANK functional relevance in clinical BC remains poorly studied. Thus, we analyzed *RANK* and *RANKL* gene and protein expression in 76 PDXs from several BC collections (Derose *et al*, 2011; Zhang & Lewis, 2013; Bruna *et al*, 2016; Eyre *et al*, 2016; Gómez‐Miragaya *et al*, 2017; Gris‐Oliver *et al*, 2020). *RANK* mRNA levels were higher in PDXs derived from ER^−^ tumors than ER^+^, while *RANKL* was low or undetectable in most PDXs, with some exceptions (Fig EV2A). RANK protein was found in 40 and 14.3% of those PDX derived from ER^−^ and ER^+^ BC, respectively, whereas tmRANKL was only detected in few models (Figs 1F and G, and EV2B; Dataset EV2), recapitulating clinical patterns (Fig 1A and C). Enhanced phosphorylation of IkBα and/or p65 and upregulation of RANK/NFKB targets after RANKL treatment confirmed activation of RANK signaling in AB521‐X, BCM‐3277 and STG139‐M, but not in other RANK^+^ PDXs (Fig EV2C and D). The AB521‐X and STG139‐M models, derived from ER^−^ BCs, and the BCM‐3277 model, derived from ER^+^ BC, but ER^−^ in the PDX, were selected for *in vivo* experiments. Upon RANKL exposure *in vivo*, more than 200 pathways were regulated (FDR < 0.25) in each PDX and most (> 100) were shared between the three PDXs (Fig 1H; Dataset EV3, Table B, F, J, O). The top‐ranked RANKL‐driven common pathways (NES > 0) were related to TNF/NFKB signaling (confirming RANK activation), metabolism, oncogenic/inflammation, immunity, and proliferation (Fig 1H; Dataset EV3, Table O). These results demonstrate that RANK/RANKL expression patterns in BC PDXs recapitulate clinical findings and that activation of RANK signaling has pleiotropic effects in human breast adenocarcinomas.

### ER expression determines RANK biology and prognosis value in BC

**Figure EV3:**
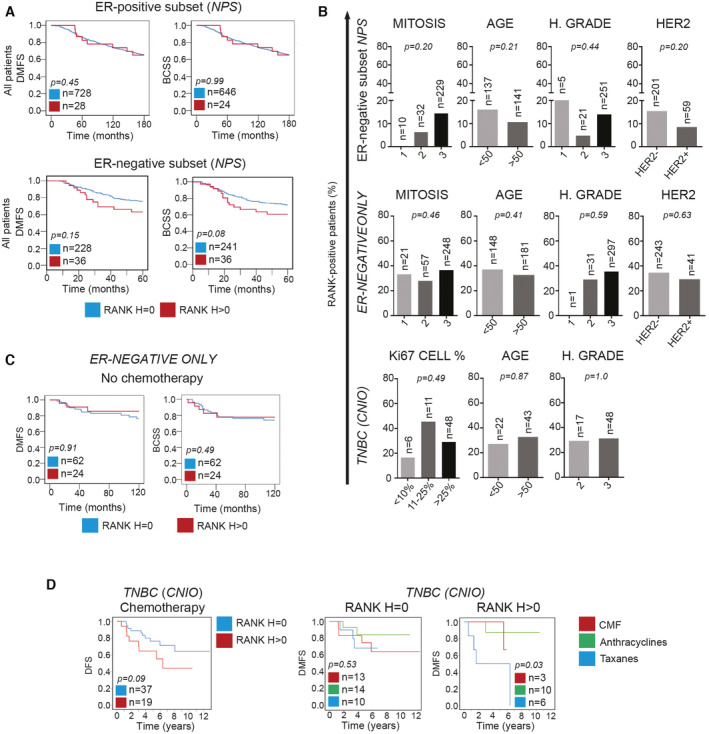
RANK tumor expression is not related to any clinicopathologic factor in ER^−^ BC BCSS and DMFS in the ER^+^ and ER^−^ subsets from the *NPS* collection according to RANK expression in all patients.Percentage of RANK^+^ BC patients according to the indicated clinicopathologic parameters in the three ER^−^ collections analyzed: *NPS* ER^‐^ subset, *ER‐NEGATIVE ONLY* and *TNBC (CNIO)*. Total number of analyzed patients per parameter and *p‐*values (Pearson's Chi‐Square test (Exact Sig. 2‐side)) are indicated.DMFS and BCSS according to RANK expression in patients with ER^−^ tumors not treated with chemotherapy in the *ER‐NEGATIVE ONLY* collection.RANK expression and DMFS in RANK^−^ (H = 0) and RANK^+^ (H > 0) tumor samples from the *TNBC (CNIO)* collection treated with chemotherapy and according to the chemotherapy regimen (group 1: CMF (cyclophosphamide, methotrexate, 5‐fluorouracil); group 2: FAC (5‐flurouracil, doxorubicin, cyclophosphamide) or FEC (5‐fluorouracil, epirubicin and cyclophosphamide); and group 3: CMF or FAC or FEC plus taxanes). Data information: (A, C, D) Total number of analyzed patients per parameter and *p‐*values (Log‐rank test (Mantel‐Cox)) are indicated.

Given the differences in prognosis between ER^+^ and ER^−^ BC and the increased RANK^+^ in ER^−^ BC, we assessed the significance of tumor RANK^+^ separately in both subsets. RANK positivity did not associate with survival in the *NPS* ER^+^ patients, but in the *NPS* ER^−^ subset, there was a trend toward worse DMFS and BCSS. RANK did not associate with any of the clinicopathologic factors analyzed (Dataset EV1; Fig EV3A and B). Although no associations between RANK^+^ and HER2^+^ were found, RANK expression associated with poor survival in HER2^+^ tumors, but the small sample size prevented solid conclusions (Dataset EV1).

**Figure 2:**
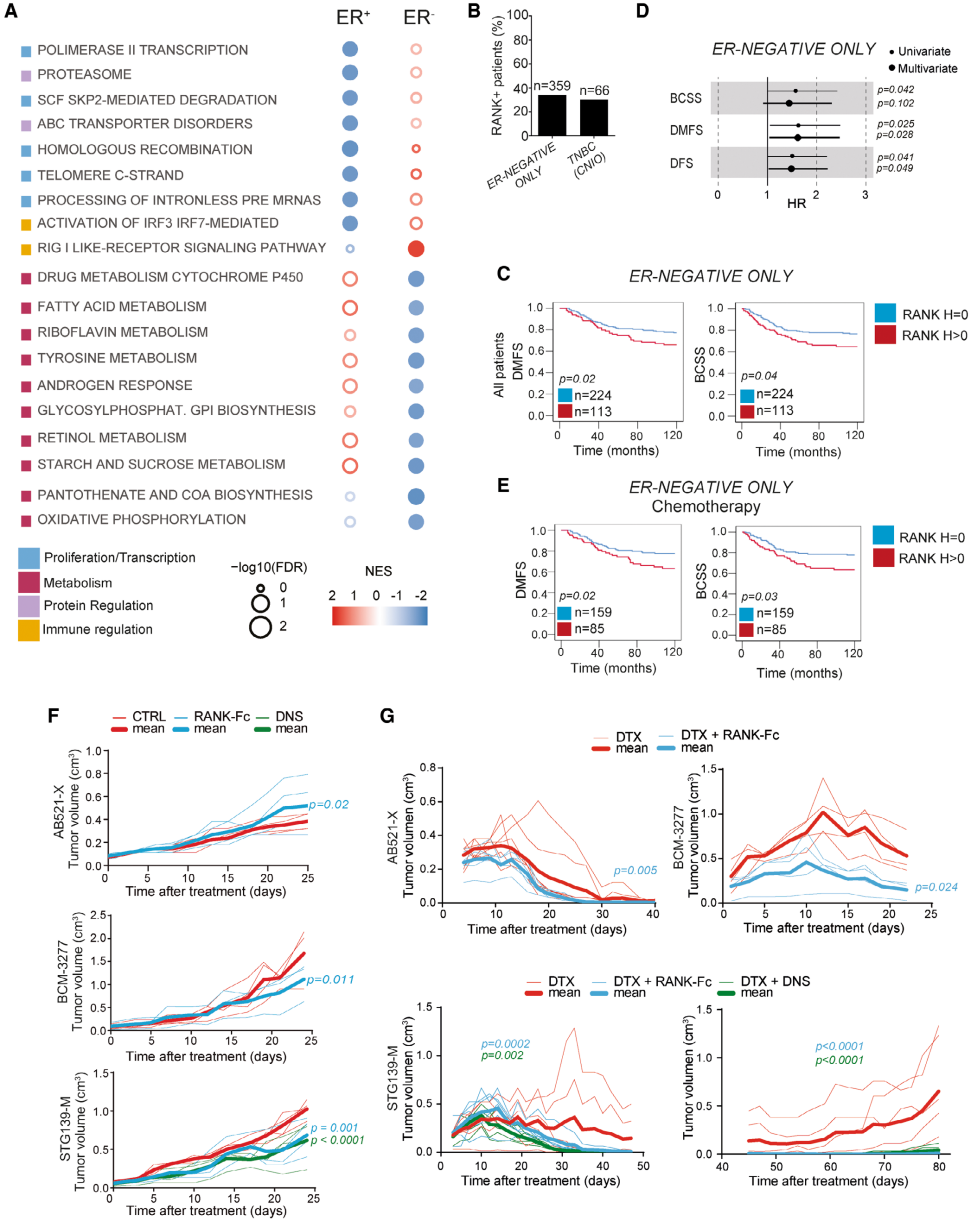
RANK tumor expression associates with poor survival in ER^−^ BC and RANKL inhibition improves response to chemotherapy ABubble matrix represents GSEA results of pathways associated with RANK protein expression in the *METABRIC* collection classified by ER expression. The matrix illustrates the NES and FDR values (empty bubbles FDR > 0.25). Color legend indicates the main biological process associated.BPercentage of RANK^+^ tumors in indicated ER^−^ collections.CDMFS and BCSS according to RANK expression.DForest plots showing HR, 95% CI and uni or multivariate *p*‐values for the indicated survival parameters.EDMFS and BCSS after chemotherapy (anthracyclines/taxanes) according to RANK expression.F, GTumor growth curves ((π × length × width^2^)/6) of the indicated PDXs after treatment with RANK‐Fc, denosumab (DNS) or mock (CTRL), alone (F) or in combination with docetaxel (DTX) (G). (G) Bottom left shows tumor growth/regression during DTX/RANKL‐inhibitor treatment for STG139‐M. Bottom right shows the tumor relapse in these same mice after removal of the treatment. Each thin curve represents one single tumor, and each thick curve represents the mean of all tumors implanted. Linear regression analysis and two‐tailed *p‐*value are shown. Data information: (B, C, E) Total number of analyzed patients per parameter and *p‐*values (Log‐rank test (Mantel‐Cox)) are indicated.

Thanks to the availability of gene expression data from the *METABRIC* dataset, we identified the pathways differentially regulated in RANK^+^ tumors: 67 in ER^+^ and 17 in ER^−^ BC patients, with no overlap between them (FDR < 0.25). In ER^+^ BC, RANK associated with replication/transcription, while in ER^−^ BC, RANK seemed to modulate multiple metabolic processes (NES < 0) (Fig 2A; Dataset EV4). Several of these pathways were regulated upon RANKL treatment in the ER^−^ PDX models, indicating direct regulation by RANK signaling (Dataset EV3, Venn in Dataset EV4). Altogether, these results highlight the different biology of RANK signaling according to ER status, which may contribute to the differences in prognosis observed between RANK^+^ ER^+^ and ER^−^ BC.

### RANK expression in ER ^−^ tumors associates with poor survival and response to chemotherapy

As the frequency of RANK positivity in the *NPS* collection was low (13%), we analyzed two additional and more recent collections of ER^−^ tumors: the *ER‐NEGATIVE ONLY* collection (*n* = 359 ER^−^ tumors) and the *TNBC (CNIO)* collection (*n* = 66). Tumor RANK^+^ was found in 34 and 30.3% of the samples, respectively (Fig 2B), in line with previous reports (Palafox *et al*, 2012; Pfitzner *et al*, 2014). Again, while tumor RANK expression was not associated with any of the clinicopathologic factors analyzed (Fig EV3B; Dataset EV1), in the *ER‐NEGATIVE ONLY* collection, patients with RANK^+^ tumors showed a significant poorer 10‐year survival compared with patients with RANK^−^ tumors (Fig 2C), confirming the results of the *NPS* ER^−^ subset (Fig EV3A). Indeed, RANK expression was an independent factor of worse DMFS and DFS in ER^−^ patients (*ER‐NEGATIVE ONLY*; Fig 2D; COX Dataset EV1). When HER2 expression, BRCA1 mutations and basal markers were considered, sample size was too small to get solid conclusions, although association of RANK^+^ with poor DFS in HER2^+^ ER^−^ tumors was observed (Dataset EV1).

Patients with ER^−^ RANK^+^ tumors showed poorer survival after adjuvant chemotherapy (mainly taxanes and anthracyclines) than those lacking RANK (Fig 2D), while no survival differences associated with RANK were found in the absence of chemotherapy (Figs 2E and EV3C; Dataset EV1). Similarly, in the *TNBC (CNIO)* collection tumors expressing RANK tended to have worse survival in patients receiving chemotherapy, particularly to regimens containing taxanes (Fig EV3D; Dataset EV1). Altogether, these results point out the importance of RANK expression as an independent biomarker of both poor prognosis and chemotherapy response in ER^−^ BC.

### RANKL inhibition improved response to docetaxel in ER ^−^ RANK ^+^ BC PDXs

**Figure EV4:**
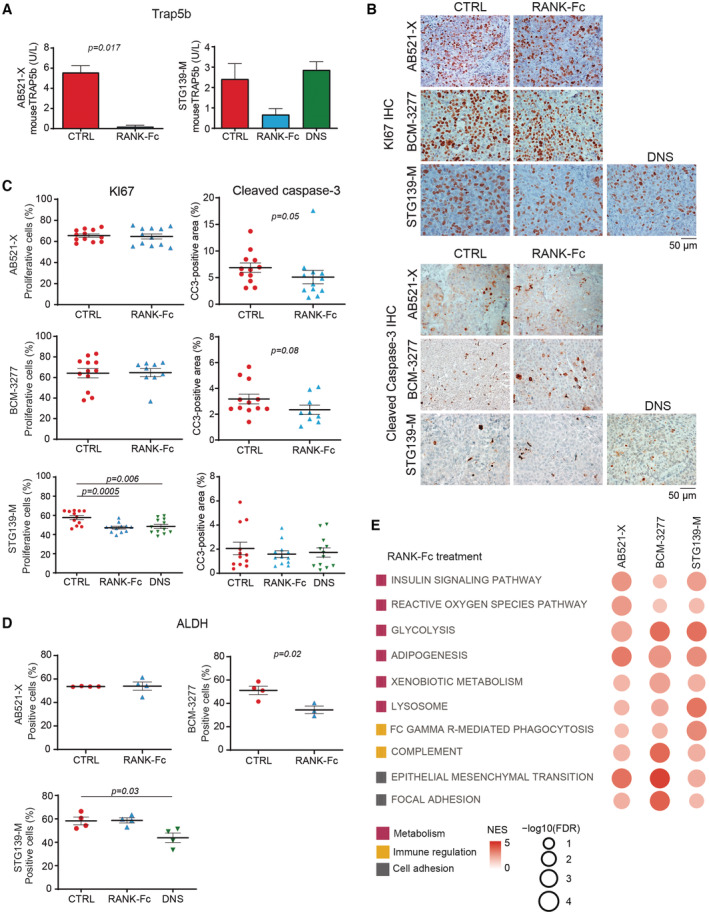
RANKL inhibition in BC PDXs ATrap5b levels in mouse serum (*n* = 2) in tumor‐bearing PDXs treated *in vivo* with RANK‐Fc or DNS. Mean ± SD is shown.B, CRepresentative images (B) and quantification (C) of KI67 and cleaved caspase‐3 staining measured by IHC in RANK^+^ tumors of PDXs treated *in vivo* with RANK‐Fc or DNS. Each dot represents one picture. Three representative pictures per tumor were quantified and at least 3–4 tumors per condition were analyzed. Mean ± SEM and two‐tailed *t*‐test *p‐*values are shown.DPercentage of cells with ALDH activity in tumors isolated from the indicated PDXs. Each dot represents one tumor. Mean ± SEM and *p‐*value of two‐tailed *t*‐student test are shown.EGene set enrichment analysis (GSEA) of associated genes after *in vivo* treatment with RANK‐Fc in NSG mice, which are common for the 3 PDX models studied. The matrix illustrates NES and FDR values. The color scale represents the NES. The size of the bubbles is proportional to the ‐log10 of the FDR. For those signatures with an FDR = 0 after 1,000 permutations, we assigned an FDR = 10^‐3 for visualization purposes. The signatures selected for this plot belong to Hallmark, Biocarta, Reactome and KEGG collections and have a reported FDR < 0.05 and a NES > 0 for all PDX models. The color legend indicates the main biological process associated with each signature. Data information: (A–D) All the analysis were performed 24 h after last treatment.

Our clinical results prompted us to evaluate whether RANKL therapeutic inhibition *in vivo* would impact the growth of the ER^−^ RANK^+^ BC PDX. Tumor‐bearing mice were randomized for treatment with the RANKL inhibitors RANK‐Fc or denosumab (the latter used only in the STG139‐M model as it expresses hRANKL) or mock (control). Serum levels of the bone remodeling marker, Trap5b, decreased upon RANK‐Fc, confirming the efficacy of the treatment, but not after denosumab as it only binds to human RANKL (Fig EV4A). RANK‐Fc and denosumab decreased tumor cell proliferation and attenuated tumor growth in the STG139‐M model, suggesting that the anti‐proliferative effects were mainly due to inhibition of tumor RANKL (Figs 2F and EV4B and C), in line with (Gonzalez‐Suarez *et al*, 2010). Tumor apoptosis was comparable between groups (Fig EV4B and C) and RANKL inhibition reduced ALDH activity in BCM‐3277 and STG139‐M (Fig EV4D), supporting a reduction in stemness (Yoldi *et al*, 2016).

Transcriptomic analyses upon RANKL inhibition revealed changes in tumor metabolism, immunity, adhesion, among others, with > 100 pathways (FDR < 0.25) shared between the three PDXs (Fig EV4E; Dataset EV3, Table P). Denosumab behaved as RANK‐Fc in STG139‐M (Venn in Dataset EV3, Table Q). The genetic signature obtained in DNS‐treated patients from the D‐BEYOND clinical trial (NCT01864798; Gómez‐Aleza *et al*, 2020), was associated with RANKL inhibition in the three RANK^+^ PDXs (Dataset EV3, Table R). Moreover, pathways such as fatty acid metabolism and oxidative phosphorylation were shared between the three RANK‐Fc‐treated PDXs and those associated with RANK expression in the ER^−^ *METABRIC* samples (Venn in Dataset EV4). This reinforces the clinical relevance of the PDX BC models and the pleiotropic effect of RANKL inhibitors in BC. Finally, in line with the association between tumor RANK expression and the poor response to chemotherapy observed in clinical samples, increased benefit was observed when RANKL inhibitors were combined with docetaxel in the three PDXs (Fig 2G). In the STG139‐M model, the combination led to complete tumor regression and no tumor relapse after interruption of docetaxel (Fig 2G). These results demonstrate that inhibition of RANK signaling improves response to chemotherapy in ER^−^ BC.

### RANK expression associates with poor prognosis in postmenopausal BC

**Figure 3:**
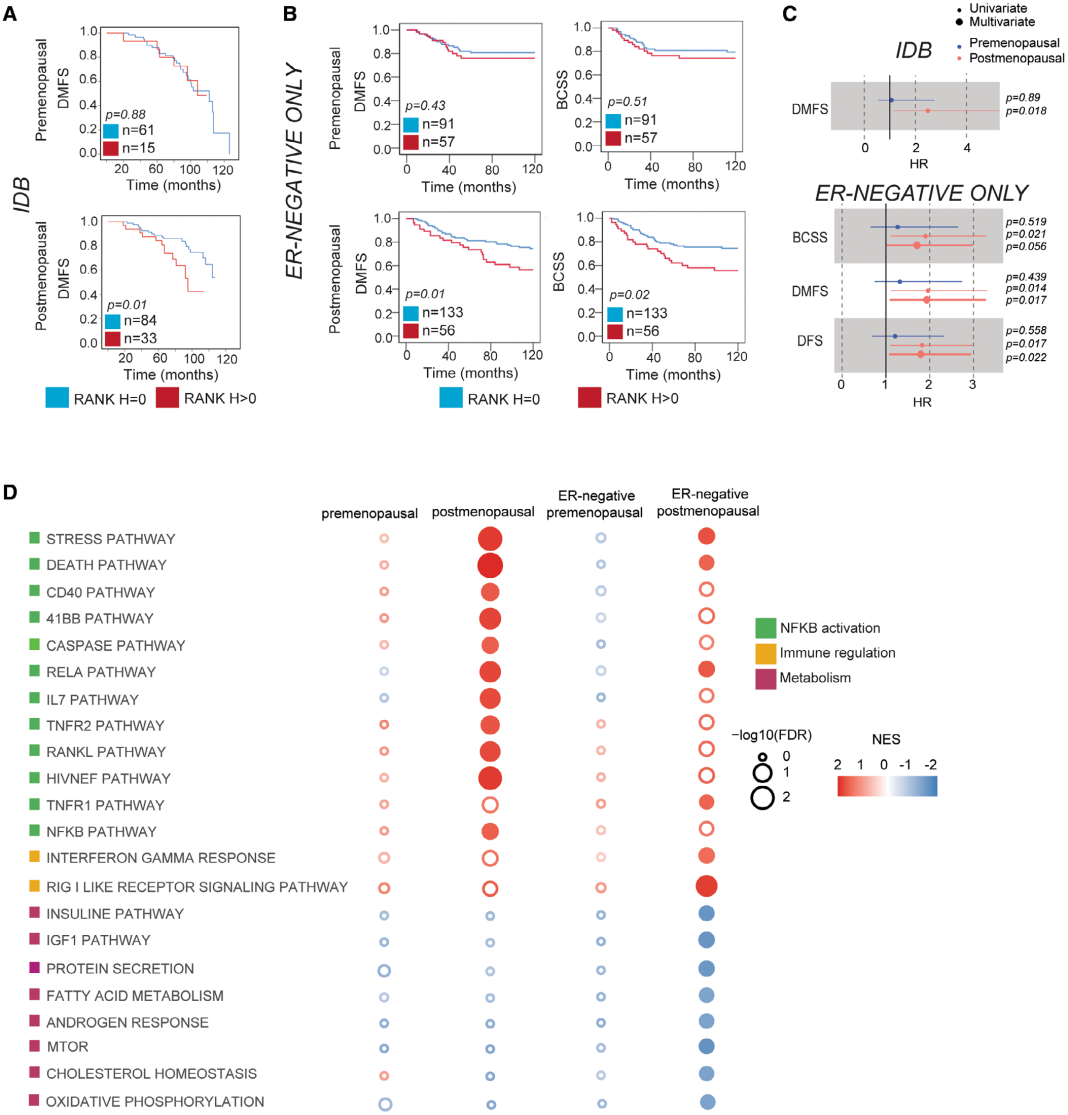
Tumor RANK expression associates with poor survival in postmenopausal patients and RANK biology in BC changes with menopause A, BDMFS and BCSS according to RANK expression in premenopausal and postmenopausal patients. Total number of analyzed patients per parameter and *p‐*values (Log‐rank test (Mantel‐Cox)) are indicated.CForest plots showing HR, 95% CI and uni or multivariate *p*‐values for the indicated survival parameters.DBubble matrix represents GSEA results of pathways associated with RANK protein expression in all patients and in the ER^−^ subset from the *METABRIC* collection classified by menopausal status. The matrix illustrates the NES and FDR values (Empty bubbles = FDR > 0.25). Color legend indicates the main biological process associated.

**Figure EV5:**
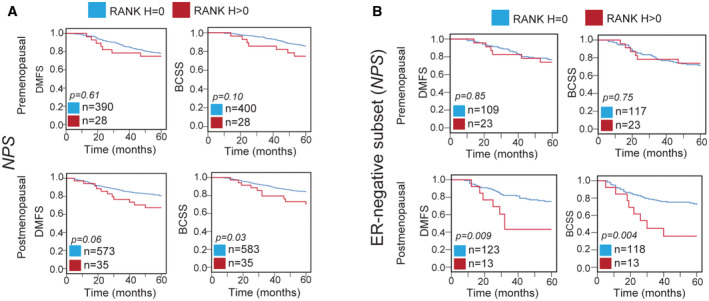
RANK is a marker of poor prognosis in BC after menopause A, BDMFS and BCSS according to RANK expression (RANK^−^ (H = 0) or RANK^+^ (H > 0)) in premenopausal and postmenopausal patients of the *NPS* collection (A) and the *NPS* ER^−^ subset (B).

Finally, we evaluated whether the prognostic value of RANK expression in BC would change with menopause, as RANK pathway is regulated by sex hormones. Interestingly, RANK^+^ associated with poor survival in postmenopausal, but not in premenopausal patients from the general *IDB* (DMFS) and *NPS* (BCSS) collections (Figs 3A and EV5A; Dataset EV1). Multivariate analyses showed that RANK^+^ associated with worse BCSS and DMFS only in postmenopausal women (*NPS*) (Dataset EV1, COX *NPS*). These findings were also validated in ER^−^ patients. RANK expression was an independent factor of worse DMFS and DFS in postmenopausal, but not in premenopausal patients in tumors from *ER‐NEGATIVE ONLY* collection (Fig 3B and C; Dataset EV1). Even in the *NPS* ER^−^ subset, RANK^+^ associated with worse DMFS and BCSS in postmenopausal patients (Fig EV5B; Dataset EV1). Results from three independent collections demonstrated that RANK expression is an independent biomarker of poor prognosis in postmenopausal BC.

GSEA revealed that tumor RANK expression in postmenopausal cases was positively associated (FDR < 0.25) with 20 pathways, 12 of them related to TNF/NFKB signaling, including RANKL pathway itself, while in premenopausal tumors, only three pathways were associated with RANK^+^ (Fig 3D; Dataset EV4), suggesting that, similar to the bone, RANK signaling in BC is more active after menopause (Streicher *et al*, 2017). Meanwhile, in postmenopausal ER^−^ tumors, RANK^+^ was positively associated with NFKB activation and immunity. Negative associations with multiple metabolic pathways (insulin/IGF1 signaling, fatty acid metabolism, mTOR, cholesterol homeostasis and oxidative phosphorylation) were found (Fig 3D; Dataset EV4). Again, these same pathways were modulated by RANK signaling in the PDX (Fig EV4E; Venn in Dataset EV4). These findings suggest that RANK activation in BC increases after menopause, regulates tumor cell metabolism and hence, contributes to the association of RANK expression with poor prognosis in ER^−^ postmenopausal BC.

## Discussion

Great heterogeneity persists in all BC subtypes that translates into wide‐range of responses to current, and still limited, treatments. Searching for new prognostic and predictive factors has become an essential task for the individualization of BC therapy (Weigel & Dowsett, 2010).

In this work, the analyses of RANK and RANKL in more than 2,000 BC samples from four independent TMA cohorts, confirmed that RANK expression was associated with ER^−^ tumors while RANKL was rarely found in tumor cells (Palafox *et al*, 2012; Pfitzner *et al*, 2014). The large number of samples analyzed in our study allowed to define RANK expression as an independent poor prognosis factor in BC, in particular in ER^−^ BC and in postmenopausal women. The distinct biology associated with RANK signaling according to ER status may explain why RANK predicts poor prognosis in ER^−^, but not in ER^+^ BC. RANK protein expression in ER^+^ tumors was negatively associated with proliferation, in line with its association with senescence in luminal tumors (Benítez *et al*, 2021). However, additional ER^+^ collections need to be evaluated to determine the prognostic value of RANK in ER^+^ BC, as the low frequency of RANK positivity in the *NPS* collection is a limitation.

Transcriptomic analyses in ER^−^ tumors and PDXs upon modulation of RANK signaling evidence its pleiotropic role in BC, regulating multiple biological processes with a key role in tumor metabolism and immunity (Gonzalez‐Suarez *et al*, 2010; Yoldi *et al*, 2016; Rao *et al*, 2017; Gómez‐Aleza *et al*, 2020). Despite BC heterogeneity, a strong overlap of RANK‐driven pathways was found between the different PDXs and clinical samples, which may help define a signature to select BC patients who may benefit from denosumab and the evaluation of drug response during treatment.

Our results suggest that RANK^+^ ER^−^ tumors showed a worse response to chemotherapy regimens that include taxanes. Accordingly, increased therapeutic benefit was observed in the ER^−^ PDXs when RANKL inhibitors were combined with chemotherapy. Results from the GeparX clinical trial demonstrated that neoadjuvant denosumab in combination with nab‐paclitaxel did not increase the pathological complete response in patients with early BC, not even in patients with RANK^+^ early BC, but survival remains to be evaluated (Blohmer *et al*, 2022).

Paradoxically to the well‐characterized role of RANK signaling as a mediator of progesterone in healthy breast or preneoplasic lesions (Gonzalez‐Suarez *et al*, 2010; Schramek *et al*, 2010), our results demonstrate that RANK predicts poor prognosis in ER^−^ postmenopausal BC. The drop of estrogen levels leads to increased RANK signaling in the bone and osteoporosis (Streicher *et al*, 2017). Similarly, RANK signaling seems to increase after menopause in breast tumors, as multiple pathways related to NFKB activation, including RANKL pathway, were positively associated with RANK protein expression only in postmenopausal patients. These results suggest that denosumab would show the highest therapeutic benefit in postmenopausal women with ER^−^ RANK^+^ breast tumors. The meta‐analysis by the Early Breast Cancer Clinical Trialists' Collaborative Group supports the idea that adjuvant treatment of early BC might be more efficacious with the addition of a bone‐modifying agent, particularly in postmenopausal women, or in combination with ovarian function suppression (Chukir *et al*, 2019; Perrone & Gravina, 2020). The increased bone remodeling and the metabolic changes (systemic and in the tumor) that follow the drop in estradiol levels (Khosla *et al*, 2012) may also contribute to the role of RANK as a marker of poor survival after menopause.

Results from the ABCSG18 trial revealed that adjuvant denosumab reduced the risk of bone fractures and improved DFS of ER^+^ postmenopausal BC patients (Gnant *et al*, 2018), but this was not validated in the D‐CARE trial (Coleman *et al*, 2020). However, in these trials, RANK expression or RANK pathway activation was not considered. Retrospective analyses of RANK pathway expression/activation are required for evaluating BC outcome after denosumab treatment.

In summary, we demonstrate that RANK is an independent marker of poor prognosis in ER^−^ BC after menopause and our functional analyses support the therapeutic potential of RANK pathway inhibitors in ER^−^ postmenopausal BC.

## Materials and Methods

### Tissue microarray (TMA) staining and scoring

RANK and tmRANKL expression were evaluated in TMAs from five different cohorts of BC patients. *IDB* TMA (donated by A. Sierra (IDIBELL, Spain)), contains 404 BC samples and clinicopathologic information from 314 patients (24–88 years old) diagnosed between 1989 and 2009. Follow‐up ranged from 8 to 146 months (mean: 76.6 months). Metastasis relapse occurred in 43.4% (138 of 318) of patients; of these, 84 patients (60.9%) developed brain metastasis, 47 (34.1%) lung metastasis, 54 (39.1%) liver metastasis, 40 (29.0%) nonregional lymph node metastasis and 89 (64.5%) bone metastasis. Just over half (56.6%; 180 of 318) of the patients had no metastatic progression after a minimum follow‐up of 5 years. *NPS* TMA is a well‐characterized cohort of unselected early‐stage (I–III) primary operable invasive BC from patients aged 70 years or younger, enrolled into the Nottingham Tenovus Primary Breast Carcinoma Series between 1990 and 1997 (*n* = 1,895), and managed in accordance with a uniform protocol; a subset of cases (*n* = 298) were included in the *METABRIC* study (Curtis *et al*, 2012), where gene expression data is available. Outcome data include survival status, survival time, cause of death, development, and time to locoregional recurrence and distant metastasis (DM). BCSS is defined as the time (in months) from the date of primary surgery to the date of breast cancer‐related death. DMFS is defined as the time (in months) from the date of primary surgery to the appearance of DM. Treatments include chemotherapy (cyclophosphamide, methotrexate and fluorouracil (CMF)) or endocrine therapy. At that time patients with HER2^+^ tumors had no access to trastuzumab. Positive ER status was defined as > 1% of tumor cells expressing ER. Positive HER2 status was defined using immunohistochemistry as HER2 3+. Histological grade was assessed based on the Nottingham Grading System (Elston & Ellis, 1991; Rakha *et al*, 2008). Other clinicopathologic factors such as ER, PR and/or HER2 expression, proliferation rate (KI67 expression or mitosis), vascular invasion, as well as patient age and survival analysis were analyzed before including the samples into the TMAs (Abd El‐Rehim *et al*, 2005). Two additional collections of ER^−^ tumors were analyzed, the Nottingham *ER‐NEGATIVE ONLY* cohort (1998–2006), which contains 396 samples, and the *TNBC* (*CNIO)*, a small collection of 66 TNBC patients with 40–50% of relapse, generated by Dr M. Quintela‐Fandino (CNIO, Spain). In the *ER‐NEGATIVE ONLY* cohort the chemotherapy regimen used was CMF and after year 2000 anthracyclines plus taxanes. In the *TNBC (CNIO)* cohort, three regimens were used: group 1 CMF (cyclophosphamide, methotrexate, and 5‐fluorouracil), group 2 FAC (5‐fluorouracil, doxorubicin, and cyclophosphamide) or FEC (5‐fluorouracil, epirubicin, and cyclophosphamide), and group 3 CMF or FAC or FEC plus taxanes.

RANK or tmRANKL staining was scored for intensity (on a scale of 0 to 3; 0 = no staining, 1 = weak, 2 = moderate, 3 = intense) and positive cell percentage (on a scale of 0 to 100%) within tumor cells or surrounding stroma for each TMA core sample. The H‐Score value is defined as the sum of multiplying staining intensity by positive area, ranging from 0 to 300. TMA cores with less than 30% of the core area were discarded. Patients were stratified according to RANK or tmRANKL H‐Scores as being protein‐positive (H > 0) or protein‐negative (H = 0). As TMA samples are enriched in tumor cells, the stroma content was not always present or representative and H‐Score was not calculated. Total number of scorable samples for each of the collections and stainings are indicated in the corresponding fig.

### PDX models

Generation of the IDB PDXs is described by Gómez‐Miragaya *et al* (2017). Briefly, they were generated by orthotopic transplantation of human fresh tumor tissue or injection of metastatic cancer cells isolated from pleural effusions into the cleared mammary fat pad of immunodeficient female mice (NOD.Cg‐Prkdcscid Il2rgtm1Wjl/SzJ, RRID:IMSR_JAX:005557, The Jackson Laboratory). The rest of the PDXs were obtained through collaboration with Dr V. Serra and Dr J. Arribas (Vall d'Hebron Institute of Oncology), Dr A. Welm (Huntsman Cancer Institute), Dr M. T. Lewis (Baylor College of Medicine), Dr A. Bruna and Dr C. Caldas (Cancer Research UK Cambridge Institute) and Dr R. Clarke (Manchester Breast Centre).

PDXs were maintained by consecutive rounds of transplantation of tumor pieces. In the case of the BCM‐3277 model, mice were treated with 17β‐estradiol at 8 μg/ml (Sigma) in drinking water. Mice were kept in individually ventilated and open cages and food and water were provided *ad libitum*. Cages, bedding, food and water were all autoclaved. Euthanasia was performed by CO_2_ inhalation. All animal experiments were conducted according to institutional policies and national and European guidelines.

### Tumor cell isolation

Single cells were isolated from tumors as described previously (Smalley, 2010). Briefly, fresh tissues were mechanically dissected with a McIlwain tissue chopper and enzymatically digested with appropriate medium (DMEM F‐12, 0.3% collagenase A, 2.5 U/ml dispase, 20 mM HEPES, and 100 U/ml penicillin/100 μg/ml streptomycin) 60 min at 37°C. Samples were washed with Leibowitz L15 medium/10% FBS between each step. Erythrocytes were eliminated with hypotonic lysis buffer, and fibroblasts were excluded by incubation with DMEM F‐12/10% FBS 1 h at 37°C. Single epithelial cells were isolated by treating with trypsin 2 min at 37°C. The cell suspension was filtered with 40 mm cell strainers and counted.

### RANKL, RANK‐Fc, denosumab and docetaxel treatments *in vivo*

Dissociated tumor cells mixed 1:1 with Matrigel Basement Membrane (BD Biosciences) were transplanted orthotopically in the inguinal mammary gland of 10–/12‐week‐old NSG mice and when tumors reached 5 mm of diameter mice were randomized for mock, h‐RANKL (0.75 mg/kg, 4–6 doses, twice per week; Amgen Inc.), h‐RANK‐Fc treatment (10 mg/kg, three times per week; Amgen Inc.) or denosumab (10 mg/kg, three times per week; XGEVA^®^). Docetaxel (Hospira/Actavis, 20 mg/kg) was administered once per week together with dexamethasone (0.132 mg/kg, Merck), to reduce the chemotherapy‐induced inflammation. All the drugs were administered intraperitoneally. Tumor growth was monitored and measured with a caliper once per week. Tumor volume was then calculated as follows: π × length × width^2^/6 in cm^3^. Treatment was interrupted when tumors regress below 3 mm of diameter. Mice were sacrificed and tumors were surgically removed 24 h after treatment completion. In combination treatments with docetaxel, mice were sacrificed once relapsing tumors reached 10 mm of diameter.

### Flow cytometry

Single tumor cells were resuspended and incubated in blocking solution (PBS containing 2% FBS, 2 mM EDTA and IgG blocking reagent (Sigma)) for 10 min on ice. Mouse cells were excluded in flow cytometry using H2Kd‐PECy7 (SF1‐1.1, 116622 from BioLegend). Gating was based on “Fluorescence Minus One” controls. ALDH activity of tumor cells was assessed using the ALDEFLUOR™ Kit (01700 from STEMCELL Technologies), following the manufacturer's protocol. Live/dead staining was performed using DAPI (Thermo Fisher Scientific). A population of 10,000 alive cells was acquired in all experiments. Samples were analyzed using a Gallios flow cytometer (Beckman Coulter) and the FlowJo software.

### Enzyme‐linked immunosorbent assay (ELISA)

Trap5b activity was measured in mouse serum according to the manufacturer's instructions (IDS).

### Tissue histology and immunostaining

Three micrometer sections was cut and immunohistochemistry of hRANK and hRANKL was performed as described (Gonzalez‐Suarez *et al*, 2010). RANK antigen retrieval was carried out with the Diva Decloaker buffer (Biocare Medical), 90°C, 14–16 h; sodium citrate buffer (0.01 M, pH = 6) was used for RANKL. Protein blocking was done with TNB Blocking Buffer (PerkinElmer). Anti‐human RANK monoclonal antibody (N1H8; Amgen Inc., 5 μg/ml), anti‐human RANKL monoclonal antibody (Amgen Inc., M366, 1.85 μg/ml), anti‐KI67 (SP6, Abcam; 1:200) and anti‐cleaved caspase‐3 (Asp175, Cell Signaling; 1:200) antibodies were used. VECTASTAIN^®^ Elite^®^ ABC‐HRP Kit (Vector Laboratories) was used to amplify the RANK, RANKL and cleaved caspase‐3 signal. Images were analyzed with Fiji software (Schindelin *et al*, 2012).

### RANKL stimulation *in vitro*

PDX single tumor cells were embedded in Corning™ Matrigel™ Growth Factor Reduced Basement Membrane Matrix (Corning™ 356,238), plated in DMEM/F‐12 with B‐27™ Supplement, EGF 10 ng/ml, hydrocortisone 0.5 μg/ml, insulin 5 μg/ml, cholera toxin 100 ng/ml, and penicillin/streptomycin. Cells were stimulated, or not, with h‐RANKL (500 ng/ml; Amgen Inc.) during 24 h prior to gene expression analyses.

### Quantitative reverse transcription PCR (Q‐RT‐PCR)

Total RNA was isolated from tumor pieces using TRIzol (Thermo Fisher Scientific) or Maxwell^®^ RSC simplyRNA Tissue Kit (AS1340 Promega). One microgram of RNA was reverse‐transcribed into cDNA using 200 U Superscript II plus random hexamer oligos (Invitrogen). *RANK* and *RANKL* expression was amplified with LightCycler^®^ 480 Probes Master (Roche, 04707494001) and a LightCycler^®^ 480 thermocycler (Roche) and normalized relative to the *PPIA house keeper gene*. The primer sequences used were: *PPIA‐UPL* (Fw: ATGCTGGACCCAACACAAAT; Rv: TCTTTCACTTTGCCAAACACC), *TNFRSF11A‐UPL* (Fw: GCAGGTGGCTTTGCAGAT; Rv: GCATTTAGAAGACATGTACTTTCCTG), *TNFSF11‐UPL* (Fw: TGATTCATGTAGGAGAATTAAACAGG; Rv: GATGTGCTGTGATCCAACGA).

Gene expression in organoid cultures was evaluated using SYBR Green Master I (Roche, 04887352001), LightCycler^®^ 480 thermocycler (Roche). The primers used were: *PPIA* (Fw: ATGGTCAACCCCACCGTT; Rv: TCTGCTGTCTTTGGGACCTTG), *TNFRSF11A* (Fw: ATCTGGGACGGTGCTGTAAC; Rv: GGCCTTGCCTGTATCACAAA), *TNFSF11* (Fw: TGATTCATGTAGGAGAATTAAACAGG; Rv: GATGTGCTGTGATCCAACGA), *BIRC3* (Fw: GGTAACAGTGATGATGTCAAATG; Rv: TAACTGGCTTGAACTTGACG), *ICAM1* (Fw: AACTGACACCTTTGTTAGCCACCTC; Rv: CCCAGTGAAATGCAAACAGGAC), *CCL2* (Fw: AGGTGACTGGGCATTGAT; Rv: GCCTCCAGCATGAAAGTCT), *CXCL8* (Fw: CTGCGCCAACACAGAAATTA; Rv: CATCTGGCAACCCTACAACA), *RELB* (Fw: CCCGACCTCTCCTCACTCTC; Rv: CAGGGTGACCGTGCTCAG), *NF‐kB2* (Fw: GGCGGGCGTCTAAAATTCTG; Rv: TCCAGACCTGGGTTGTAGCA).

### Western blot

PDX‐derived cells were seeded in growth medium (5% FBS, EGF 10 ng/ml, hydrocortisone 0.5 μg/ml, insulin 5 μg/ml, cholera toxin 100 ng/ml, and penicillin/streptomycin) overnight. The following day, cells were serum starved (growth medium containing 0.5% FBS) for 24 h before RANKL stimulation (500 ng/ml; Amgen Inc.). Extracts for immunoblots were prepared with modified RIPA buffer (50 mM Tris pH 7.4, 150 nM NaCl, 1% Triton NP‐40, 0.25% sodium deoxycholate) containing PhosSTOP and Complete protease inhibitor cocktail (Roche). Protein concentration was measured with DC protein assay reagent (Bio‐Rad) and 40 μg of total protein were resolved by SDS–PAGE and transferred to Immobilon‐P 0.45 μm membranes (Millipore). Primary antibodies against P‐p65 (Ser536, Cell Signaling: 1:500), p65 (D14E12, Cell Signaling; 1:1,000), P‐IkBα (S32/36, Cell Signaling; 1:500), IkBα (L35A5, Cell Signaling; 1:500), and β‐tubulin (ab21058, Abcam; 1:5,000) were used. Blots were incubated with HRP‐conjugated secondary antibodies (DAKO) and developed with ECL detection kit (Amersham Biosciences).

### RNA sequencing

Total RNA samples were processed with the “QuantSeq 3′ mRNA‐Seq Library Prep Kit (FWD) for Illumina” (Lexogen, Cat.No. 015) with RNA Quality scores of 7.7 on average (range 4.2–9.2). Library generation was initiated by reverse transcription with oligodT priming, and a second strand synthesis was performed from random primers. Libraries were completed by PCR. cDNA libraries were purified, applied to an Illumina flow cell for cluster generation and sequenced on an Illumina instrument. Read adapters and poly A tails were removed with BBDuk v38.38. Then, human reads were separated from mice ones using Xenome v1.0.1 (Conway *et al*, 2012) and those classified as “human,” “both” or “ambiguous” were selected. Processed reads were analyzed with the Nextpresso pipeline v1.9.2.5 (Graña *et al*, 2018). Sequencing quality was checked with FastQC v0.11.7 and FastQ Screen v0.13.0. Reads were aligned to the human reference genome (GRCh38) with TopHat v2.0.10 using Bowtie v1.0.0.0 and Samtools v0.1.19.0 (−library‐type fr‐secondstrand), allowing three mismatches and 20 multihits. Read counts were obtained with HTSeq‐count v0.6.1 (−‐stranded = yes) using the human gene annotation from GENCODE (gencode.v34.GRCh38.Ensembl100). Differential expression was performed with DESeq2, using a 0.05 FDR. Genes were ranked according to the log2 Fold Change and GSEAPreranked v2.2.2 was used to perform gene set enrichment analysis for Hallmark, Biocarta, Reactome and KEGG v7.1 signatures, setting 1,000 gene set permutations and a classic enrichment statistic. Only those signatures with significant enrichment levels (FDR q‐value < 0.25) were considered.

### Statistical analysis

Statistical analysis in the TMA collections was performed with the support of the IDIBELL and Nottingham University Statistical Assessment Services. Associations between IHC scores and clinicopathologic parameters were evaluated using Pearson's Chi‐Square test or Fisher's exact test. Tumor samples with less than 30% tumor cells were excluded from the analyses. RANK and RANKL were scored blindly to the tumor clinical and pathological characteristics. BCSS, DMFS and DFS were analyzed using the Kaplan–Meier function, Cox regression analyses and the log rank test. Data analyses of mouse experiments were performed using GraphPad Prism software version 8. Regression analysis of the growth curve mean for *in vivo* treatments was performed. Analysis of the differences between two conditions was performed with a two‐tailed Student's *t*‐test.

Bubble matrix plots were drawn using R (v4.0.3) and ggplot2 (v3.3.3). These plots represent the NES and FDR values reported by GSEA for some selected pathways in all tested comparisons. The color scale represents the NES: red denotes a NES > 0 and blue a NES < 0. The more intense the color, the more extreme the NES. In addition, the size of the bubble is proportional to the −log10 of the FDR. Thus, the bigger the dot, the smaller the FDR. Gene sets were classified according to Pearson's *R* coefficient generated by public gene set databases (KEGG, Biocarta, Reactome, and Hallmarks). For *in vivo* experiments, mice showing a non‐complete recovery after surgery were excluded from the study. Mice were randomized into the different treatment groups when the tumor reached 5 × 5 mm and tumor monitoring was done in a blinded fashion.

### Study approval

All human samples were obtained following institutional guidelines, study received approval form the corresponding institutional Ethics Committee, and the experiments conformed to the principles set out in the WMA Declaration of Helsinki and the Department of Health and Human Services Belmont Report. This work obtained ethics approval to use the human tissue samples by the corresponding institutional review boards: Greater Manchester Central Research Ethics Committee reference number 15/NW/0685 (Nottingham); Hospital Universitario 12 de Octubre, number 11/137 (CNIO) and Hospital Universitario de Bellvitge, PR166/11071/015. Informed consent was obtained from all individuals prior to surgery to use their tissue materials in research. Written informed consent for PDX generation was obtained from all subjects. All experimental animal procedures were performed according to Spanish regulations. All research involving animals was performed at the IDIBELL and CNIO animal facilities in compliance with protocols approved by the IDIBELL Committee on Animal Care and the Directorate‐General for Agricultural Production of the Ministry of Agriculture and Livestock Farming (PROEX_161.2/21), respectively, following national and European Union regulations.

**The paper explained**

1 **Problem**

The search for new prognostic factors and therapeutic targets has become an essential task for the individualization of breast cancer therapy. RANK signaling pathway has emerged as a new target for breast cancer based on compelling preclinical evidence. RANKL inhibition prevents or attenuates mammary tumor initiation, and induces tumor cell differentiation and an anti‐tumorigenic immune response in established tumors. However, in clinical trials the therapeutic benefit of the RANKL inhibitor denosumab in breast cancer, beyond its bone‐related effects, is unclear. Given the heterogeneity of breast cancer, a better understanding of RANK biology is needed to identify the patients who may benefit from denosumab.

**Results**

Here, we report the expression patterns of RANK and RANKL proteins in more than 2,000 breast tumor samples from independent collections, together with functional studies in breast cancer patient‐derived xenografts (PDXs). Our results demonstrate that RANK protein expression in tumor cells constitutes a new independent biomarker of poor prognosis in patients with ER^−^ tumors and in postmenopausal women. Accordingly, RANKL inhibition improves response to chemotherapy in ER^−^ BC PDXs, reducing recurrence. The distinct biology of RANK signaling according to ER expression and menopause enlighten these results: RANK activation increases in tumors after menopause and regulates tumor cell metabolism in ER^−^ disease.

**Impact**

Our findings identify RANK as a new biomarker of poor prognosis in postmenopausal women with ER^−^ breast tumors. These results will help to identify breast cancer patients who can benefit from denosumab in a personalized therapeutic strategy.

## Author contributions

**Marina Ciscar:** Data curation; formal analysis; investigation; methodology; writing – original draft; writing – review and editing. **Eva M Trinidad:** Data curation; formal analysis; investigation; methodology; writing – original draft; writing – review and editing. **Gema Perez‐Chacon:** Data curation; formal analysis; investigation; writing – original draft; writing – review and editing. **Hector Perez‐Montoyo:** Data curation; formal analysis; investigation; writing – review and editing. **Maria Jimenez:** Data curation; formal analysis; investigation; writing – original draft; writing – review and editing. **Mansour Alsaleem:** Formal analysis; writing – review and editing. **Maria J Jimenez‐Santos:** Formal analysis; writing – review and editing. **Adrian Sanz‐Moreno:** Data curation; formal analysis; writing – review and editing. **Andrea Vethencourt:** Data curation; formal analysis; writing – review and editing. **Michael Toss:** Formal analysis; writing – review and editing. **Anna Petit:** Data curation; formal analysis; writing – review and editing. **Maria Teresa Soler‐Monso:** Formal analysis; writing – review and editing. **Victor Lopez:** Data curation; writing – review and editing. **Jorge Gomez‐Miragaya:** Data curation; writing – review and editing. **Clara Gomez‐Aleza:** Data curation; writing – review and editing. **Lacey E Dobrolecki:** Resources. **Michael T Lewis:** Resources. **Alejandra Bruna:** Resources. **Silvana Mouron:** Resources. **Miguel Angel Quintela‐Fandino:** Resources. **Fátima Al‐Shahrour:** Formal analysis; writing – review and editing. **Antonio Martinez‐Aranda:** Resources. **Angels Sierra:** Resources. **Andrew R Green:** Formal analysis. **Emad Rakha:** Resources; formal analysis; writing – review and editing. **Eva Gonzalez‐Suarez:** Conceptualization; resources; data curation; formal analysis; supervision; funding acquisition; investigation; methodology; writing – original draft; project administration; writing – review and editing.

## Disclosure and competing interests statement

EG‐S has served on advisory boards for Amgen Inc. and has received honoraria and research funding from Amgen Inc. MTL is a Founder of, and an uncompensated Manager in StemMed Holdings L.L.C., an uncompensated Limited Partner in StemMed Ltd., and is a Founder of and equity stake holder in Tvardi Therapeutics. LED is a compensated employee of StemMed Ltd. Selected BCM PDX models described herein are exclusively licensed to StemMed Ltd., resulting in tangible property royalties to MTL and LED.

## Supporting information



Expanded View Figures pdf

Dataset EV1

Dataset EV2

Dataset EV3

Dataset EV4

PDF+

## Data Availability

RNAseq results have been deposited in GEO: GSE185513 study (https://www.ncbi.nlm.nih.gov/geo/query/acc.cgi?acc=GSE185513).
