# Supplementary material for: RANK is a poor prognosis marker and a therapeutic target in ER‐negative postmenopausal breast cancer
Source: EMBO Mol Med. 2023 Mar 7;15(4):e16715. doi: 10.15252/emmm.202216715 (PMC10086586; doi:10.15252/emmm.202216715)
Supplement: Supplementary file 1 — Expanded View Figures pdf [file EMMM-15-e16715-s003.pdf]

Expanded View Figures

Figure EV1. RANK is expressed in tumor and stromal cells of human BC and associates with ER/PR-negative tumors.

A Percentage of patients expressing RANK or tmRANKL in tumor and stromal cells in BC samples. The total number of patients is indicated.
B H-Score values of tumor RANK and tmRANKL from *IDB*, *NPS* collections and the *METABRIC* dataset. Mean ± SEM is shown.
C Percentage of BC patients with $RANK^+$ tumors according to the indicated clinicopathologic parameters in the *METABRIC* cohort. Total number of analyzed patients per parameter and *p*-values (Pearson's Chi-Square test (Exact Sig. 2- Side)) are indicated.

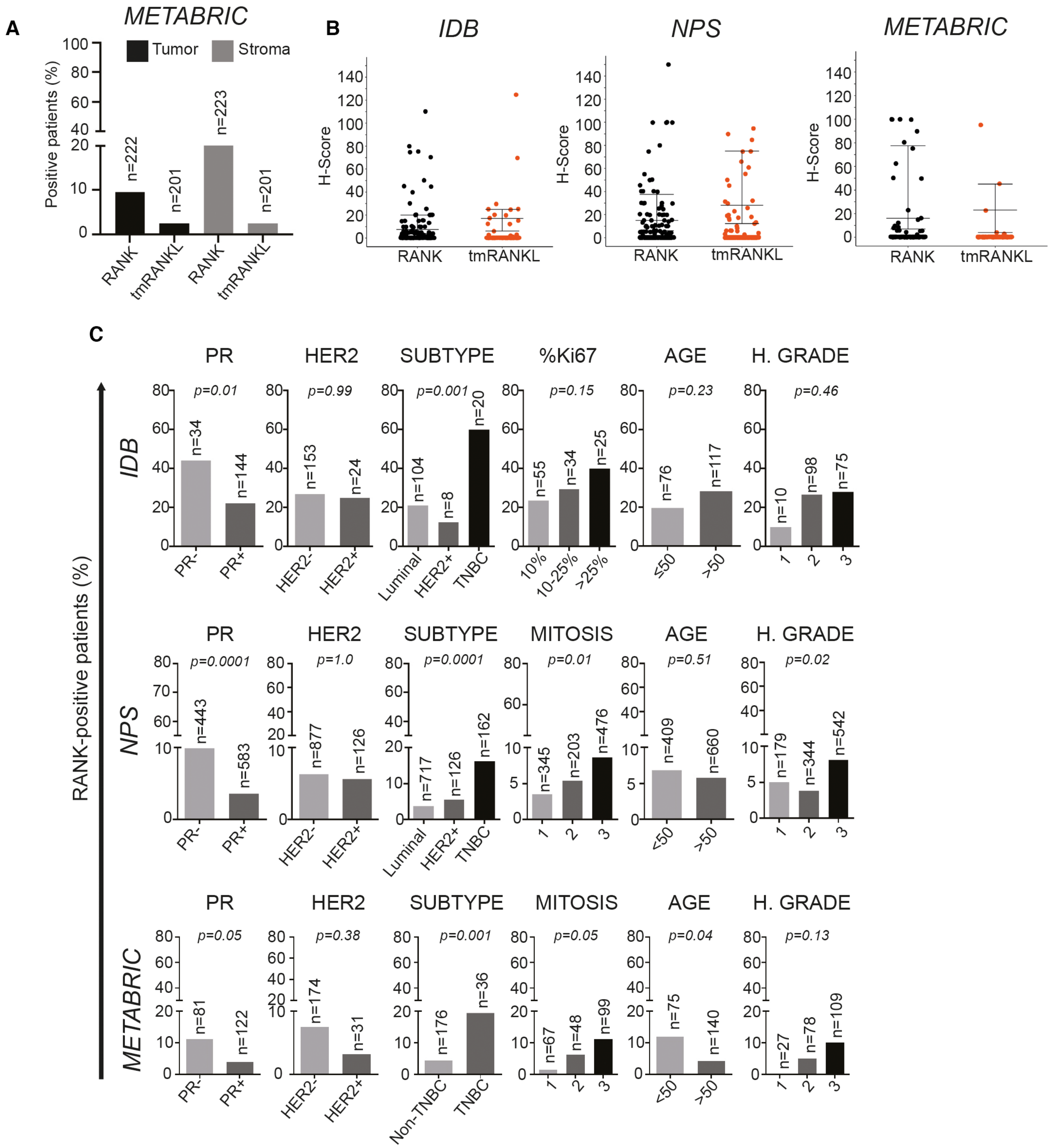

Figure EV1.

 © 2023 The Authors

Figure EV2. RANK is expressed and active in a subset of BC PDXs.

A RANK and RANKL mRNA expression levels relative to *PPIA* in the indicated BC PDXs, organized according to ER status in the human tumor of origin and *RANK* mRNA expression. Two-tailed *t*-student test was used to evaluate the RANK/RANKL differential expression between ER^{-} and ER^{+} BC PDXs. # Indicates models where RANK and RANKL expression were analyzed by IHC.

B Representative images of RANK and RANKL protein expression in BC PDXs detected by IHC. H-Score (H) of the models (and not of the picture) are shown. A total of 3–5 independent tumors per PDX were scored for RANK.

C Western blot analyses of P-p65, P-IKBα and corresponding total proteins after RANKL stimulation in the indicated PDXs. Tubulin was used as a loading control.

D Gene expression analyses of the indicated NFKB target genes in PDX tumor organoids after 24 h of RANKL stimulation. Expression levels relative to the untreated controls are shown. Each dot represents organoids from an independent BC PDX tumor. Mean ± SEM and *p*-value of two-tailed *t*-student test are shown.

© 2023 The Authors

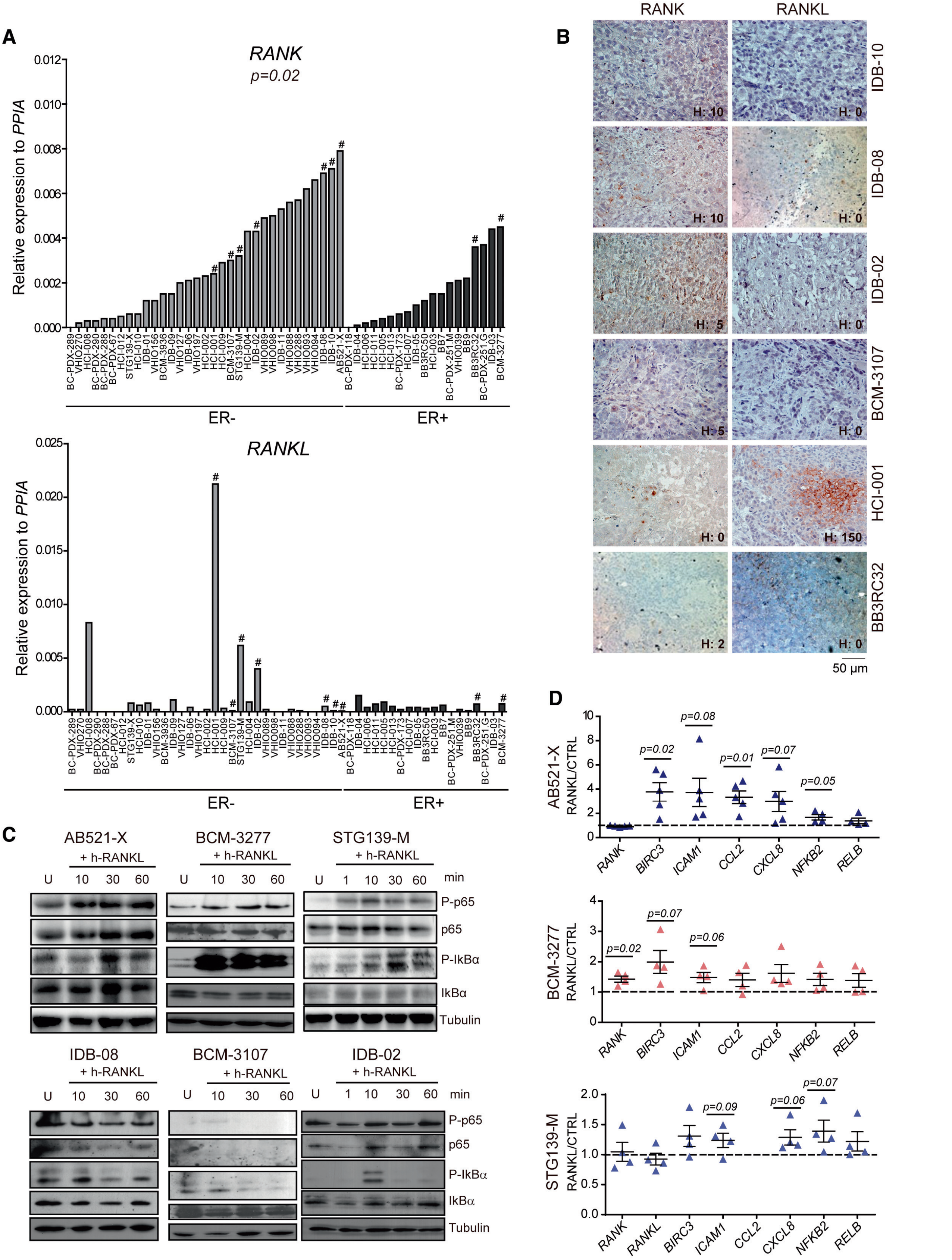
A
RANK
p=0.02
Relative expression to PPIA
ER-
ER+
RANKL
Relative expression to PPIA
ER-
ER+
B
RANK
RANKL
IDB-10
IDB-08
IDB-02
BCM-3107
HCI-001
BB3RC32
50 µm
C
AB521-X
BCM-3277
STG139-M
+ h-RANKL
U 10 30 60
min
P-p65
p65
P-IkBα
IkBα
Tubulin
IDB-08
BCM-3107
IDB-02
D
AB521-X RANKL/CTRL
BCM-3277 RANKL/CTRL
STG139-M RANKL/CTRL
RANK
RANKL
BIRC3
ICAM1
CCL2
CXCL8
NFKB2
RELB

Figure EV2.

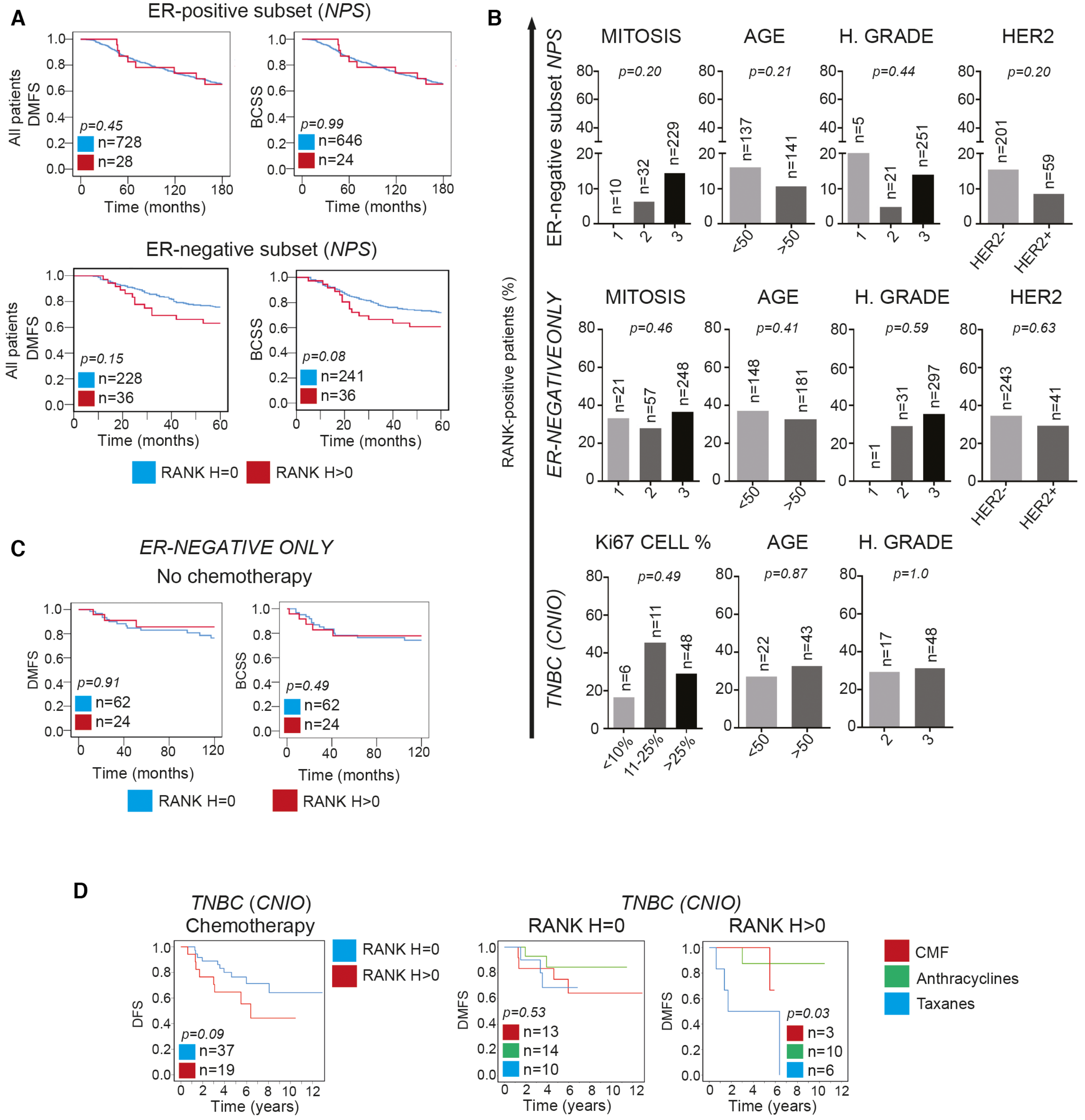

Figure EV3. RANK tumor expression is not related to any clinicopathologic factor in ER⁻ BC.

A BCSS and DMFS in the ER⁺ and ER⁻ subsets from the *NPS* collection according to RANK expression in all patients.

B Percentage of RANK⁺ BC patients according to the indicated clinicopathologic parameters in the three ER⁻ collections analyzed: *NPS* ER⁻ subset, *ER-NEGATIVE ONLY* and *TNBC (CNIO)*. Total number of analyzed patients per parameter and *p*-values (Pearson's Chi-Square test (Exact Sig. 2-side)) are indicated.

C DMFS and BCSS according to RANK expression in patients with ER⁻ tumors not treated with chemotherapy in the *ER-NEGATIVE ONLY* collection.

D RANK expression and DMFS in RANK⁻ (H = 0) and RANK⁺ (H > 0) tumor samples from the *TNBC (CNIO)* collection treated with chemotherapy and according to the chemotherapy regimen (group 1: CMF (cyclophosphamide, methotrexate, 5-fluorouracil); group 2: FAC (5-flurouracil, doxorubicin, cyclophosphamide) or FEC (5-fluorouracil, epirubicin and cyclophosphamide); and group 3: CMF or FAC or FEC plus taxanes).

Data information: (A, C, D) Total number of analyzed patients per parameter and *p*-values (Log-rank test (Mantel-Cox)) are indicated.

Figure EV4. RANKL inhibition in BC PDXs.

A Trap5b levels in mouse serum (n = 2) in tumor-bearing PDXs treated *in vivo* with RANK-Fc or DNS. Mean ± SD is shown.

B, C Representative images (B) and quantification (C) of KI67 and cleaved caspase-3 staining measured by IHC in $RANK^{+}$ tumors of PDXs treated *in vivo* with RANK-Fc or DNS. Each dot represents one picture. Three representative pictures per tumor were quantified and at least 3–4 tumors per condition were analyzed. Mean ± SEM and two-tailed t-test p-values are shown.

D Percentage of cells with ALDH activity in tumors isolated from the indicated PDXs. Each dot represents one tumor. Mean ± SEM and p-value of two-tailed t-student test are shown.

E Gene set enrichment analysis (GSEA) of associated genes after *in vivo* treatment with RANK-Fc in NSG mice, which are common for the 3 PDX models studied. The matrix illustrates NES and FDR values. The color scale represents the NES. The size of the bubbles is proportional to the -log10 of the FDR. For those signatures with an FDR = 0 after 1,000 permutations, we assigned an FDR = 10^-3 for visualization purposes. The signatures selected for this plot belong to Hallmark, Biocarta, Reactome and KEGG collections and have a reported FDR < 0.05 and a NES > 0 for all PDX models. The color legend indicates the main biological process associated with each signature.

Data information: (A–D) All the analysis were performed 24 h after last treatment.

© 2023 The Authors

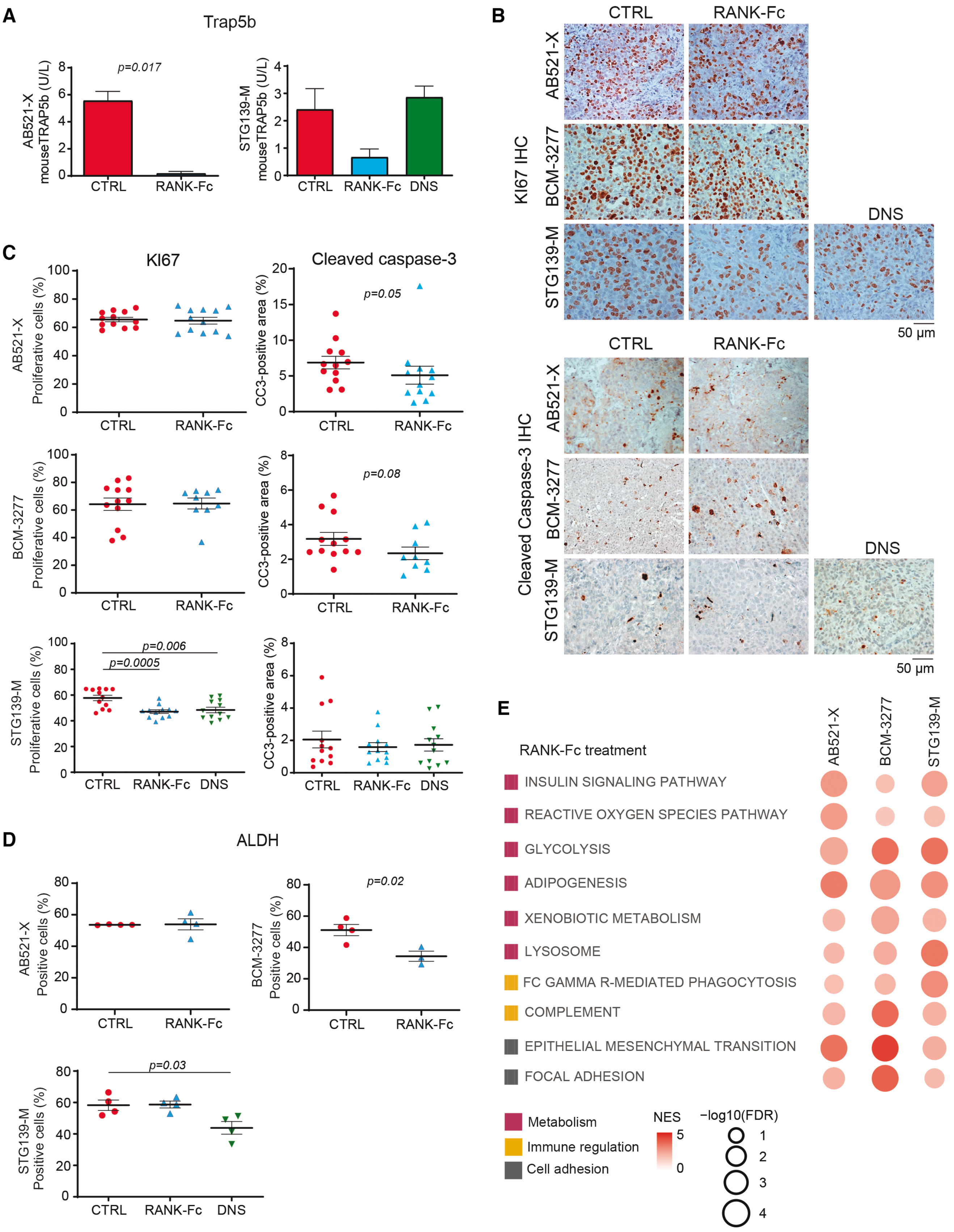
A
Trap5b
AB521-X mouseTRAP5b (U/L)
p=0.017
CTRL
RANK-Fc
STG139-M mouseTRAP5b (U/L)
CTRL
RANK-Fc
DNS
B
CTRL
RANK-Fc
DNS
KI67 IHC
AB521-X
BCM-3277
STG139-M
50 µm
Cleaved Caspase-3 IHC
C
KI67
Cleaved caspase-3
Proliferative cells (%)
CC3-positive area (%)
p=0.05
p=0.08
p=0.006
p=0.0005
D
ALDH
Positive cells (%)
p=0.02
p=0.03
E
RANK-Fc treatment
INSULIN SIGNALING PATHWAY
REACTIVE OXYGEN SPECIES PATHWAY
GLYCOLYSIS
ADIPOGENESIS
XENOBIOTIC METABOLISM
LYSOSOME
FC GAMMA R-MEDIATED PHAGOCYTOSIS
COMPLEMENT
EPITHELIAL MESENCHYMAL TRANSITION
FOCAL ADHESION
Metabolism
Immune regulation
Cell adhesion
NES
−log10(FDR)

Figure EV4.

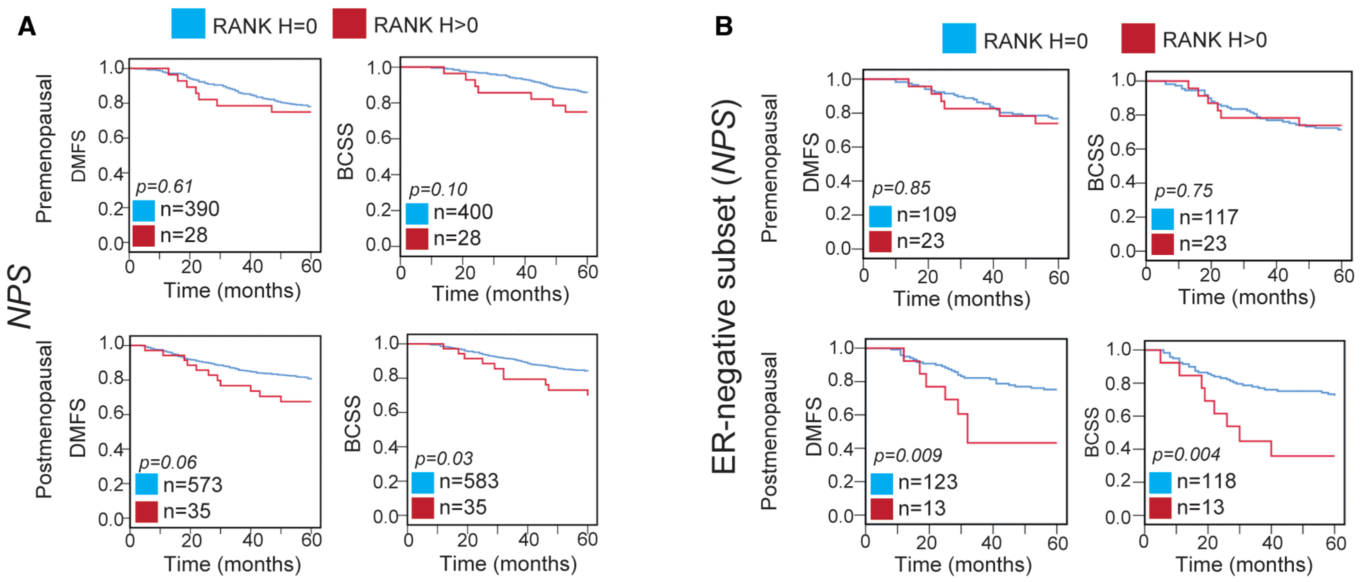

Figure EV5. RANK is a marker of poor prognosis in BC after menopause.

A, B DMFS and BCSS according to RANK expression (RANK^{-} (H = 0) or RANK^{+} (H > 0)) in premenopausal and postmenopausal patients of the *NPS* collection (A) and the *NPS* ER^{-} subset (B).

© 2023 The Authors
