# Supplementary material for: RANK is a poor prognosis marker and a therapeutic target in ER‐negative postmenopausal breast cancer
Source: EMBO Mol Med. 2023 Mar 7;15(4):e16715. doi: 10.15252/emmm.202216715 (PMC10086586; doi:10.15252/emmm.202216715)
Supplement: Supplementary file 6 — PDF+ [file EMMM-15-e16715-s004.pdf]

Report

TRANSPARENT PROCESS

OPEN ACCESS

EMBO Molecular Medicine

RANK is a poor prognosis marker and a therapeutic target in ER-negative postmenopausal breast cancer

Marina Ciscar[1,2,†], Eva M Trinidad[2,†], Gema Perez-Chacon[1], Mansour Alsaleem[3,‡], Maria Jimenez[1], Maria J Jimenez-Santos[4], Hector Perez-Montoyo[2], Adrian Sanz-Moreno[2], Andrea Vethencourt[2,5], Michael Toss[3], Anna Petit[6], Maria T Soler-Monso[6], Victor Lopez[1], Jorge Gomez-Miragaya[2], Clara Gomez-Aleza[2], Lacey E Dobrolecki[7], Michael T Lewis[7], Alejandra Bruna[8,§], Silvana Mouron[9], Miguel Quintela-Fandino[9], Fatima Al-Shahrour[4], Antonio Martinez-Aranda[2,5], Angels Sierra[2,¶], Andrew R Green[3], Emad Rakha[3] & Eva Gonzalez-Suarez[1,2,*]

Abstract

Despite strong preclinical data, the therapeutic benefit of the RANKL inhibitor, denosumab, in breast cancer patients, beyond the bone, is unclear. Aiming to select patients who may benefit from denosumab, we hereby analyzed RANK and RANKL protein expression in more than 2,000 breast tumors (777 estrogen receptor-negative, ER⁻) from four independent cohorts. RANK protein expression was more frequent in ER⁻ tumors, where it associated with poor outcome and poor response to chemotherapy. In ER⁻ breast cancer patient-derived orthoxenografts (PDXs), RANKL inhibition reduced tumor cell proliferation and stemness, regulated tumor immunity and metabolism, and improved response to chemotherapy. Intriguingly, tumor RANK protein expression associated with poor prognosis in postmenopausal breast cancer patients, activation of NFKB signaling, and modulation of immune and metabolic pathways, suggesting that RANK signaling increases after menopause. Our results demonstrate that RANK protein expression is an independent biomarker of poor prognosis in postmenopausal and ER⁻ breast cancer patients and support the therapeutic benefit of RANK pathway inhibitors, such as denosumab, in breast cancer patients with RANK⁺ ER⁻ tumors after menopause.

Keywords breast cancer patient-derived xenografts; ER negative breast cancer; menopause; pharmacological RANKL inhibitors; RANK-RANKL
Subject Categories Biomarkers; Cancer
DOI 10.15252/emmm.202216715 | Received 8 August 2022 | Revised 24 January 2023 | Accepted 8 February 2023 | Published online 7 March 2023
EMBO Mol Med (2023) 15: e16715

Introduction

Despite recent advances in treatment, breast cancer (BC) is the main cause of mortality by cancer in women, highlighting the unmet need of identifying new prognosis markers and personalized treatments. BC shows a high pathological and biological heterogeneity in histology, genetics, and sensitivity to therapies. The expression of estrogen and progesterone receptor (ER, PR), human epidermal growth factor receptor 2 (HER2) and KI67 are determinant for BC prognosis

1 Molecular Oncology, Spanish National Cancer Research Centre (CNIO), Madrid, Spain
2 Oncobell, Bellvitge Biomedical Research Institute (IDIBELL), Barcelona, Spain
3 Nottingham Breast Cancer Research Centre, Academic Unit for Translational Medical Sciences, School of Medicine, University of Nottingham Biodiscovery Institute, University Park, Nottingham, UK
4 Bioinformatics Unit, Structural Biology, Spanish National Cancer Research Centre (CNIO), Madrid, Spain
5 Medical Oncology, Breast Unit, Catalan Institute of Oncology (ICO), University Hospital of Bellvitge, Barcelona, Spain
6 Pathology Department, University Hospital of Bellvitge, IDIBELL, Barcelona, Spain
7 Molecular and Cellular Biology and Radiology, The Lester and Sue Smith Breast Center, Baylor College of Medicine, Houston, Texas, USA
8 Cancer Research UK Cambridge Centre, Cambridge, UK
9 Breast Cancer Clinical Research Unit, Clinical Research Program, Spanish National Cancer Research Centre (CNIO), Madrid, Spain
*Corresponding author. Tel: +34 917328000; E-mail: egsuarez@idibell.cat, egonzalez@cnio.es
†These authors contributed equally to this work
‡Present address: Department of Applied Medical Science, Applied College, Qassim University, Unayzah, Saudi Arabia
§Present address: Molecular Pathology Division, Centre for Paediatric Oncology Experimental Medicine, Centre for Cancer Evolution, The Institute of Cancer Research, London, UK
¶Present address: Laboratory of Experimental Oncological Neurosurgery, Neurosurgery Service, Hospital Clinic de Barcelona-FCRB, Barcelona, Spain

© 2023 The Authors. Published under the terms of the CC BY 4.0 license.

and treatment (Perou *et al*, 2000; Cheang *et al*, 2009). Tumors lacking ER, PR, and HER2 (triple negative BC, TNBC) have the worst outcome among BC subtypes in part due to limited therapeutic options (Dent *et al*, 2007).

RANKL and its receptor RANK are potential predictor biomarkers in BC. RANK is expressed on tumor cells in 40% of hormone receptor-negative tumors and 20% of the luminal tumors (Palafox *et al*, 2012). RANK expression is associated with a higher risk of relapse and death (Pfitzner *et al*, 2014). By contrast, RANKL is rarely found in tumor cells, being mostly restricted to the luminal A-like subset (Pfitzner *et al*, 2014; Azim *et al*, 2015).

Preclinical studies support RANK signaling as a therapeutic target in BC; it regulates mammary tumor initiation mediating the proliferative response to progesterone and the expansion of mammary stem cells and progenitors (Gonzalez-Suarez *et al*, 2010; Joshi *et al*, 2010; Schramek *et al*, 2010). Loss of RANK signaling prevents or attenuates mammary tumorigenesis, induces tumor cell apoptosis and/or differentiation, reduces recurrence and metastasis in $Rank^{+}$ mouse mammary tumors, and enhances tumor immunity (Gonzalez-Suarez *et al*, 2010; Schramek *et al*, 2010; Nolan *et al*, 2016; Yoldi *et al*, 2016; Gómez-Aleza *et al*, 2020).

Denosumab, a fully human monoclonal antibody against RANKL, is currently used for the treatment of osteoporosis and skeletal-related events arising from bone metastases (Miyazaki *et al*, 2014). In BC, adjuvant denosumab improved the disease-free survival (DFS) in postmenopausal women with luminal BC (ABCSG-18 trial/NCT00556374; Gnant *et al*, 2018), but in the D-CARE study/NCT01077154, no benefit in survival upon denosumab was found in any subgroup (Coleman *et al*, 2020). These conflicting results highlight the need of further knowledge in the understanding of RANK biology in BC and its therapeutic potential.

In this study, we have evaluated the potential value of RANK and RANKL protein expression as clinical predictors of BC prognosis and the therapeutic value of targeting RANK signaling in human BC.

Results

RANK expression in tumor cells associates with ER/PR-negative tumors

We analyzed the expression of RANK and RANKL proteins in two independent tissue-microarray (TMA) collections containing all BC subtypes: *IDIBELL* (*IDB*) (n = 404; Martínez-Aranda *et al*, 2015) and *Nottingham Primary Series* (*NPS*) (n = 1,895 samples, 298 included in the Molecular Taxonomy of Breast Cancer International Consortium (*METABRIC*); Curtis *et al*, 2012; Green *et al*, 2013). RANK protein was detected in the tumor compartment in 18.3 and 5.7% of samples from *IDB* and *NPS*, respectively, as well as in the stroma of half of the samples. Tumor expression of transmembrane RANKL (tmRANKL) was found in only 4.6% (*IDB*) and 3.5% (*NPS*) of adenocarcinomas and rarely observed in the stroma (< 3%; Fig 1A and B). Similar expression patterns for RANK and RANKL were found in the *METABRIC* subset (Fig EV1A). Fig EV1B shows the H-Score (H) for tumor RANK and tmRANKL in the three collections.

RANK was more frequently found in the ER^{-} compared with the ER^{+} subsets of *IDB* (40.4% vs. 21.3%) and *NPS* (13% vs. 3.7%) samples (Fig 1C). In line with previous results (Gonzalez-Suarez *et al*, 2010; Pfitzner *et al*, 2014), RANK expression (H > 0) was associated with ER/PR negativity and TNBC subtype, but not with HER2, age, tumor size or grade in all cohorts (Figs 1C and EV1C). In the *NPS*, RANK expression was also associated with a higher mitosis rate and grade (Fig EV1C; Dataset EV1). The low frequency of $tmRANKL^{+}$ samples prevented associations with clinicopathologic parameters.

Patients with $RANK^{+}$ tumors tended to have a poorer distant metastasis-free survival (DMFS) (*IDB* and *NPS*) and BC-specific survival (BCSS) (*NPS*) compared with those with $RANK^{-}$ tumors (Fig 1D; Dataset EV1). Moreover, RANK expression (*NPS*) was associated with shorter BCSS, independent of ER, tumor grade, stage and size (Fig 1E; Dataset EV1). Altogether, our results show that RANK protein expression associates with ER^{-}/PR^{-} tumors and poor outcome.

RANK is expressed in ER^{-} BC patient-derived orthoxenografts (PDXs) and it is responsive to RANKL

Despite encouraging results in BC mouse models and cell lines, RANK functional relevance in clinical BC remains poorly studied. Thus, we analyzed *RANK* and *RANKL* gene and protein expression in 76 PDXs from several BC collections (Derose *et al*, 2011; Zhang & Lewis, 2013; Bruna *et al*, 2016; Eyre *et al*, 2016; Gómez-Miragaya *et al*, 2017; Gris-Oliver *et al*, 2020). *RANK* mRNA levels were higher in PDXs derived from ER^{-} tumors than ER^{+}, while *RANKL* was low or undetectable in most PDXs, with some exceptions (Fig EV2A). RANK protein was found in 40 and 14.3% of those PDX derived from ER^{-} and ER^{+} BC, respectively, whereas tmRANKL was only detected in few models (Figs 1F and G, and EV2B; Dataset EV2), recapitulating clinical patterns (Fig 1A and C). Enhanced phosphorylation of IkBα and/or p65 and upregulation of RANK/NFKB targets after RANKL treatment confirmed activation of RANK signaling in AB521-X, BCM-3277 and STG139-M, but not in other $RANK^{+}$ PDXs (Fig EV2C and D). The AB521-X and STG139-M models, derived

Figure 1. Tumor RANK is expressed and active in human BC and it associates with ER^{-} BC subtype and poor survival.

A Percentage of patients expressing tumor and stromal RANK or tmRANKL (H > 0) in BC samples.
B Representative images showing RANK and tmRANKL protein expression in tumor and stromal cells in human BC determined by IHC.
C Percentage of BC patients with $RANK^{+}$ tumors according to ER expression, p-values (Pearson's ChiSquare test (Exact Sig. 2-Side)).
D DMFS and BCSS according to RANK expression. p-values (Log-rank test (Mantel-Cox)).
E Forest plots showing HR, 95% CI and p-values from uni or multivariate regression analyses for the indicated survival parameters.
F Percentage of PDXs expressing RANK protein according to ER expression. p-value (Two tailed t-test).
G Representative images of RANK and RANKL protein expression in BC PDXs. H-Score (H) of each PDX model is indicated.
H Venn diagram (left) shows the pathways (FDR < 0.25) modulated by RANKL in each PDX and those shared. Pie chart (right) represents the percentage of pathways involved in cited biological processes.

Data information: (A, C, D, F) The total number of patients or PDX is shown.

 © 2023 The Authors

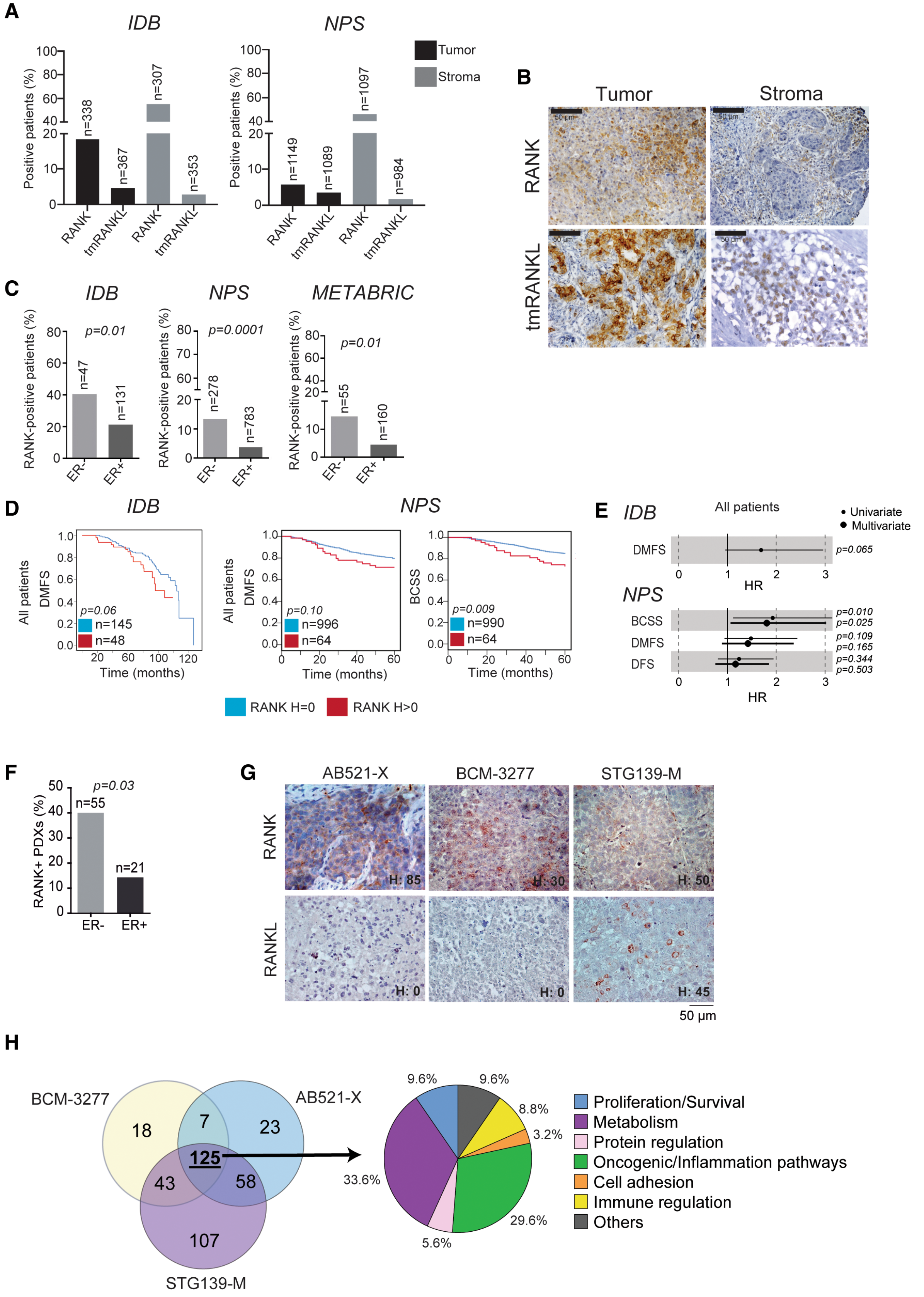
A
IDB
NPS
Tumor
Stroma
Positive patients (%)
n=338
n=367
n=307
n=353
n=1149
n=1089
n=1097
n=984
RANK
tmRANKL
B
Tumor
Stroma
RANK
tmRANKL
C
IDB
NPS
METABRIC
RANK-positive patients (%)
p=0.01
p=0.0001
p=0.01
n=47
n=131
n=278
n=783
n=55
n=160
ER-
ER+
D
IDB
NPS
All patients
DMFS
BCSS
p=0.06
n=145
n=48
p=0.10
n=996
n=64
p=0.009
n=990
n=64
Time (months)
RANK H=0
RANK H>0
E
IDB
All patients
Univariate
Multivariate
DMFS
p=0.065
HR
NPS
BCSS
DMFS
DFS
p=0.010
p=0.025
p=0.109
p=0.165
p=0.344
p=0.503
F
p=0.03
n=55
n=21
RANK+ PDXs (%)
ER-
ER+
G
AB521-X
BCM-3277
STG139-M
RANK
RANKL
H: 85
H: 30
H: 50
H: 0
H: 0
H: 45
50 µm
H
BCM-3277
AB521-X
STG139-M
18
7
23
125
43
58
107
9.6%
9.6%
8.8%
3.2%
33.6%
29.6%
5.6%
Proliferation/Survival
Metabolism
Protein regulation
Oncogenic/Inflammation pathways
Cell adhesion
Immune regulation
Others

Figure 1.

from ER^- BCs, and the BCM-3277 model, derived from ER^+ BC, but ER^- in the PDX, were selected for *in vivo* experiments. Upon RANKL exposure *in vivo*, more than 200 pathways were regulated (FDR < 0.25) in each PDX and most (> 100) were shared between the three PDXs (Fig 1H; Dataset EV3, Table B, F, J, O). The top-ranked RANKL-driven common pathways (NES > 0) were related to TNF/NFKB signaling (confirming RANK activation), metabolism, oncogenic/inflammation, immunity, and proliferation (Fig 1H; Dataset EV3, Table O). These results demonstrate that RANK/RANKL expression patterns in BC PDXs recapitulate clinical findings and that activation of RANK signaling has pleiotropic effects in human breast adenocarcinomas.

ER expression determines RANK biology and prognosis value in BC

Given the differences in prognosis between ER^+ and ER^- BC and the increased $RANK^+$ in ER^- BC, we assessed the significance of tumor $RANK^+$ separately in both subsets. RANK positivity did not associate with survival in the *NPS* ER^+ patients, but in the *NPS* ER^- subset, there was a trend toward worse DMFS and BCSS. RANK did not associate with any of the clinicopathologic factors analyzed (Dataset EV1; Fig EV3A and B). Although no associations between $RANK^+$ and $HER2^+$ were found, RANK expression associated with poor survival in $HER2^+$ tumors, but the small sample size prevented solid conclusions (Dataset EV1).

Thanks to the availability of gene expression data from the *METABRIC* dataset, we identified the pathways differentially regulated in $RANK^+$ tumors: 67 in ER^+ and 17 in ER^- BC patients, with no overlap between them (FDR < 0.25). In ER^+ BC, RANK associated with replication/transcription, while in ER^- BC, RANK seemed to modulate multiple metabolic processes (NES < 0) (Fig 2A; Dataset EV4). Several of these pathways were regulated upon RANKL treatment in the ER^- PDX models, indicating direct regulation by RANK signaling (Dataset EV3, Venn in Dataset EV4). Altogether, these results highlight the different biology of RANK signaling according to ER status, which may contribute to the differences in prognosis observed between $RANK^+$ ER^+ and ER^- BC.

RANK expression in ER^- tumors associates with poor survival and response to chemotherapy

As the frequency of RANK positivity in the *NPS* collection was low (13%), we analyzed two additional and more recent collections of ER^- tumors: the *ER-NEGATIVE ONLY* collection (n = 359 ER^- tumors) and the *TNBC (CNIO)* collection (n = 66). Tumor $RANK^+$ was found in 34 and 30.3% of the samples, respectively (Fig 2B), in line with previous reports (Palafox *et al*, 2012; Pfitzner *et al*, 2014). Again, while tumor RANK expression was not associated with any of the clinicopathologic factors analyzed (Fig EV3B; Dataset EV1), in the *ER-NEGATIVE ONLY* collection, patients with $RANK^+$ tumors showed a significant poorer 10-year survival compared with patients with $RANK^-$ tumors (Fig 2C), confirming the results of the *NPS* ER^- subset (Fig EV3A). Indeed, RANK expression was an independent factor of worse DMFS and DFS in ER^- patients (*ER-NEGATIVE ONLY*; Fig 2D; COX Dataset EV1). When HER2 expression, BRCA1 mutations and basal markers were considered, sample size was too small to get solid conclusions, although association of $RANK^+$ with poor DFS in $HER2^+$ ER^- tumors was observed (Dataset EV1).

Patients with ER^- $RANK^+$ tumors showed poorer survival after adjuvant chemotherapy (mainly taxanes and anthracyclines) than those lacking RANK (Fig 2D), while no survival differences associated with RANK were found in the absence of chemotherapy (Figs 2E and EV3C; Dataset EV1). Similarly, in the *TNBC (CNIO)* collection tumors expressing RANK tended to have worse survival in patients receiving chemotherapy, particularly to regimens containing taxanes (Fig EV3D; Dataset EV1). Altogether, these results point out the importance of RANK expression as an independent biomarker of both poor prognosis and chemotherapy response in ER^- BC.

RANKL inhibition improved response to docetaxel in ER^- $RANK^+$ BC PDXs

Our clinical results prompted us to evaluate whether RANKL therapeutic inhibition *in vivo* would impact the growth of the ER^- $RANK^+$ BC PDX. Tumor-bearing mice were randomized for treatment with the RANKL inhibitors RANK-Fc or denosumab (the latter used only in the STG139-M model as it expresses hRANKL) or mock (control). Serum levels of the bone remodeling marker, Trap5b, decreased upon RANK-Fc, confirming the efficacy of the treatment, but not after denosumab as it only binds to human RANKL (Fig EV4A). RANK-Fc and denosumab decreased tumor cell proliferation and attenuated tumor growth in the STG139-M model, suggesting that the anti-proliferative effects were mainly due to inhibition of tumor RANKL (Figs 2F and EV4B and C), in line with (Gonzalez-Suarez *et al*, 2010). Tumor apoptosis was comparable between groups (Fig EV4B and C) and RANKL inhibition reduced ALDH activity in BCM-3277 and STG139-M (Fig EV4D), supporting a reduction in stemness (Yoldi *et al*, 2016).

Figure 2. RANK tumor expression associates with poor survival in ER^- BC and RANKL inhibition improves response to chemotherapy.

A Bubble matrix represents GSEA results of pathways associated with RANK protein expression in the *METABRIC* collection classified by ER expression. The matrix illustrates the NES and FDR values (empty bubbles FDR > 0.25). Color legend indicates the main biological process associated.

B Percentage of $RANK^+$ tumors in indicated ER^- collections.

C DMFS and BCSS according to RANK expression.

D Forest plots showing HR, 95% CI and uni or multivariate *p*-values for the indicated survival parameters.

E DMFS and BCSS after chemotherapy (anthracyclines/taxanes) according to RANK expression.

F, G Tumor growth curves (($\pi \times$ length $\times$ width^2)/6) of the indicated PDXs after treatment with RANK-Fc, denosumab (DNS) or mock (CTRL), alone (F) or in combination with docetaxel (DTX) (G). (G) Bottom left shows tumor growth/regression during DTX/RANKL-inhibitor treatment for STG139-M. Bottom right shows the tumor relapse in these same mice after removal of the treatment. Each thin curve represents one single tumor, and each thick curve represents the mean of all tumors implanted. Linear regression analysis and two-tailed *p*-value are shown.

Data information: (B, C, E) Total number of analyzed patients per parameter and *p*-values (Log-rank test (Mantel-Cox)) are indicated.

© 2023 The Authors

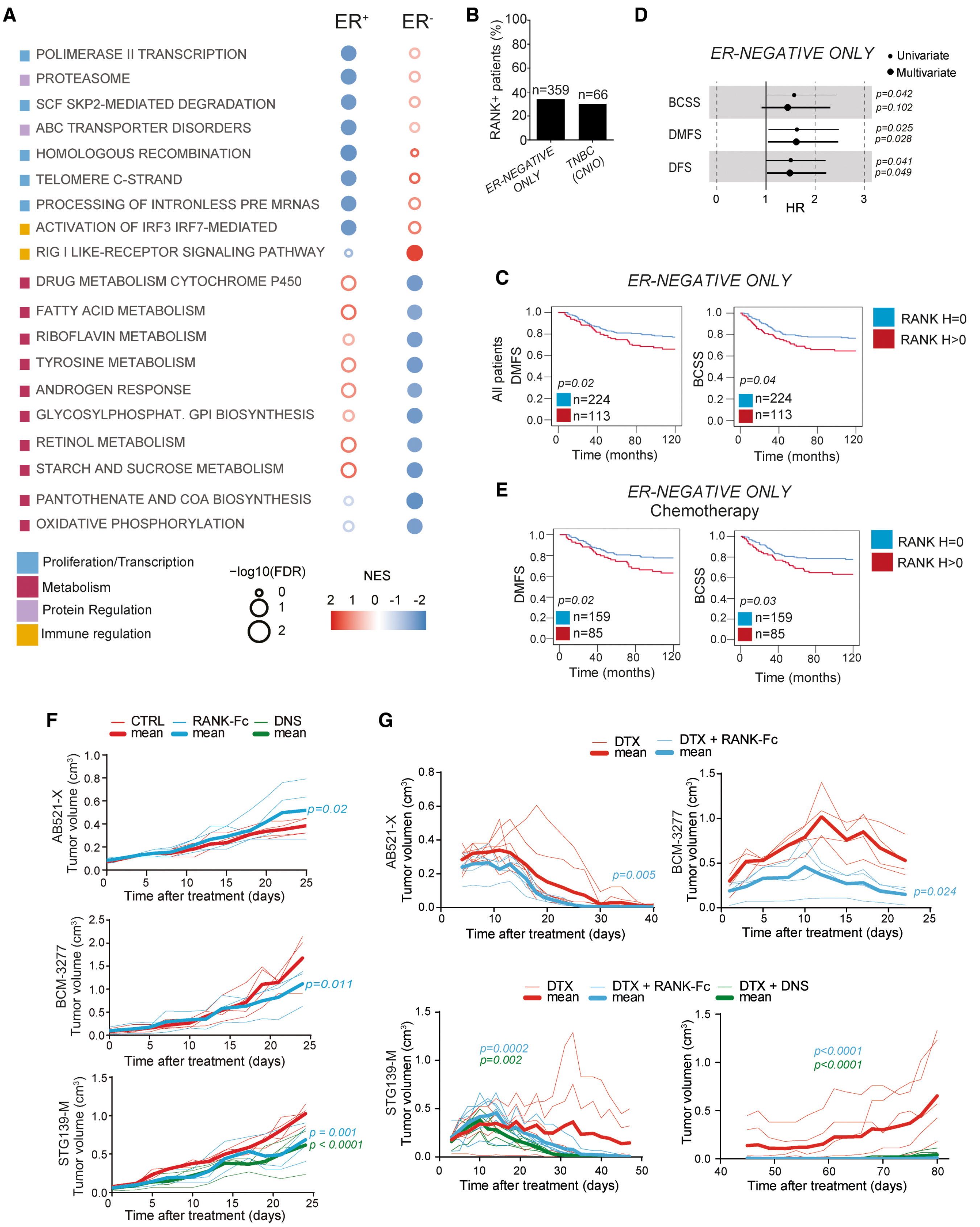

A
ER+
ER-
POLIMERASE II TRANSCRIPTION
PROTEASOME
SCF SKP2-MEDIATED DEGRADATION
ABC TRANSPORTER DISORDERS
HOMOLOGOUS RECOMBINATION
TELOMERE C-STRAND
PROCESSING OF INTRONLESS PRE MRNAS
ACTIVATION OF IRF3 IRF7-MEDIATED
RIG I LIKE-RECEPTOR SIGNALING PATHWAY
DRUG METABOLISM CYTOCHROME P450
FATTY ACID METABOLISM
RIBOFLAVIN METABOLISM
TYROSINE METABOLISM
ANDROGEN RESPONSE
GLYCOSYLPHOSPHAT. GPI BIOSYNTHESIS
RETINOL METABOLISM
STARCH AND SUCROSE METABOLISM
PANTOTHENATE AND COA BIOSYNTHESIS
OXIDATIVE PHOSPHORYLATION
Proliferation/Transcription
Metabolism
Protein Regulation
Immune regulation
−log10(FDR)
NES
B
RANK+ patients (%)
n=359
n=66
ER-NEGATIVE ONLY
TNBC (CNIO)
D
ER-NEGATIVE ONLY
Univariate
Multivariate
BCSS
DMFS
DFS
p=0.042
p=0.102
p=0.025
p=0.028
p=0.041
p=0.049
HR
C
ER-NEGATIVE ONLY
All patients
DMFS
BCSS
p=0.02
p=0.04
n=224
n=113
Time (months)
RANK H=0
RANK H>0
E
ER-NEGATIVE ONLY
Chemotherapy
p=0.02
p=0.03
n=159
n=85
F
CTRL mean
RANK-Fc mean
DNS mean
AB521-X
Tumor volume (cm³)
p=0.02
BCM-3277
p=0.011
STG139-M
p = 0.001
p < 0.0001
Time after treatment (days)
G
DTX mean
DTX + RANK-Fc mean
DTX + DNS mean
Tumor volumen (cm³)
p=0.005
p=0.024
p=0.0002
p=0.002
p<0.0001
p<0.0001

Figure 2.

Transcriptomic analyses upon RANKL inhibition revealed changes in tumor metabolism, immunity, adhesion, among others, with > 100 pathways (FDR < 0.25) shared between the three PDXs (Fig EV4E; Dataset EV3, Table P). Denosumab behaved as RANK-Fc in STG139-M (Venn in Dataset EV3, Table Q). The genetic signature obtained in DNS-treated patients from the D-BEYOND clinical trial (NCT01864798; Gómez-Aleza *et al*, 2020), was associated with RANKL inhibition in the three $RANK^+$ PDXs (Dataset EV3, Table R). Moreover, pathways such as fatty acid metabolism and oxidative phosphorylation were shared between the three RANK-Fc-treated PDXs and those associated with RANK expression in the ER^- *METABRIC* samples (Venn in Dataset EV4). This reinforces the clinical relevance of the PDX BC models and the pleiotropic effect of RANKL inhibitors in BC. Finally, in line with the association between tumor RANK expression and the poor response to chemotherapy observed in clinical samples, increased benefit was observed when RANKL inhibitors were combined with docetaxel in the three PDXs (Fig 2G). In the STG139-M model, the combination led to complete tumor regression and no tumor relapse after interruption of docetaxel (Fig 2G). These results demonstrate that inhibition of RANK signaling improves response to chemotherapy in ER^- BC.

RANK expression associates with poor prognosis in postmenopausal BC

Finally, we evaluated whether the prognostic value of RANK expression in BC would change with menopause, as RANK pathway is regulated by sex hormones. Interestingly, $RANK^+$ associated with poor survival in postmenopausal, but not in premenopausal patients from the general *IDB* (DMFS) and *NPS* (BCSS) collections (Figs 3A and EV5A; Dataset EV1). Multivariate analyses showed that $RANK^+$ associated with worse BCSS and DMFS only in postmenopausal women (*NPS*) (Dataset EV1, COX *NPS*). These findings were also validated in ER^- patients. RANK expression was an independent factor of worse DMFS and DFS in postmenopausal, but not in premenopausal patients in tumors from *ER-NEGATIVE ONLY* collection (Fig 3B and C; Dataset EV1). Even in the *NPS* ER^- subset, $RANK^+$ associated with worse DMFS and BCSS in postmenopausal patients (Fig EV5B; Dataset EV1). Results from three independent collections demonstrated that RANK expression is an independent biomarker of poor prognosis in postmenopausal BC.

GSEA revealed that tumor RANK expression in postmenopausal cases was positively associated (FDR < 0.25) with 20 pathways, 12 of them related to TNF/NFKB signaling, including RANKL pathway itself, while in premenopausal tumors, only three pathways were associated with $RANK^+$ (Fig 3D; Dataset EV4), suggesting that, similar to the bone, RANK signaling in BC is more active after menopause (Streicher *et al*, 2017). Meanwhile, in postmenopausal ER^- tumors, $RANK^+$ was positively associated with NFKB activation and immunity. Negative associations with multiple metabolic pathways (insulin/IGF1 signaling, fatty acid metabolism, mTOR, cholesterol homeostasis and oxidative phosphorylation) were found (Fig 3D; Dataset EV4). Again, these same pathways were modulated by RANK signaling in the PDX (Fig EV4E; Venn in Dataset EV4). These findings suggest that RANK activation in BC increases after menopause, regulates tumor cell metabolism and hence, contributes to the association of RANK expression with poor prognosis in ER^- postmenopausal BC.

Discussion

Great heterogeneity persists in all BC subtypes that translates into wide-range of responses to current, and still limited, treatments. Searching for new prognostic and predictive factors has become an essential task for the individualization of BC therapy (Weigel & Dowsett, 2010).

In this work, the analyses of RANK and RANKL in more than 2,000 BC samples from four independent TMA cohorts, confirmed that RANK expression was associated with ER^- tumors while RANKL was rarely found in tumor cells (Palafox *et al*, 2012; Pfitzner *et al*, 2014). The large number of samples analyzed in our study allowed to define RANK expression as an independent poor prognosis factor in BC, in particular in ER^- BC and in postmenopausal women. The distinct biology associated with RANK signaling according to ER status may explain why RANK predicts poor prognosis in ER^-, but not in ER^+ BC. RANK protein expression in ER^+ tumors was negatively associated with proliferation, in line with its association with senescence in luminal tumors (Benítez *et al*, 2021). However, additional ER^+ collections need to be evaluated to determine the prognostic value of RANK in ER^+ BC, as the low frequency of RANK positivity in the *NPS* collection is a limitation.

Transcriptomic analyses in ER^- tumors and PDXs upon modulation of RANK signaling evidence its pleiotropic role in BC, regulating multiple biological processes with a key role in tumor metabolism and immunity (Gonzalez-Suarez *et al*, 2010; Yoldi *et al*, 2016; Rao *et al*, 2017; Gómez-Aleza *et al*, 2020). Despite BC heterogeneity, a strong overlap of RANK-driven pathways was found between the different PDXs and clinical samples, which may help define a signature to select BC patients who may benefit from denosumab and the evaluation of drug response during treatment.

Our results suggest that $RANK^+$ ER^- tumors showed a worse response to chemotherapy regimens that include taxanes. Accordingly, increased therapeutic benefit was observed in the ER^- PDXs when RANKL inhibitors were combined with chemotherapy. Results from the GeparX clinical trial demonstrated that neoadjuvant denosumab in combination with nab-paclitaxel did not increase the pathological complete response in patients with early BC, not even in patients with $RANK^+$ early BC, but survival remains to be evaluated (Blohmer *et al*, 2022).

Paradoxically to the well-characterized role of RANK signaling as a mediator of progesterone in healthy breast or preneoplasic lesions (Gonzalez-Suarez *et al*, 2010; Schramek *et al*, 2010), our results demonstrate that RANK predicts poor prognosis in ER^- postmenopausal BC. The drop of estrogen levels leads to increased RANK signaling in the bone and osteoporosis (Streicher *et al*, 2017). Similarly, RANK signaling seems to increase after menopause in breast tumors, as multiple pathways related to NFKB activation, including RANKL pathway, were positively associated with RANK protein expression only in postmenopausal patients. These results suggest that denosumab would show the highest therapeutic benefit in postmenopausal women with ER^- $RANK^+$ breast tumors. The meta-analysis by the Early Breast Cancer Clinical Trialists' Collaborative Group supports the idea that adjuvant treatment of early BC might be more efficacious with the addition of a bone-modifying agent, particularly in postmenopausal women, or in combination with ovarian function suppression (Chukir *et al*, 2019; Perrone & Gravina, 2020). The increased bone remodeling and the metabolic

© 2023 The Authors

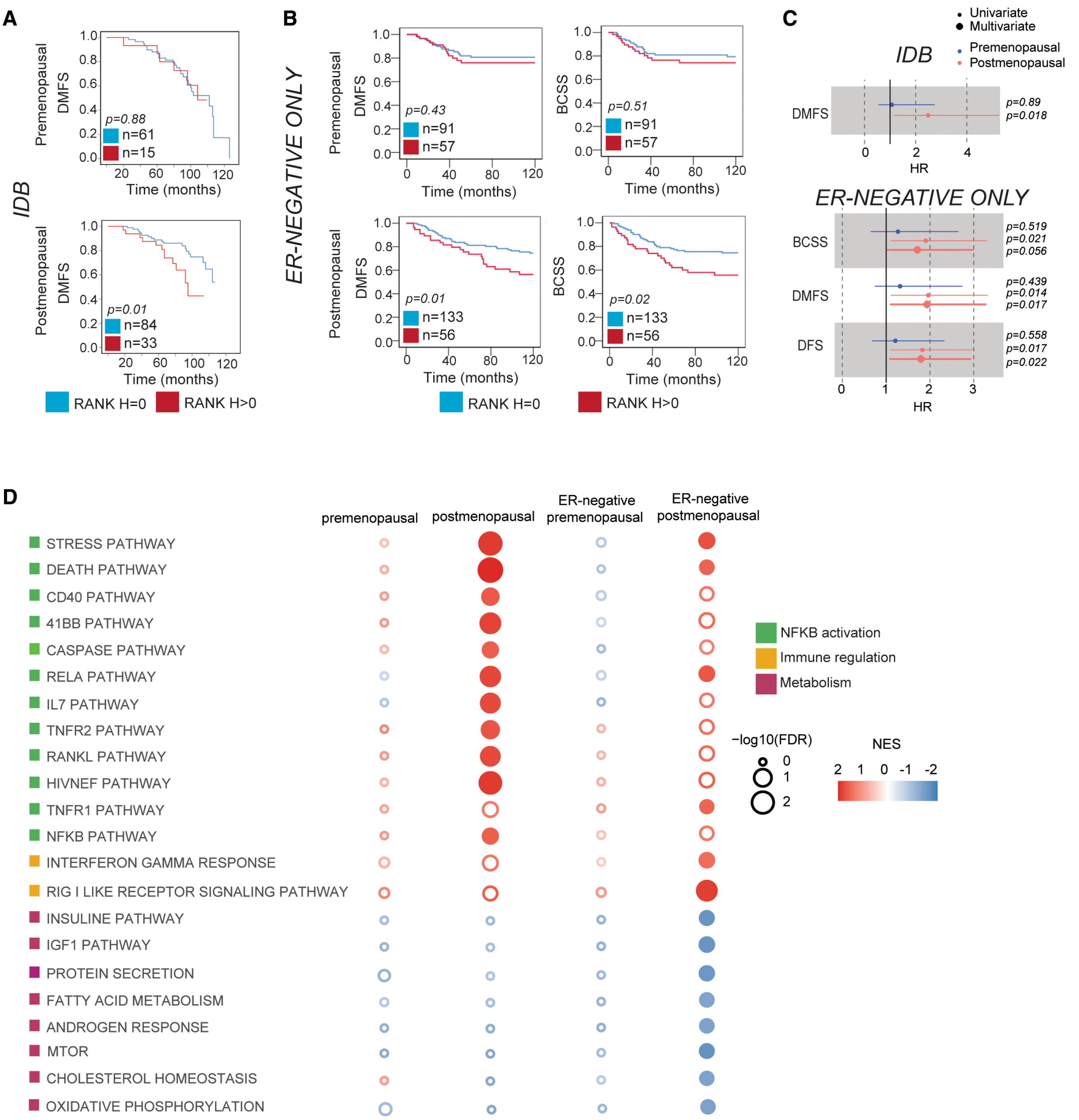

Figure 3. Tumor RANK expression associates with poor survival in postmenopausal patients and RANK biology in BC changes with menopause.

A, B DMFS and BCSS according to RANK expression in premenopausal and postmenopausal patients. Total number of analyzed patients per parameter and *p*-values (Log-rank test (Mantel-Cox)) are indicated.

C Forest plots showing HR, 95% CI and uni or multivariate *p*-values for the indicated survival parameters.

D Bubble matrix represents GSEA results of pathways associated with RANK protein expression in all patients and in the ER$^-$ subset from the *METABRIC* collection classified by menopausal status. The matrix illustrates the NES and FDR values (Empty bubbles = FDR > 0.25). Color legend indicates the main biological process associated.

changes (systemic and in the tumor) that follow the drop in estradiol levels (Khosla *et al*, 2012) may also contribute to the role of RANK as a marker of poor survival after menopause.

Results from the ABCSG18 trial revealed that adjuvant denosumab reduced the risk of bone fractures and improved DFS of ER^+ postmenopausal BC patients (Gnant *et al*, 2018), but this was not validated in the D-CARE trial (Coleman *et al*, 2020). However, in these trials, RANK expression or RANK pathway activation was not considered. Retrospective analyses of RANK pathway expression/activation are required for evaluating BC outcome after denosumab treatment.

In summary, we demonstrate that RANK is an independent marker of poor prognosis in ER^- BC after menopause and our functional analyses support the therapeutic potential of RANK pathway inhibitors in ER^- postmenopausal BC.

Materials and Methods

Tissue microarray (TMA) staining and scoring

RANK and tmRANKL expression were evaluated in TMAs from five different cohorts of BC patients. *IDB* TMA (donated by A. Sierra (IDIBELL, Spain)), contains 404 BC samples and clinicopathologic information from 314 patients (24–88 years old) diagnosed between 1989 and 2009. Follow-up ranged from 8 to 146 months (mean: 76.6 months). Metastasis relapse occurred in 43.4% (138 of 318) of patients; of these, 84 patients (60.9%) developed brain metastasis, 47 (34.1%) lung metastasis, 54 (39.1%) liver metastasis, 40 (29.0%) nonregional lymph node metastasis and 89 (64.5%) bone metastasis. Just over half (56.6%; 180 of 318) of the patients had no metastatic progression after a minimum follow-up of 5 years. *NPS* TMA is a well-characterized cohort of unselected early-stage (I–III) primary operable invasive BC from patients aged 70 years or younger, enrolled into the Nottingham Tenovus Primary Breast Carcinoma Series between 1990 and 1997 (n = 1,895), and managed in accordance with a uniform protocol; a subset of cases (n = 298) were included in the *METABRIC* study (Curtis *et al*, 2012), where gene expression data is available. Outcome data include survival status, survival time, cause of death, development, and time to locoregional recurrence and distant metastasis (DM). BCSS is defined as the time (in months) from the date of primary surgery to the date of breast cancer-related death. DMFS is defined as the time (in months) from the date of primary surgery to the appearance of DM. Treatments include chemotherapy (cyclophosphamide, methotrexate and fluorouracil (CMF)) or endocrine therapy. At that time patients with $HER2^+$ tumors had no access to trastuzumab. Positive ER status was defined as > 1% of tumor cells expressing ER. Positive HER2 status was defined using immunohistochemistry as HER2 3+. Histological grade was assessed based on the Nottingham Grading System (Elston & Ellis, 1991; Rakha *et al*, 2008). Other clinicopathologic factors such as ER, PR and/or HER2 expression, proliferation rate (KI67 expression or mitosis), vascular invasion, as well as patient age and survival analysis were analyzed before including the samples into the TMAs (Abd El-Rehim *et al*, 2005). Two additional collections of ER^- tumors were analyzed, the Nottingham *ER-NEGATIVE ONLY* cohort (1998–2006), which contains 396 samples, and the *TNBC (CNIO)*, a small collection of 66 TNBC patients with 40–50% of relapse, generated by Dr M. Quintela-Fandino (CNIO, Spain). In the *ER-NEGATIVE ONLY* cohort the chemotherapy regimen used was CMF and after year 2000 anthracyclines plus taxanes. In the *TNBC (CNIO)* cohort, three regimens were used: group 1 CMF (cyclophosphamide, methotrexate, and 5-fluorouracil), group 2 FAC (5-fluorouracil, doxorubicin, and cyclophosphamide) or FEC (5-fluorouracil, epirubicin, and cyclophosphamide), and group 3 CMF or FAC or FEC plus taxanes.

RANK or tmRANKL staining was scored for intensity (on a scale of 0 to 3; 0 = no staining, 1 = weak, 2 = moderate, 3 = intense) and positive cell percentage (on a scale of 0 to 100%) within tumor cells or surrounding stroma for each TMA core sample. The H-Score value is defined as the sum of multiplying staining intensity by positive area, ranging from 0 to 300. TMA cores with less than 30% of the core area were discarded. Patients were stratified according to RANK or tmRANKL H-Scores as being protein-positive (H > 0) or protein-negative (H = 0). As TMA samples are enriched in tumor cells, the stroma content was not always present or representative and H-Score was not calculated. Total number of scorable samples for each of the collections and stainings are indicated in the corresponding fig.

PDX models

Generation of the IDB PDXs is described by Gómez-Miragaya *et al* (2017). Briefly, they were generated by orthotopic transplantation of human fresh tumor tissue or injection of metastatic cancer cells isolated from pleural effusions into the cleared mammary fat pad of immunodeficient female mice (NOD.Cg-Prkdcscid Il2rgtm1Wjl/SzJ, RRID:IMSR_JAX:005557, The Jackson Laboratory). The rest of the PDXs were obtained through collaboration with Dr V. Serra and Dr J. Arribas (Vall d'Hebron Institute of Oncology), Dr A. Welm (Huntsman Cancer Institute), Dr M. T. Lewis (Baylor College of Medicine), Dr A. Bruna and Dr C. Caldas (Cancer Research UK Cambridge Institute) and Dr R. Clarke (Manchester Breast Centre).

PDXs were maintained by consecutive rounds of transplantation of tumor pieces. In the case of the BCM-3277 model, mice were treated with 17β-estradiol at 8 μg/ml (Sigma) in drinking water. Mice were kept in individually ventilated and open cages and food and water were provided *ad libitum*. Cages, bedding, food and water were all autoclaved. Euthanasia was performed by CO_2 inhalation. All animal experiments were conducted according to institutional policies and national and European guidelines.

Tumor cell isolation

Single cells were isolated from tumors as described previously (Smalley, 2010). Briefly, fresh tissues were mechanically dissected with a McIlwain tissue chopper and enzymatically digested with appropriate medium (DMEM F-12, 0.3% collagenase A, 2.5 U/ml dispase, 20 mM HEPES, and 100 U/ml penicillin/100 μg/ml streptomycin) 60 min at 37°C. Samples were washed with Leibowitz L15 medium/10% FBS between each step. Erythrocytes were eliminated with hypotonic lysis buffer, and fibroblasts were excluded by incubation with DMEM F-12/10% FBS 1 h at 37°C. Single epithelial cells were isolated by treating with trypsin 2 min at 37°C. The cell suspension was filtered with 40 mm cell strainers and counted.

 © 2023 The Authors

RANKL, RANK-Fc, denosumab and docetaxel treatments *in vivo*

Dissociated tumor cells mixed 1:1 with Matrigel Basement Membrane (BD Biosciences) were transplanted orthotopically in the inguinal mammary gland of 10–/12-week-old NSG mice and when tumors reached 5 mm of diameter mice were randomized for mock, h-RANKL (0.75 mg/kg, 4–6 doses, twice per week; Amgen Inc.), h-RANK-Fc treatment (10 mg/kg, three times per week; Amgen Inc.) or denosumab (10 mg/kg, three times per week; XGEVA®). Docetaxel (Hospira/Actavis, 20 mg/kg) was administered once per week together with dexamethasone (0.132 mg/kg, Merck), to reduce the chemotherapy-induced inflammation. All the drugs were administered intraperitoneally. Tumor growth was monitored and measured with a caliper once per week. Tumor volume was then calculated as follows: $\pi \times$ length $\times$ width^2/6 in cm^3. Treatment was interrupted when tumors regress below 3 mm of diameter. Mice were sacrificed and tumors were surgically removed 24 h after treatment completion. In combination treatments with docetaxel, mice were sacrificed once relapsing tumors reached 10 mm of diameter.

Flow cytometry

Single tumor cells were resuspended and incubated in blocking solution (PBS containing 2% FBS, 2 mM EDTA and IgG blocking reagent (Sigma)) for 10 min on ice. Mouse cells were excluded in flow cytometry using H2Kd-PECy7 (SF1-1.1, 116622 from BioLegend). Gating was based on "Fluorescence Minus One" controls. ALDH activity of tumor cells was assessed using the ALDEFLUOR™ Kit (01700 from STEMCELL Technologies), following the manufacturer's protocol. Live/dead staining was performed using DAPI (Thermo Fisher Scientific). A population of 10,000 alive cells was acquired in all experiments. Samples were analyzed using a Gallios flow cytometer (Beckman Coulter) and the FlowJo software.

Enzyme-linked immunosorbent assay (ELISA)

Trap5b activity was measured in mouse serum according to the manufacturer's instructions (IDS).

Tissue histology and immunostaining

Three micrometer sections was cut and immunohistochemistry of hRANK and hRANKL was performed as described (Gonzalez-Suarez *et al*, 2010). RANK antigen retrieval was carried out with the Diva Decloaker buffer (Biocare Medical), 90°C, 14–16 h; sodium citrate buffer (0.01 M, pH = 6) was used for RANKL. Protein blocking was done with TNB Blocking Buffer (PerkinElmer). Anti-human RANK monoclonal antibody (N1H8; Amgen Inc., 5 µg/ml), anti-human RANKL monoclonal antibody (Amgen Inc., M366, 1.85 µg/ml), anti-KI67 (SP6, Abcam; 1:200) and anti-cleaved caspase-3 (Asp175, Cell Signaling; 1:200) antibodies were used. VECTASTAIN® Elite® ABC-HRP Kit (Vector Laboratories) was used to amplify the RANK, RANKL and cleaved caspase-3 signal. Images were analyzed with Fiji software (Schindelin *et al*, 2012).

RANKL stimulation *in vitro*

PDX single tumor cells were embedded in Corning™ Matrigel™ Growth Factor Reduced Basement Membrane Matrix (Corning™ 356,238), plated in DMEM/F-12 with B-27™ Supplement, EGF 10 ng/ml, hydrocortisone 0.5 µg/ml, insulin 5 µg/ml, cholera toxin 100 ng/ml, and penicillin/streptomycin. Cells were stimulated, or not, with h-RANKL (500 ng/ml; Amgen Inc.) during 24 h prior to gene expression analyses.

Quantitative reverse transcription PCR (Q-RT-PCR)

Total RNA was isolated from tumor pieces using TRIzol (Thermo Fisher Scientific) or Maxwell® RSC simplyRNA Tissue Kit (AS1340 Promega). One microgram of RNA was reverse-transcribed into cDNA using 200 U Superscript II plus random hexamer oligos (Invitrogen). *RANK* and *RANKL* expression was amplified with LightCycler® 480 Probes Master (Roche, 04707494001) and a LightCycler® 480 thermocycler (Roche) and normalized relative to the *PPIA house keeper gene*. The primer sequences used were: *PPIA-UPL* (Fw: ATGCTGGACCCAA CACAAAT; Rv: TCTTTCACTTTGCCAAACACC), *TNFRSF11A-UPL* (Fw: GCAGGTGGCTTTGCAGAT; Rv: GCATTTAGAAGACATGTACT TTCCTG), *TNFSF11-UPL* (Fw: TGATTCATGTAGGAGAATTAAAC AGG; Rv: GATGTGCTGTGATCCAACGA).

Gene expression in organoid cultures was evaluated using SYBR Green Master I (Roche, 04887352001), LightCycler® 480 thermocycler (Roche). The primers used were: *PPIA* (Fw: ATGGTCAACCCCAC CGTT; Rv: TCTGCTGTCTTTGGGACCTTG), *TNFRSF11A* (Fw: ATCTG GGACGGTGCTGTAAC; Rv: GGCCTTGCCTGTATCACAAA), *TNFSF11* (Fw: TGATTCATGTAGGAGAATTAAACAGG; Rv: GATGTGCTGTGAT CCAACGA), *BIRC3* (Fw: GGTAACAGTGATGATGTCAAATG; Rv: TAA CTGGCTTGAACTTGACG), *ICAM1* (Fw: AACTGACACCTTTGTTAGC CACCTC; Rv: CCCAGTGAAATGCAAACAGGAC), *CCL2* (Fw: AGGTG ACTGGGGCATTGAT; Rv: GCCTCCAGCATGAAAGTCT), *CXCL8* (Fw: CTGCGCCAACACAGAAATTA; Rv: CATCTGGCAACCCTACAACA), *RELB* (Fw: CCCGACCTCTCCTCACTCTC; Rv: CAGGGTGACCGTGCT CAG), *NF-kB2* (Fw: GGCGGGCGTCTAAAATTCTG; Rv: TCCAGACC TGGGTTGTAGCA).

Western blot

PDX-derived cells were seeded in growth medium (5% FBS, EGF 10 ng/ml, hydrocortisone 0.5 µg/ml, insulin 5 µg/ml, cholera toxin 100 ng/ml, and penicillin/streptomycin) overnight. The following day, cells were serum starved (growth medium containing 0.5% FBS) for 24 h before RANKL stimulation (500 ng/ml; Amgen Inc.). Extracts for immunoblots were prepared with modified RIPA buffer (50 mM Tris pH 7.4, 150 nM NaCl, 1% Triton NP-40, 0.25% sodium deoxycholate) containing PhosSTOP and Complete protease inhibitor cocktail (Roche). Protein concentration was measured with DC protein assay reagent (Bio-Rad) and 40 µg of total protein were resolved by SDS–PAGE and transferred to Immobilon-P 0.45 µm membranes (Millipore). Primary antibodies against P-p65 (Ser536, Cell Signaling: 1:500), p65 (D14E12, Cell Signaling; 1:1,000), P-IkBα (S32/36, Cell Signaling; 1:500), IkBα (L35A5, Cell Signaling; 1:500), and β-tubulin (ab21058, Abcam; 1:5,000) were used. Blots were incubated with HRP-conjugated secondary antibodies (DAKO) and developed with ECL detection kit (Amersham Biosciences).

RNA sequencing

Total RNA samples were processed with the "QuantSeq 3′ mRNA-Seq Library Prep Kit (FWD) for Illumina" (Lexogen, Cat.No. 015)

© 2023 The Authors

with RNA Quality scores of 7.7 on average (range 4.2–9.2). Library generation was initiated by reverse transcription with oligodT priming, and a second strand synthesis was performed from random primers. Libraries were completed by PCR. cDNA libraries were purified, applied to an Illumina flow cell for cluster generation and sequenced on an Illumina instrument. Read adapters and poly A tails were removed with BBDuk v38.38. Then, human reads were separated from mice ones using Xenome v1.0.1 (Conway *et al*, 2012) and those classified as "human," "both" or "ambiguous" were selected. Processed reads were analyzed with the Nextpresso pipeline v1.9.2.5 (Graña *et al*, 2018). Sequencing quality was checked with FastQC v0.11.7 and FastQ Screen v0.13.0. Reads were aligned to the human reference genome (GRCh38) with TopHat v2.0.10 using Bowtie v1.0.0.0 and Samtools v0.1.19.0 (—library-type fr-secondstrand), allowing three mismatches and 20 multihits. Read counts were obtained with HTSeq-count v0.6.1 (—-stranded = yes) using the human gene annotation from GENCODE (gencode.v34.GRCh38.Ensembl100). Differential expression was performed with DESeq2, using a 0.05 FDR. Genes were ranked according to the log2 Fold Change and GSEAPreranked v2.2.2 was used to perform gene set enrichment analysis for Hallmark, Biocarta, Reactome and KEGG v7.1 signatures, setting 1,000 gene set permutations and a classic enrichment statistic. Only those signatures with significant enrichment levels (FDR q-value < 0.25) were considered.

Statistical analysis

Statistical analysis in the TMA collections was performed with the support of the IDIBELL and Nottingham University Statistical Assessment Services. Associations between IHC scores and clinicopathologic parameters were evaluated using Pearson's Chi-Square test or Fisher's exact test. Tumor samples with less than 30% tumor cells were excluded from the analyses. RANK and RANKL were scored blindly to the tumor clinical and pathological characteristics. BCSS, DMFS and DFS were analyzed using the Kaplan–Meier function, Cox regression analyses and the log rank test. Data analyses of mouse experiments were performed using GraphPad Prism software version 8. Regression analysis of the growth curve mean for *in vivo* treatments was performed. Analysis of the differences between two conditions was performed with a two-tailed Student's *t*-test.

Bubble matrix plots were drawn using R (v4.0.3) and ggplot2 (v3.3.3). These plots represent the NES and FDR values reported by GSEA for some selected pathways in all tested comparisons. The color scale represents the NES: red denotes a NES > 0 and blue a NES < 0. The more intense the color, the more extreme the NES. In addition, the size of the bubble is proportional to the $-\log10$ of the FDR. Thus, the bigger the dot, the smaller the FDR. Gene sets were classified according to Pearson's R coefficient generated by public gene set databases (KEGG, Biocarta, Reactome, and Hallmarks). For *in vivo* experiments, mice showing a non-complete recovery after surgery were excluded from the study. Mice were randomized into the different treatment groups when the tumor reached 5×5 mm and tumor monitoring was done in a blinded fashion.

Study approval

All human samples were obtained following institutional guidelines, study received approval form the corresponding institutional Ethics Committee, and the experiments conformed to the principles set out in the WMA Declaration of Helsinki and the Department of Health and Human Services Belmont Report. This work obtained ethics approval to use the human tissue samples by the corresponding institutional review boards: Greater Manchester Central Research Ethics Committee reference number 15/NW/0685 (Nottingham); Hospital Universitario 12 de Octubre, number 11/137 (CNIO) and Hospital Universitario de Bellvitge, PR166/11071/015. Informed consent was obtained from all individuals prior to surgery to use their tissue materials in research. Written informed consent for PDX generation was obtained from all subjects. All experimental animal procedures were performed according to Spanish regulations. All research involving animals was performed at the IDIBELL and CNIO animal facilities in compliance with protocols approved by the IDIBELL Committee on Animal Care and the Directorate-General for Agricultural Production of the Ministry of Agriculture and Livestock Farming (PROEX_161.2/21), respectively, following national and European Union regulations.

The paper explained

Problem

The search for new prognostic factors and therapeutic targets has become an essential task for the individualization of breast cancer therapy. RANK signaling pathway has emerged as a new target for breast cancer based on compelling preclinical evidence. RANKL inhibition prevents or attenuates mammary tumor initiation, and induces tumor cell differentiation and an anti-tumorigenic immune response in established tumors. However, in clinical trials the therapeutic benefit of the RANKL inhibitor denosumab in breast cancer, beyond its bone-related effects, is unclear. Given the heterogeneity of breast cancer, a better understanding of RANK biology is needed to identify the patients who may benefit from denosumab.

Results

Here, we report the expression patterns of RANK and RANKL proteins in more than 2,000 breast tumor samples from independent collections, together with functional studies in breast cancer patient-derived xenografts (PDXs). Our results demonstrate that RANK protein expression in tumor cells constitutes a new independent biomarker of poor prognosis in patients with ER^- tumors and in postmenopausal women. Accordingly, RANKL inhibition improves response to chemotherapy in ER^- BC PDXs, reducing recurrence. The distinct biology of RANK signaling according to ER expression and menopause enlighten these results: RANK activation increases in tumors after menopause and regulates tumor cell metabolism in ER^- disease.

Impact

Our findings identify RANK as a new biomarker of poor prognosis in postmenopausal women with ER^- breast tumors. These results will help to identify breast cancer patients who can benefit from denosumab in a personalized therapeutic strategy.

Data availability

RNAseq results have been deposited in GEO: GSE185513 study (https://www.ncbi.nlm.nih.gov/geo/query/acc.cgi?acc=GSE185513).

Expanded View for this article is available online.

© 2023 The Authors

Acknowledgements

We would like to thank all the patients who contributed to this study, Robert Clarke, Bruno Simoes (University of Manchester), Joaquin Arribas (VHIO), Alana Welm (Huntsman Cancer institute), Violeta Serra (VHIO) and Carlos Caldas (CRUK, University of Cambridge) for providing PDX tumor samples for the analyses of RANK and RANKL expression. We thank Amgen Inc. for providing the N1H8 and M366 antibodies and the recombinant RANKL and RANK-Fc proteins. We would like to thank the IDIBELL and CNIO Animal Facility for their assistance with mouse colonies, Esther Castaño, Beatriz Barroso and the scientific services of the University of Barcelona for their assistance with flow cytometry analyses, Fran Cimas, Elayne Hondares, Idoia Morilla for the analyses of TMA and PDX cohorts, Manuel Gris. Sergi Velasco for NSG maintenance and analyses, Estefania Ayala and Dr. Sagrario Ortega of the Mouse Genome Editing for their experimental assistance. We thank Miguel Angel Pujana, Christian Tebe and Judith Peñafiel for the analyses of the *IDB* and *CNIO* cohorts, Sergi Velasco and Purificación Muñoz for feedback about the manuscript. Work in the laboratory of EG-S has received funding from the Spanish Ministerio de Ciencia, Innovación y Universidades, which is part of Agencia Estatal de Investigación (AEI) (SAF2014-55997-R, SAF2017-86117-R, and PID2020-116441GB-I00), the ISCIII (PIE13/00022) co-funded by European Regional Development Fund, a Career Catalyst Grant from the Susan Komen Foundation (CCR13262449), the Catalan Government 2017SGR00665, co-funded by FEDER funds/European Regional Development Fund (ERDF) (a way to build Europe), the European Research Council (ERC) under the European Union's Horizon 2020 research and innovation program (grant agreement no. 682935), This study has been partially funded by Amgen Inc. EMT had received a Juan de la Cierva-Incorporación grant from Spanish Ministry of Science and Innovation (IJCI-2017-31564) and MG a PERIS contract from the Departament of Salut de la Generalitat de Catalunya. The IDIBELL samples collection was granted by the Spanish Ministry of Health and Consumer Affairs FIS-PI14/00336 from the I + D + I National Plan with the financial support from ISCIII-Subdirección General de Evaluación and the Fondo Europeo de Desarrollo Regional (FEDER). The generation of PDX models in the Lewis laboratory was supported in part by NIH/NCI grants U54 CA224076 (MTL), U24 CA226110 (to MTL), P50 CA186784 (MTL) (Dan L. Duncan Cancer Center). This work was also supported by a Core Facility grant from the Cancer Prevention and Research Institute of Texas (CPRIT Core Facilities Support Grant RP170691).

Author contributions

Marina Ciscar: Data curation; formal analysis; investigation; methodology; writing – original draft; writing – review and editing. **Eva M Trinidad:** Data curation; formal analysis; investigation; methodology; writing – original draft; writing – review and editing. **Gema Perez-Chacon:** Data curation; formal analysis; investigation; writing – original draft; writing – review and editing. **Hector Perez-Montoyo:** Data curation; formal analysis; investigation; writing – review and editing. **Maria Jimenez:** Data curation; formal analysis; investigation; writing – original draft; writing – review and editing. **Mansour Alsaleem:** Formal analysis; writing – review and editing. **Maria J Jimenez-Santos:** Formal analysis; writing – review and editing. **Adrian Sanz-Moreno:** Data curation; formal analysis; writing – review and editing. **Andrea Vethencourt:** Data curation; formal analysis; writing – review and editing. **Michael Toss:** Formal analysis; writing – review and editing. **Anna Petit:** Data curation; formal analysis; writing – review and editing. **Maria Teresa Soler-Monso:** Formal analysis; writing – review and editing. **Victor Lopez:** Data curation; writing – review and editing. **Jorge Gomez-Miragaya:** Data curation; writing – review and editing. **Clara Gomez-Aleza:** Data curation; writing – review and editing. **Lacey E Dobrolecki:** Resources. **Michael T Lewis:** Resources. **Alejandra Bruna:** Resources. **Silvana Mouron:** Resources. **Miguel Angel Quintela-Fandino:** Resources. **Fátima Al-Shahrour:** Formal analysis; writing – review and editing. **Antonio Martinez-Aranda:** Resources. **Angels Sierra:** Resources. **Andrew R Green:** Formal analysis. **Emad Rakha:** Resources; formal analysis; writing – review and editing. **Eva Gonzalez-Suarez:** Conceptualization; resources; data curation; formal analysis; supervision; funding acquisition; investigation; methodology; writing – original draft; project administration; writing – review and editing.

Disclosure and competing interests statement

EG-S has served on advisory boards for Amgen Inc. and has received honoraria and research funding from Amgen Inc. MTL is a Founder of, and an uncompensated Manager in StemMed Holdings L.L.C., an uncompensated Limited Partner in StemMed Ltd., and is a Founder of and equity stake holder in Tvardi Therapeutics. LED is a compensated employee of StemMed Ltd. Selected BCM PDX models described herein are exclusively licensed to StemMed Ltd., resulting in tangible property royalties to MTL and LED.

References

Abd El-Rehim DM, Ball G, Finder SE, Rakha E, Paish C, Robertson JFR, Macmillan D, Blamey RW, Ellis IO (2005) High-throughput protein expression analysis using tissue microarray technology of a large well-characterised series identifies biologically distinct classes of breast cancer confirming recent cDNA expression analyses. *Int J Cancer* 116: 340–350

Azim HA, Peccatori FA, Brohée S, Branstetter D, Loi S, Viale G, Piccart M, Dougall WC, Pruneri G, Sotiriou C (2015) RANK-ligand (RANKL) expression in young breast cancer patients and during pregnancy. *Breast Cancer Res* 17: 24

Benítez S, Cordero A, Santamaría PG, Redondo-Pedraza J, Rocha AS, Collado-Solé A, Jimenez M, Sanz-Moreno A, Yoldi G, Santos JC *et al* (2021) RANK links senescence to stemness in the mammary epithelia, delaying tumor onset but increasing tumor aggressiveness. *Dev Cell* 56: 1727–1741.e7

Blohmer JU, Link T, Reinisch M, Just M, Untch M, Stötzer O, Fasching PA, Schneeweiss A, Wimberger P, Seiler S *et al* (2022) Effect of denosumab added to 2 different nab-paclitaxel regimens as neoadjuvant therapy in patients with primary breast cancer: the GeparX 2 × 2 randomized clinical trial. *JAMA Oncol* 8: 1010–1018

Bruna A, Rueda OM, Greenwood W, Batra AS, Callari M, Batra RN, Pogrebniak K, Sandoval J, Cassidy JW, Tufegdzic-Vidakovic A *et al* (2016) A biobank of breast cancer explants with preserved intra-tumor heterogeneity to screen anticancer compounds. *Cell* 167: 260–274.e22

Cheang MCU, Chia SK, Voduc D, Gao D, Leung S, Snider J, Watson M, Davies S, Bernard PS, Parker JS *et al* (2009) Ki67 index, HER2 status, and prognosis of patients with luminal B breast cancer. *J Natl Cancer Inst* 101: 736–750

Chukir T, Liu Y, Farooki A (2019) Antiresorptive agents' bone-protective and adjuvant effects in postmenopausal women with early breast cancer. *Br J Clin Pharmacol* 85: 1125–1135

Coleman R, Finkelstein DM, Barrios C, Martin M, Iwata H, Hegg R, Glaspy J, Periañez AM, Tonkin K, Deleu I *et al* (2020) Adjuvant denosumab in early breast cancer (D-CARE): an international, multicentre, randomised, controlled, phase 3 trial. *Lancet Oncol* 21: 60–72

Conway T, Wazny J, Bromage A, Tymms M, Sooraj D, Williams ED, Beresford-Smith B (2012) Xenome-a tool for classifying reads from xenograft samples. *Bioinformatics* 28: 172–178

Curtis C, Shah SP, Chin SF, Turashvili G, Rueda OM, Dunning MJ, Speed D, Lynch AG, Samarajiwa S, Yuan Y *et al* (2012) The genomic and

© 2023 The Authors

transcriptomic architecture of 2,000 breast tumours reveals novel subgroups. *Nature* 486: 346 – 352

Dent R, Trudeau M, Pritchard KI, Hanna WM, Kahn HK, Sawka CA, Lickley LA, Rawlinson E, Sun P, Narod SA (2007) Triple-negative breast cancer: clinical features and patterns of recurrence. *Clin Cancer Res* 13: 4429 – 4434

Derose YS, Wang G, Lin YC, Bernard PS, Buys SS, Ebbert MTW, Factor R, Matsen C, Milash BA, Nelson E *et al* (2011) Tumor grafts derived from women with breast cancer authentically reflect tumor pathology, growth, metastasis and disease outcomes. *Nat Med* 17: 1514 – 1520

Elston CW, Ellis IO (1991) Pathological prognostic factors in breast cancer. I. the value of histological grade in breast cancer: experience from a large study with long-term follow-up. *Histopathology* 19: 403 – 410

Eyre R, Alférez DG, Spence K, Kamal M, Shaw FL, Simões BM, Santiago-Gómez A, Sarmiento-Castro A, Bramley M, Absar M *et al* (2016) Patient-derived mammosphere and xenograft tumour initiation correlates with progression to metastasis. *J Mammary Gland Biol Neoplasia* 21: 99 – 109

Gnant M, Pfeiler G, Steger GG, Egle D, Greil R, Fitzal F, Wette V, Balic M, Haslbauer F, Melbinger-Zeinitzer E *et al* (2018) Adjuvant denosumab in early breast cancer: disease-free survival analysis of 3,425 postmenopausal patients in the ABCSG-18 trial. *J Clin Oncol* 36: 500

Gómez-Aleza C, Nguyen B, Yoldi G, Ciscar M, Barranco A, Hernández-Jiménez E, Maetens M, Salgado R, Zafeiroglou M, Pellegrini P *et al* (2020) Inhibition of RANK signaling in breast cancer induces an anti-tumor immune response orchestrated by CD8+ T cells. *Nat Commun* 11: 1 – 18

Gómez-Miragaya J, Palafox M, Paré L, Yoldi G, Ferrer I, Vila S, Galván P, Pellegrini P, Pérez-Montoyo H, Igea A *et al* (2017) Resistance to Taxanes in triple-negative breast cancer associates with the dynamics of a CD49f+ tumor-initiating population. *Stem Cell Reports* 8: 1392 – 1407

Gonzalez-Suarez E, Jacob AP, Jones J, Miller R, Roudier-Meyer MP, Erwert R, Pinkas J, Branstetter D, Dougall WC (2010) RANK ligand mediates progestin-induced mammary epithelial proliferation and carcinogenesis. *Nature* 468: 103 – 107

Graña O, Rubio-Camarillo M, Fdez-Riverola F, Pisano DG, Glez-Peña D (2018) Nextpresso: next generation sequencing expression analysis pipeline. *Curr Bioinform* 13: 583 – 591

Green AR, Powe DG, Rakha EA, Soria D, Lemetre C, Nolan CC, Barros FFT, MacMillan RD, Garibaldi JM, Ball GR *et al* (2013) Identification of key clinical phenotypes of breast cancer using a reduced panel of protein biomarkers. *Br J Cancer* 109: 1886 – 1894

Gris-Oliver A, Palafox M, Monserrat L, Braso-Maristany F, Odena A, Sanchez-Guixe M, Ibrahim YH, Villacampa G, Grueso J, Pares M *et al* (2020) Genetic alterations in the PI3K/AKT pathway and baseline AKT activity define AKT inhibitor sensitivity in breast cancer patient-derived xenografts. *Clin Cancer Res* 26: 3720 – 3731

Joshi PA, Jackson HW, Beristain AG, Di Grappa MA, Mote PA, Clarke CL, Stingl J, Waterhouse PD, Khokha R (2010) Progesterone induces adult mammary stem cell expansion. *Nature* 465: 803 – 807

Khosla S, Oursler MJ, Monroe DG (2012) Estrogen and the skeleton. *Trends Endocrinol Metab* 23: 576 – 581

Martínez-Aranda A, Hernández V, Guney E, Muixí L, Foj R, Baixeras N, Cuadras D, Moreno V, Urruticoechea A, Gil M *et al* (2015) FN14 and GRP94 expression are prognostic/predictive biomarkers of brain metastasis outcome that open up new therapeutic strategies. *Oncotarget* 6: 44254 – 44273

Miyazaki T, Tokimura F, Tanaka S (2014) A review of denosumab for the treatment of osteoporosis. *Patient Prefer Adherence* 8: 463 – 471

Nolan E, Vaillant F, Branstetter D, Pal B, Giner G, Whitehead L, Lok SW, Mann GB, Rohrbach K, Huang LY *et al* (2016) RANK ligand as a potential target for breast cancer prevention in BRCA1-mutation carriers. *Nat Med* 22: 933 – 939

Palafox M, Ferrer I, Pellegrini P, Vila S, Hernandez-Ortega S, Urruticoechea A, Climent F, Soler MT, Muñoz P, Viñals F *et al* (2012) RANK induces epithelial-mesenchymal transition and stemness in human mammary epithelial cells and promotes tumorigenesis and metastasis. *Cancer Res* 72: 2879 – 2888

Perou CM, Sørile T, Eisen MB, Van De Rijn M, Jeffrey SS, Ress CA, Pollack JR, Ross DT, Johnsen H, Akslen LA *et al* (2000) Molecular portraits of human breast tumours. *Nature* 406: 747 – 752

Perrone F, Gravina A (2020) Denosumab in early breast cancer: negative data and a call to action. *Lancet Oncol* 21: 5 – 6

Pfitzner BM, Branstetter D, Loibl S, Denkert C, Lederer B, Schmitt WD, Dombrowski F, Werner M, Rüdiger T, Dougall WC *et al* (2014) RANK expression as a prognostic and predictive marker in breast cancer. *Breast Cancer Res Treat* 145: 307 – 315

Rakha EA, El-Sayed ME, Lee AHS, Elston CW, Grainge MJ, Hodi Z, Blamey RW, Ellis IO (2008) Prognostic significance of Nottingham histologic grade in invasive breast carcinoma. *J Clin Oncol* 26: 3153 – 3158

Rao S, Sigl V, Wimmer RA, Novatchkova M, Jais A, Wagner G, Handschuh S, Uribesalgo I, Hagelkruys A, Kozieradzki I *et al* (2017) RANK rewires energy homeostasis in lung cancer cells and drives primary lung cancer. *Genes Dev* 31: 2099 – 2111

Schindelin J, Arganda-Carreras I, Frise E, Kaynig V, Longair M, Pietzsch T, Preibisch S, Rueden C, Saalfeld S, Schmid B *et al* (2012) Fiji: an open-source platform for biological-image analysis. *Nat Methods* 9: 676 – 682

Schramek D, Leibbrandt A, Sigl V, Kenner L, Pospisilik JA, Lee HJ, Hanada R, Joshi PA, Aliprantis A, Glimcher L *et al* (2010) Osteoclast differentiation factor RANKL controls development of progestin-driven mammary cancer. *Nature* 468: 98 – 102

Smalley MJ (2010) Isolation, culture and analysis of mouse mammary epithelial cells. *Methods Mol Biol* 633: 139 – 170

Streicher C, Heyny A, Andrukhova O, Haigl B, Slavic S, Schüler C, Kollmann K, Kantner I, Sexl V, Kleiter M *et al* (2017) Estrogen regulates bone turnover by targeting RANKL expression in bone lining cells. *Sci Rep* 7: 6460

Weigel MT, Dowsett M (2010) Current and emerging biomarkers in breast cancer: prognosis and prediction. *Endocr Relat Cancer* 17: R245 – R262

Yoldi G, Pellegrini P, Trinidad EM, Cordero A, Gomez-Miragaya J, Serra-Musach J, Dougall WC, Muñoz P, Pujana MA, Planelles L *et al* (2016) RANK signaling blockade reduces breast cancer recurrence by inducing tumor cell differentiation. *Cancer Res* 76: 5857 – 5869

Zhang X, Lewis MT (2013) Establishment of patient-derived xenograft (PDX) models of human breast cancer. *Curr Protoc Mouse Biol* 3: 21 – 29

License: This is an open access article under the terms of the Creative Commons Attribution License, which permits use, distribution and reproduction in any medium, provided the original work is properly cited.

Expanded View Figures

Figure EV1. RANK is expressed in tumor and stromal cells of human BC and associates with ER/PR-negative tumors.

A Percentage of patients expressing RANK or tmRANKL in tumor and stromal cells in BC samples. The total number of patients is indicated.
B H-Score values of tumor RANK and tmRANKL from *IDB*, *NPS* collections and the *METABRIC* dataset. Mean ± SEM is shown.
C Percentage of BC patients with $RANK^+$ tumors according to the indicated clinicopathologic parameters in the *METABRIC* cohort. Total number of analyzed patients per parameter and *p*-values (Pearson's Chi-Square test (Exact Sig. 2- Side)) are indicated.

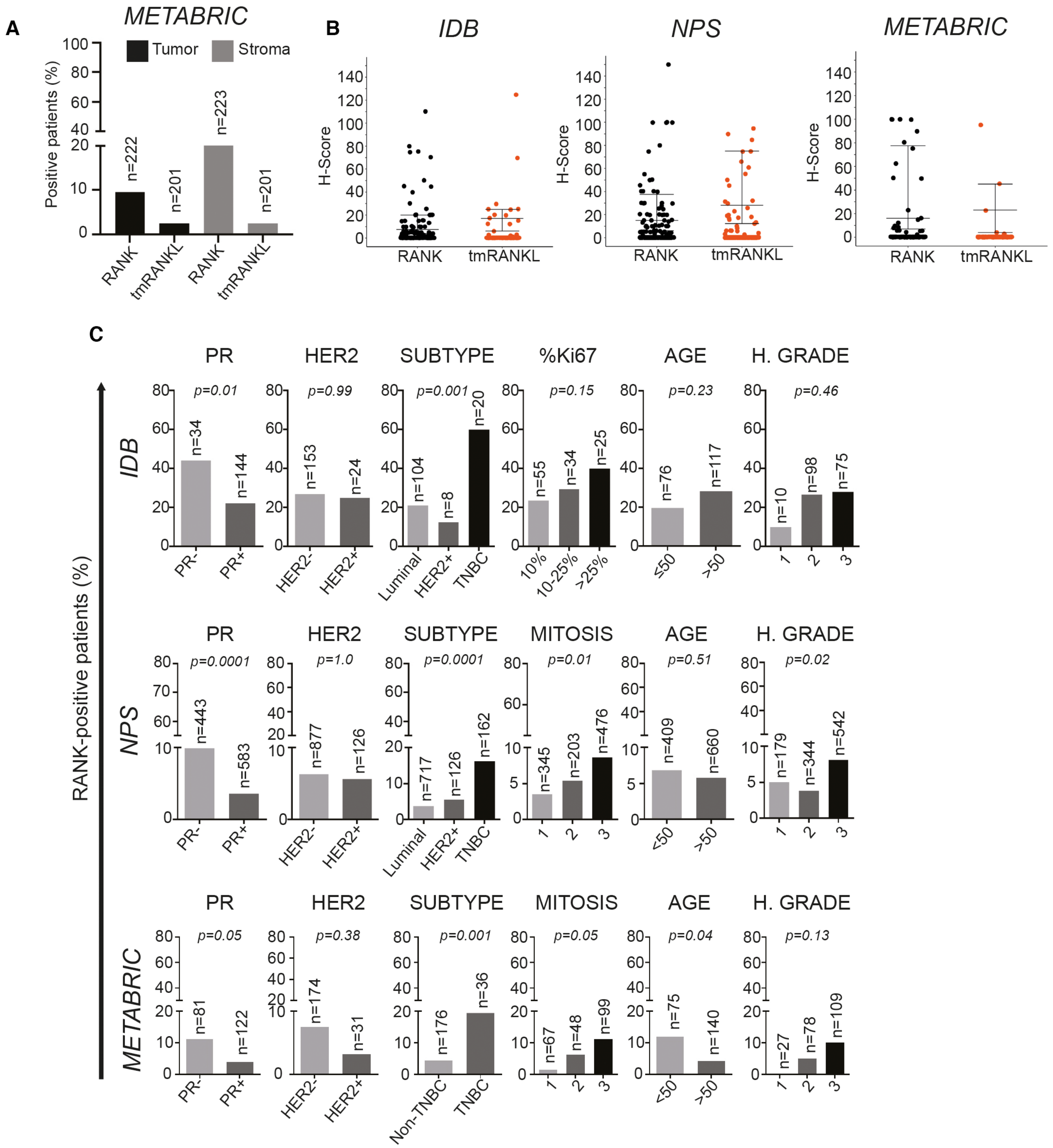

Figure EV1.

© 2023 The Authors

Figure EV2. RANK is expressed and active in a subset of BC PDXs.

A RANK and RANKL mRNA expression levels relative to *PPIA* in the indicated BC PDXs, organized according to ER status in the human tumor of origin and *RANK* mRNA expression. Two-tailed *t*-student test was used to evaluate the RANK/RANKL differential expression between ER^{-} and ER^{+} BC PDXs. # Indicates models where RANK and RANKL expression were analyzed by IHC.

B Representative images of RANK and RANKL protein expression in BC PDXs detected by IHC. H-Score (H) of the models (and not of the picture) are shown. A total of 3–5 independent tumors per PDX were scored for RANK.

C Western blot analyses of P-p65, P-IKBα and corresponding total proteins after RANKL stimulation in the indicated PDXs. Tubulin was used as a loading control.

D Gene expression analyses of the indicated NFKB target genes in PDX tumor organoids after 24 h of RANKL stimulation. Expression levels relative to the untreated controls are shown. Each dot represents organoids from an independent BC PDX tumor. Mean ± SEM and *p*-value of two-tailed *t*-student test are shown.

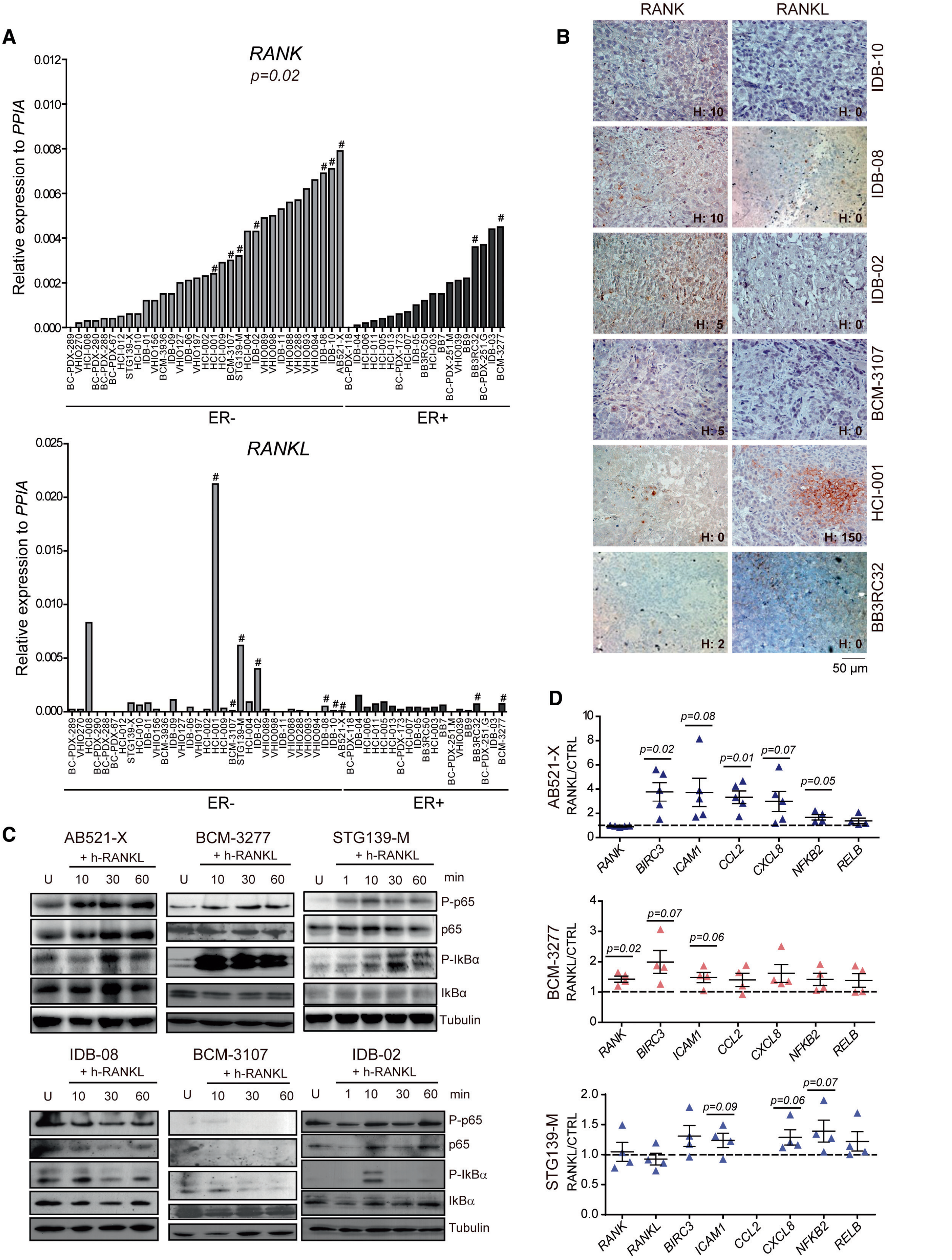

Figure EV2.

 © 2023 The Authors

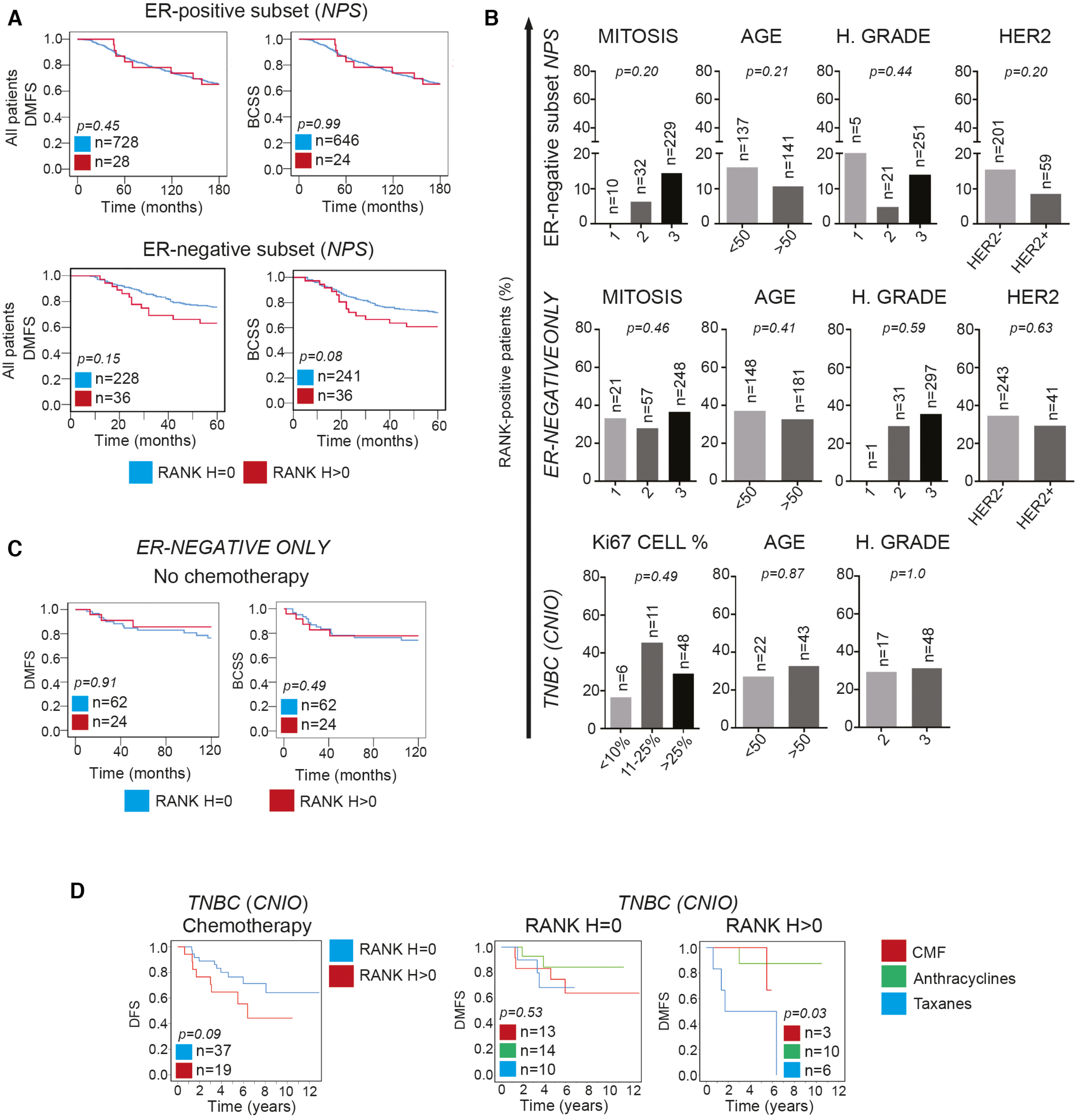

Figure EV3. RANK tumor expression is not related to any clinicopathologic factor in ER⁻ BC.

A BCSS and DMFS in the ER^+ and ER^- subsets from the *NPS* collection according to RANK expression in all patients.

B Percentage of $RANK^+$ BC patients according to the indicated clinicopathologic parameters in the three ER^- collections analyzed: *NPS* ER^- subset, *ER-NEGATIVE ONLY* and *TNBC (CNIO)*. Total number of analyzed patients per parameter and *p*-values (Pearson's Chi-Square test (Exact Sig. 2-side)) are indicated.

C DMFS and BCSS according to RANK expression in patients with ER^- tumors not treated with chemotherapy in the *ER-NEGATIVE ONLY* collection.

D RANK expression and DMFS in $RANK^-$ (H = 0) and $RANK^+$ (H > 0) tumor samples from the *TNBC (CNIO)* collection treated with chemotherapy and according to the chemotherapy regimen (group 1: CMF (cyclophosphamide, methotrexate, 5-fluorouracil); group 2: FAC (5-flurouracil, doxorubicin, cyclophosphamide) or FEC (5-fluorouracil, epirubicin and cyclophosphamide); and group 3: CMF or FAC or FEC plus taxanes).

Data information: (A, C, D) Total number of analyzed patients per parameter and *p*-values (Log-rank test (Mantel-Cox)) are indicated.

Figure EV4. RANKL inhibition in BC PDXs.

A Trap5b levels in mouse serum ($n = 2$) in tumor-bearing PDXs treated *in vivo* with RANK-Fc or DNS. Mean ± SD is shown.

B, C Representative images (B) and quantification (C) of KI67 and cleaved caspase-3 staining measured by IHC in $RANK^{+}$ tumors of PDXs treated *in vivo* with RANK-Fc or DNS. Each dot represents one picture. Three representative pictures per tumor were quantified and at least 3–4 tumors per condition were analyzed. Mean ± SEM and two-tailed *t*-test *p*-values are shown.

D Percentage of cells with ALDH activity in tumors isolated from the indicated PDXs. Each dot represents one tumor. Mean ± SEM and *p*-value of two-tailed *t*-student test are shown.

E Gene set enrichment analysis (GSEA) of associated genes after *in vivo* treatment with RANK-Fc in NSG mice, which are common for the 3 PDX models studied. The matrix illustrates NES and FDR values. The color scale represents the NES. The size of the bubbles is proportional to the -log10 of the FDR. For those signatures with an FDR = 0 after 1,000 permutations, we assigned an FDR = 10^-3 for visualization purposes. The signatures selected for this plot belong to Hallmark, Biocarta, Reactome and KEGG collections and have a reported FDR < 0.05 and a NES > 0 for all PDX models. The color legend indicates the main biological process associated with each signature.

Data information: (A–D) All the analysis were performed 24 h after last treatment.

© 2023 The Authors

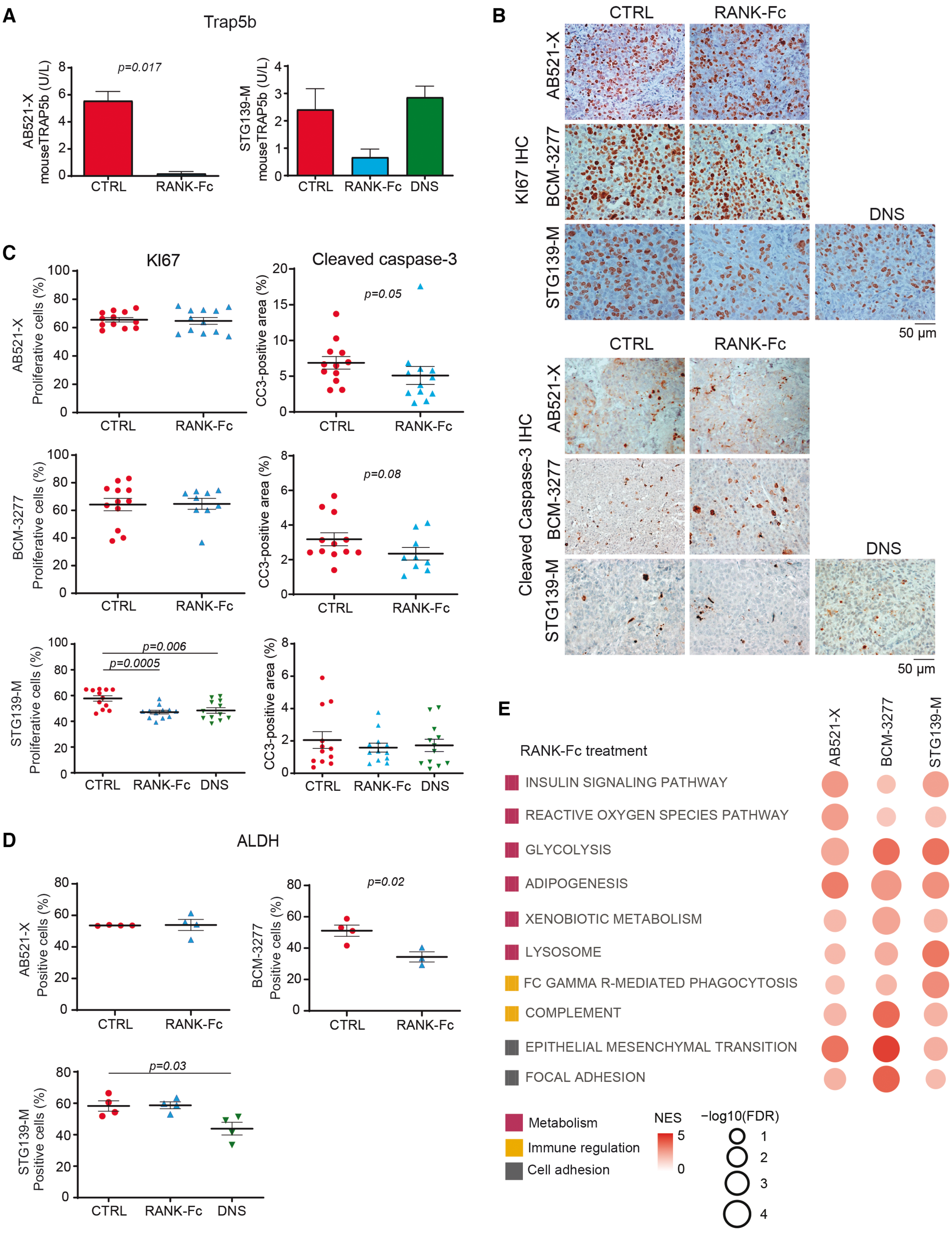
A
Trap5b
AB521-X mouseTRAP5b (U/L)
p=0.017
CTRL
RANK-Fc
STG139-M mouseTRAP5b (U/L)
CTRL RANK-Fc DNS
B
CTRL
RANK-Fc
DNS
KI67 IHC
AB521-X
BCM-3277
STG139-M
50 µm
Cleaved Caspase-3 IHC
C
KI67
Cleaved caspase-3
Proliferative cells (%)
CC3-positive area (%)
p=0.05
p=0.08
p=0.006
p=0.0005
D
ALDH
Positive cells (%)
p=0.02
p=0.03
E
RANK-Fc treatment
INSULIN SIGNALING PATHWAY
REACTIVE OXYGEN SPECIES PATHWAY
GLYCOLYSIS
ADIPOGENESIS
XENOBIOTIC METABOLISM
LYSOSOME
FC GAMMA R-MEDIATED PHAGOCYTOSIS
COMPLEMENT
EPITHELIAL MESENCHYMAL TRANSITION
FOCAL ADHESION
Metabolism
Immune regulation
Cell adhesion
NES
−log10(FDR)

Figure EV4.

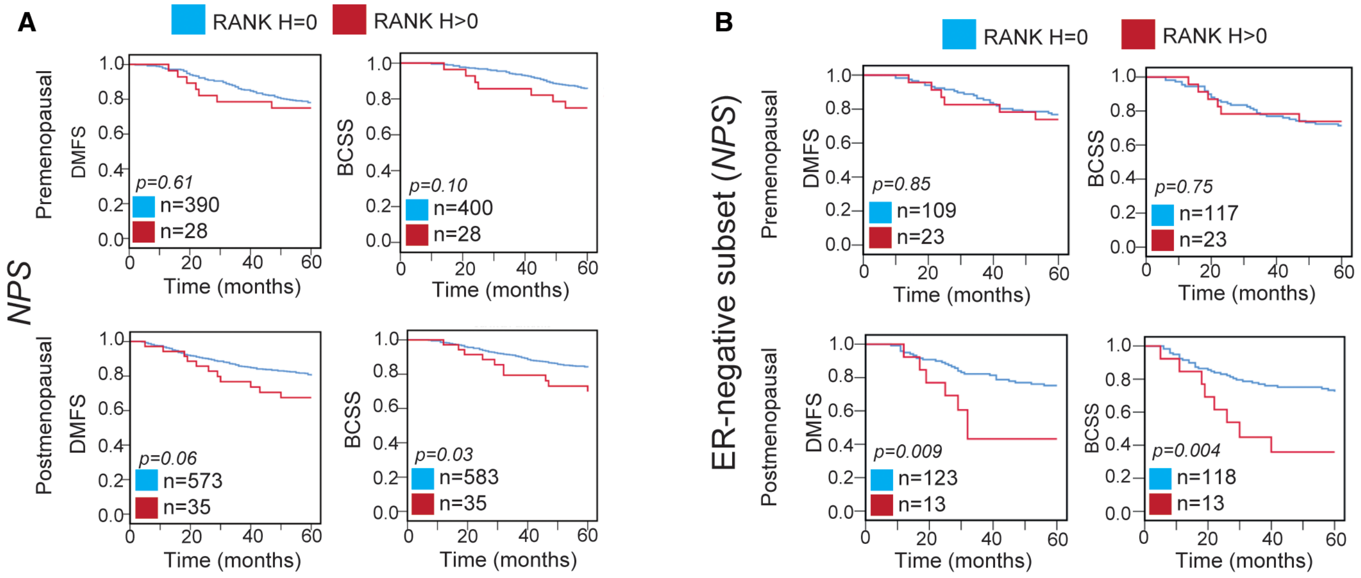

Figure EV5. RANK is a marker of poor prognosis in BC after menopause.

A, B DMFS and BCSS according to RANK expression ($RANK^-$ (H = 0) or $RANK^+$ (H > 0)) in premenopausal and postmenopausal patients of the *NPS* collection (A) and the *NPS* ER^- subset (B).

 © 2023 The Authors
