# Supplementary material for: RANK is a poor prognosis marker and a therapeutic target in ER‐negative postmenopausal breast cancer
Source: EMBO Mol Med. 2023 Mar 7;15(4):e16715. doi: 10.15252/emmm.202216715 (PMC10086586; doi:10.15252/emmm.202216715)
Supplement: Supplementary file 7 [file EMMM-15-e16715-s006.pdf]

EMBO Molecular Medicine

# RANK is a poor prognosis marker and a therapeutic target in ER-negative postmenopausal breast cancer

Marina Ciscar, Eva Trinidad, Gema Perez-Chacon, Hector Perez-Montoyo, Maria Jimenez, Mansour Alsaleem, Maria Jimenez-Santos, Adrian Sanz-Moreno, Andrea Vethencourt, Michael Toss, Anna Petit, Maria Teresa Soler-Monso, Victor Lopez, Jorge Gomez-Miragaya, Clara Gomez-Aleza, Lacey Dobrolecki, Michael Lewis, Alejandra Bruna, Silvana Mouron, Miguel Quintela-Fandino, Fátima Al-Shahrour, Antonio Martinez-Aranda, Angels Sierra, Andrew Green, Emad Rakha, and Eva Gonzalez-Suarez
**DOI: 10.15252/emmm.202216715**

*Corresponding author: Eva Gonzalez-Suarez (egonzalez@cnio.es)*



## Review Timeline:

| | |
|---|---|
| Submission Date: | 8th Aug 22 |
| Editorial Decision: | 9th Sep 22 |
| Revision Received: | 1st Dec 22 |
| Editorial Decision: | 16th Jan 23 |
| Revision Received: | 24th Jan 23 |
| Accepted: | 8th Feb 23 |

*Editor: Lise Roth*

## Transaction Report:

(Note: With the exception of the correction of typographical or spelling errors that could be a source of ambiguity, letters and reports are not edited. Depending on transfer agreements, referee reports obtained elsewhere may or may not be included in this compilation. Referee reports are anonymous unless the Referee chooses to sign their reports.)

9th Sep 2022

Dear Dr. Gonzalez-Suarez,

Thank you for the submission of your manuscript to EMBO Molecular Medicine, and please accept my apologies for the delay in getting back to you in this holiday season.
We have now received feedback from the three reviewers who agreed to evaluate your manuscript. As you will see from the reports below, the reviewers raise substantial concerns on your work, which unfortunately preclude its publication in EMBO Molecular Medicine in its current form.

The reviewers find that the question addressed by the study is of potential interest, however they remain unconvinced that some of the major conclusions are sufficiently supported by the data. They thus raise the following major issues:
- Small sample number
- Lack of mechanistic insight

After extensive discussion with my colleagues and further consultation with the referees, we would like to propose two different options:
- You may want to address these points and those listed by the referees, and submit a revised version of your manuscript as Research Article.
- However, we understand that this might require a lot of additional work and effort. Therefore, you could alternatively address the concerns raised by the referees with the exception of a detailed mechanistic insight, and submit a revised version as a Report. In this case, you should still discuss possible mechanisms/experiments that may explain your results.

Please attach a covering letter giving details of the way in which you have handled each of the points raised by the referees. A revised manuscript will once again be subject to review and we cannot guarantee at this stage that the eventual outcome will be favorable.

EMBO Molecular Medicine encourages a single round of revision only and therefore, acceptance or rejection of the manuscript will depend on the completeness of your responses included in the next, final version of the manuscript. For this reason, and to save you from any frustrations in the end, I would strongly advise against returning an incomplete revision.

Revised manuscripts should be submitted within three months of a request for revision; they will otherwise be treated as new submissions, except under exceptional circumstances in which a short extension is obtained from the editor.

When submitting your revised manuscript, please carefully review the instructions that follow below. We perform an initial quality control of all revised manuscripts before re-review; failure to include requested items will delay the evaluation of your revision.

We require:

1) A .docx formatted version of the manuscript text (including legends for main figures, EV figures and tables). Please make sure that the changes are highlighted to be clearly visible.

2) Individual production quality figure files as .eps, .tif, .jpg (one file per figure). For guidance, download the 'Figure Guide PDF' (https://www.embopress.org/page/journal/17574684/authorguide#figureformat).

3) A .docx formatted letter INCLUDING the reviewers' reports and your detailed point-by-point responses to their comments. As part of the EMBO Press transparent editorial process, the point-by-point response is part of the Review Process File (RPF), which will be published alongside your paper.

4) A complete author checklist, which you can download from our author guidelines (https://www.embopress.org/page/journal/17574684/authorguide#submissionofrevisions). Please insert information in the checklist that is also reflected in the manuscript. The completed author checklist will also be part of the RPF.

5) Please note that all corresponding authors are required to supply an ORCID ID for their name upon submission of a revised manuscript.

6) It is mandatory to include a 'Data Availability' section after the Materials and Methods. Before submitting your revision, primary datasets produced in this study need to be deposited in an appropriate public database, and the accession numbers and database listed under 'Data Availability'. Please remember to provide a reviewer password if the datasets are not yet public (see https://www.embopress.org/page/journal/17574684/authorguide#dataavailability).

In case you have no data that requires deposition in a public database, please state so in this section. Note that the Data Availability Section is restricted to new primary data that are part of this study.

7) For data quantification: please specify the name of the statistical test used to generate error bars and P values, the number (n) of independent experiments (specify technical or biological replicates) underlying each data point and the test used to calculate p-values in each figure legend. The figure legends should contain a basic description of n, P and the test applied. Graphs must include a description of the bars and the error bars (s.d., s.e.m.). Please provide exact p values.

8) We would also encourage you to include the source data for figure panels that show essential data. Numerical data should be provided as individual .xls or .csv files (including a tab describing the data). For blots or microscopy, uncropped images should be submitted (using a zip archive if multiple images need to be supplied for one panel). Additional information on source data and instruction on how to label the files are available at
.

9) Our journal encourages inclusion of *data citations in the reference list* to directly cite datasets that were re-used and obtained from public databases. Data citations in the article text are distinct from normal bibliographical citations and should directly link to the database records from which the data can be accessed. In the main text, data citations are formatted as follows: "Data ref: Smith et al, 2001" or "Data ref: NCBI Sequence Read Archive PRJNA342805, 2017". In the Reference list, data citations must be labeled with "[DATASET]". A data reference must provide the database name, accession number/identifiers and a resolvable link to the landing page from which the data can be accessed at the end of the reference. Further instructions are available at .

10) We replaced Supplementary Information with Expanded View (EV) Figures and Tables that are collapsible/expandable online. A maximum of 5 EV Figures can be typeset. EV Figures should be cited as 'Figure EV1, Figure EV2" etc... in the text and their respective legends should be included in the main text after the legends of regular figures.
- For the figures that you do NOT wish to display as Expanded View figures, they should be bundled together with their legends in a single PDF file called *Appendix*, which should start with a short Table of Content. Appendix figures should be referred to in the main text as: "Appendix Figure S1, Appendix Figure S2" etc.
- Additional Tables/Datasets should be labeled and referred to as Table EV1, Dataset EV1, etc. Legends have to be provided in a separate tab in case of .xls files. Alternatively, the legend can be supplied as a separate text file (README) and zipped together with the Table/Dataset file.
See detailed instructions here:
.

11) The paper explained: EMBO Molecular Medicine articles are accompanied by a summary of the articles to emphasize the major findings in the paper and their medical implications for the non-specialist reader. Please provide a draft summary of your article highlighting
- the medical issue you are addressing,
- the results obtained and
- their clinical impact.
This may be edited to ensure that readers understand the significance and context of the research. Please refer to any of our published articles for an example.

12) For more information: There is space at the end of each article to list relevant web links for further consultation by our readers. Could you identify some relevant ones and provide such information as well? Some examples are patient associations, relevant databases, OMIM/proteins/genes links, author's websites, etc...

13) Author contributions: CRediT has replaced the traditional author contributions section because it offers a systematic machine readable author contributions format that allows for more effective research assessment. Please remove the Authors Contributions from the manuscript and use the free text boxes beneath each contributing author's name in our system to add specific details on the author's contribution. More information is available in our guide to authors.

14) Conflict of interest: We updated our journal's competing interests policy in January 2022 and request authors to consider both actual and perceived competing interests. Please review the policy https://www.embopress.org/competing-interests and update your competing interests if necessary.

15) Every published paper now includes a 'Synopsis' to further enhance discoverability. Synopses are displayed on the journal webpage and are freely accessible to all readers. They include a short stand first (maximum of 300 characters, including space) as well as 2-5 one-sentences bullet points that summarizes the paper. Please write the bullet points to summarize the key NEW findings. They should be designed to be complementary to the abstract - i.e. not repeat the same text. We encourage inclusion of key acronyms and quantitative information (maximum of 30 words / bullet point). Please use the passive voice. Please attach these in a separate file or send them by email, we will incorporate them accordingly.

Please also suggest a striking image or visual abstract to illustrate your article as a PNG file 550 px wide x 300-600 px high.

16) As part of the EMBO Publications transparent editorial process initiative (see our Editorial at http://embomolmed.embopress.org/content/2/9/329), EMBO Molecular Medicine will publish online a Review Process File (RPF) to accompany accepted manuscripts.

In the event of acceptance, this file will be published in conjunction with your paper and will include the anonymous referee reports, your point-by-point response and all pertinent correspondence relating to the manuscript. Let us know whether you agree with the publication of the RPF and as here, if you want to remove or not any figures from it prior to publication.

Please note that the Authors checklist will be published at the end of the RPF.

EMBO Molecular Medicine has a "scooping protection" policy, whereby similar findings that are published by others during review or revision are not a criterion for rejection. Should you decide to submit a revised version, I do ask that you get in touch after three months if you have not completed it, to update us on the status.

I look forward to receiving your revised manuscript.

Yours sincerely,

Lise Roth

Lise Roth, PhD
Senior Editor
EMBO Molecular Medicine

***** Reviewer's comments *****

Referee #1 (Remarks for Author):

This is a potentially interesting results on the connection of RANK expression with response to chemotherapy in TNBC. The main thrust of the paper is shown in Figures 4-6: RANKL inhibitions cooperate with chemotherapy (docetaxel) to suppress ER-negative/RANK+ PDX growth (Fig 4); RANK tumor expression associates with poor survival in postmenopausal patients (Fig 5); and RANKL inhibition attenuates tumor growth of a RANK+ ER- BC PDX in ovariectomized mice (postmenopausal conditions). Although interesting, the results are descriptive and seem like the beginning of a good story, which need further development. Most importantly, the mechanistic basis for the effect of RANKL inhibition on survival of postmenopausal ER- RANK+ BC patients and for the cooperation with or potentiation of chemotherapy is lacking? Specifically:

- the mechanistic basis for the enhanced effect of RANKL-inhibition in ovariectomized mice should be explored/elucidated.
- There is a known crosstalk between ER and NFkB. Does ER suppress RANK in ER+ breast cancer or does high RANK suppress ER expression in TNBC? i.e. can the authors immunoblot for ER in figure 2D?
- Why ER- BC cells respond to ovariectomy?
- Postmenopausal ER+ breast cancer is treated with aromatase inhibitors. Would such inhibitors further enhance the effect of ovariectomy?
- Does RANK-shRNA have similar effect as RANKL-inhibitors
- Fig. 6 should be expanded to include additional PDXs

Other issues
• The Abstract is a bit sloppy going back and forth about the correlation of RANK expression with poor prognosis in ER-negative BC (first) and then with poor prognosis in postmenopausal BC (Second). These two may be combined.

"Our results demonstrate that. RANK protein expression..." - a full stop in the middle of a sentence.

One sentence is interrupted and another line starts as a new paragraph.

Abstract ends with no full stop.

•Figure 1 is not very informative; it interrupts the flow. It may be combined with Fig. 3 or moved to supplemental data, and Fig 3 presented as Fig. 1.

• Fig. 1d shows distant metastasis-free survival (DMFS) of all patients is worse for RANK+ patients but not significantly. The Hazard ratio should be provided.
As indicated by the authors, the worse OS of RANK+ patients (the only statistically significant cohort), and the poor DMFS may reflect the fact that RANK is a marker for TNBCs which have poor prognosis. If only TNBC patients are stratified based on RANK expression - is there any effect on clinical outcome? This analysis is shown later, in Fig. 3b bottom, where high RANK is associated with a moderate but not significant worse prognosis. Overall, the differences are not great, may be subtype-related, and are not entirely clear.

Notably, a KM-Plotter analysis (https://kmplot.com) shows no significant effect of RANK expression in TN - Basal-breast cancer, based on RNA expression. When protein levels are used - high RANK expression correlates with better prognosis. Can the authors confirm and discuss the discrepancy between this site vs their IHC data?

• "Tumors with the highest levels of RANK mRNA expression were found in the ER- subtype. Meanwhile".
- A correlation analysis between RANK and ER should be provided.

• page 6 "In all cohorts, RANK expression (H > 0) was significantly associated with ER/PR negativity and TNBC subtype, but not HER2, age, tumor size or stage. In the NPS collection, RANK expression was also associated with a higher mitosis rate and grade (Fig. 1d, Table S1).
- Shouldn't this be Fig. 1C?

• Fig. 2C -subtypes should be indicated

• Fig. 4 AB521-x - P=0.005 even though the curves are very close to each other compared to BCM3277 - P=0.024.
- Is this correct?

• Fig. 6 shows the results in ovariectomized NSG mice. To demonstrate specificity, the authors should show side-by-side the effect of RANKL inhibition alone in normal mice as shown in Fig. 4a.

Referee #2 (Comments on Novelty/Model System for Author):

RANK has been extensively studied in breast cancer.
The conclusions are drawn from very few patient samples.

Referee #2 (Remarks for Author):

In this manuscript, Ciscar et al. present evidence that RANK is a poor prognosis and a potential target in ER-negative breast cancer. Denosumab (DNS) a RANK ligand (RANKL) inhibitor has been used clinically in breast cancer (BC) for decades, in patients who have bone metastasis to prevent further loss of bone mass, most often associated with ER-positive breast cancers. DNS is also extensively used in patients with osteoporosis to prevent further bone density loss. Both RANK and the receptor of RANKL activity have been heavily published in BC, including by the authors.
The authors present data from two independent TMA collections for all BC subtypes from the IDIBELL (IDB) and the Nottingham series (NPS) s and a subset of the NPS which were part of the METRABIC study. The IHC from these data was not impressive for RANK protein expression. The number of RANK-positive was highest in the NPS than IDB study with The result for TNBC in both IRB and NPS subtypes was correlating with the highest expression of RANK. RANK expression has been shown to be a prognostic and predictive marker in breast cancer subtypes
Functional, validation for RANK was done by using several PDX models, the authors show that RANK is mostly restricted to ER-negative PDX tumors. Exposure to hRANKL activated the TNF/NFκB signaling pathway in three PDX models in vitro ER-negative. And In vivo PDX ER-negative tumor treated with RANK inhibitor (RANK-Fc) alone or in combination with docetaxel inhibition showed moderate effects on tumor growth. Nonetheless, combination with docetaxel decreases tumor growth. Comparisons using expression profiling with generated GSEA pathways utilizing: 1) METABRIC, PDX derived tumors inhibited for RANK activity and clinical trials for early breast cancer using denosumab concluded the RANK signature prevails in ER-negative BC above the ER-positive BC. The GSEA pathways /data sets/ survival outcome(s), along with premenopausal and postmenopausal status were integrated to further provide evidence that RANK is most prominently predictive in premeopsaul ER-negative BC. Suggesting that clinical work should target RANK as a precision target in premenopausal ER-negative breast cancer a very important aspect to meet clinic needs.

In general: This is an extensive study that provide evidence to convince us that RANK is important in ER-negative BC from a leader in the field. Yet several areas of concern as to the virtuosity of the study as to how widely to define ER-negative breast

cancer subtypes, that is within such a category you also may harbor other subtypes, e.g., TNBC, HER2, BRCA etc... The nomenclature used to draw conclusion between ER-negative and ER-positive BC cause concerns due to having analysis on limited sample size, that de-arms datas statistically insignificant. In the present form the manuscript is not acceptable and or will require major revisions.
Major/Minor issues:
1) The authors are well verse and know that BC subtypes is a heterogenous disease. ER+, PR+, ER+/PR+ HER2, Luminal A, Luminal B , Basal-like, BRCA, TNBC etc...
2) ER-negative can be reflective of several subtype for example, PR+ or HER2 or a TNBC they are all ER-negative so is RANK. As example of this in the Nottingham Series were histological grade is provide to be significant for RANK expression the data reflect only 61 patient samples out of 1,054.
3) Similarly with the "vascular invasion" significance is based on 4 positive patients to make ana argument for RANK expression to be important paler in vascular invasion.
4) Additionally BCSS and DMFS significance is driving by few sample number the ER-negative Nottingham Series 8 and 7.
5) The author never reflect the true number in the text they are significance but in very few samples.
6) The menopausal status for RANK expression the Nottingham Series for ER+ BC was significant with only 24 driving the postmenopausal significance. Yet the ER-negative cohort BC postmenopausal was not statistically significance. Is not the argument that RANK is important in ER-negative postmenopausal women? Please clarify.
7) On occasion this reviewer was not sure if the authors were speaking describing correct clinical data when this reviewer was looking at in supplemental tables. A possible suggestion a "red highlight" in the excel table sheet would facilitate finds the discussed results.
8) Figure 1: IHC for stroma vs tumor staining for RANK was not impressive as the staining looks like it is mostly stroma and no tumor. One sample of IHC does not represent the IDB and or NPS study to draw these conclusions. None of these results are in ER negative subtypes. SFS1A/B/C has no statistical significance shown.
9) Figure 2: The authors state that there is no "functionality of RANK in human BC..." there are at minimum 256 published articles on RANK in BC, and more than one has done functionality studies of RANK. Fig2a qPCR is of poor resolution.
10) Figure 2D why was no RANK western run of the PDX tumors? because IHC on the PDX not impressive and is the BCM3277 least impressive by IHC, yet is very responsive to hRANKL activation of TNF/NFKB signaling. SFS2B RANK in the STG139M is low vs that of BCM-3277 is high yet IHC is the reverse.
11) If BCM3277 was original an ER positive as stated then become a ER negative how do the authors know if PR or HER2 is not there as well? Or if it's truly TNBC? Same goes for the other PDX models used.
12) Rationale not provide as to why RNA seq was done on the PDX model with exposure to RANKL for one month.
13) Fig3E is followed by "Tumor stage independently associated with three survival parameters analyzed.." refer data to Table S1. Which specific comparisons where done did the authors use the COX NPS vs CP NPS tabs? Please clarify.
14) Fig4. In Fig 1 IHC show that PDX AB521-X has more RANK that STG139-M, both responded to hRANKL yest these same PDX is never treated with DNS why? Same goes for BCM-3277 .
15) Similar concerns arise in the combinatorial DTX studies. No rationale is given.
16) If you block with RANK-Fc why did the tumor proliferate? This is never discussed.
17) Section on pathways the authors state "PDX GSEA demonstrates....200 pathways differentially expresses. TS3 has allot dozens of tabs was not easy to identify, which it is please clarify and identify.
18) FigS5A TS3 "Immunity" pathways? Which comparison again dozen of tabs multiple comparison which one are the authors referring to? S5B RANKL inhibition in three PDX models which three is not clear.
19) RANKL is regulated by progesterone in mammary gland homeostasis whereas estradiol/opg is the inhibitor of RANK/RANKL signaling (reference 30). Should state that is in bone please clarify inference make it sound like it does so in mammary gland .
20) Fig5a Table S1 it was not clear if RANK was predicting DMFS and BCSS not clear from the data if it was refereeing to ER- or the ER+, please clarify.
21) Following Fig5B, data from Table S1 was sued to refence "survival of 15/20 years" yet on the data CNIO data was only for 12 years, please clarify, similarly please clarify which chemoresistance data is been represented, not clear which one if been used from the text or the Tables S1.
22) The rationale to compare PDX data (Fig5C Table S4) with that of the postmenopausal METABRIC data sets is not clear stated at all. How does a PDX in a NSG mice comparison work?
23) Fig6C data set not significant, why not use denosumab in the AB 521?

Referee #3 (Remarks for Author):

The manuscript is rather interesting as it looks at the role of RANK in breast cancer and leverages the large datasets to do so - but importantly the authors have followed up with wet work to test the hypotheses that they generated. They should be commended for this. Only minor concerns are listed that may aid in the manuscript:

1) Figure 1D - can you split this to the PAM50 subtypes and show that this is not simply a function of basal vs all other subtypes. It would be good to show here that RANK status within a subtype can show altered outcomes.

2) Figure 2 - I would suggest bringing some of the supplemental data for a non-responsive line as a control into the main figure

(2D).

3) RNAseq data - I was unable to review the data deposited to GeoDatasets. Please make a reviewer token available so that the data can be reviewed PRIOR to publication. An embargo until publication is fine, but please provide a reviewer link and token.

4) GSEA - again, I'd suggest bringing this into the main portion of the manuscript. Cut some of Figure 2C and bring some of the data from table S3 in as a GSEA based figure showing the random walk.

5) FIgure 3B does not indicate in the legend or on the figure which is RANK +ve or -ve

6) Figure 5 - again, split out the subtypes and do the appropriate statistical tests.

*Point by point response EMM-2022-16715*

Referee #1 (Remarks for Author):

This is a potentially interesting results on the connection of RANK expression with response to chemotherapy in TNBC. The main thrust of the paper is shown in Figures 4-6: RANKL inhibitions cooperate with chemotherapy (docetaxel) to suppress ER-negative/RANK+ PDX growth (Fig 4); RANK tumor expression associates with poor survival in postmenopausal patients (Fig 5); and RANKL inhibition attenuates tumor growth of a RANK+ ER- BC PDX in ovariectomized mice (postmenopausal conditions). Although interesting, the results are descriptive and seem like the beginning of a good story, which need further development. Most importantly, the mechanistic basis for the effect of RANKL inhibition on survival of postmenopausal ER- RANK+ BC patients and for the cooperation with or potentiation of chemotherapy is lacking?

Specifically:
- the mechanistic basis for the enhanced effect of RANKL-inhibition in ovariectomized mice should be explored/elucidated.

*We agree with the referee that it is highly relevant to explore further the mechanism underlying the distinct biology of RANK signaling in postmenopausal conditions and the greater effect in ovariectomized mice. This is an ongoing line of research in the laboratory. We are using additional experimental models and clinical samples from our ongoing clinical trial, D-BIOMARK for this purpose. Our current hypothesis is that differences in tumor cell metabolism driven by RANK contribute to the greater effect observed in postmenopausal conditions. Preliminary results support that the drop in estradiol changes systemic metabolism but also tumor cell metabolism driven by RANK. Additional mechanisms may include: enhanced activation of RANK signaling in the tumors after menopause that would make them more responsive to RANKL inhibition, putative cooperation of RANKL inhibitors with soluble factors released from the bone. Additional work that extends beyond this revision is required to provide a solid mechanism. For this reason, as suggested by the editors, we have submitted the revised manuscript as a Report, where we discuss potential mechanisms contributing to these differences.*

- There is a known crosstalk between ER and NFkB. Does ER suppress RANK in ER+ breast cancer or does high RANK suppress ER expression in TNBC? i.e. can the authors immunoblot for ER in figure 2D?

*ER was not detectable by IHC in any of the PDX used in the manuscript for functional studies, despite some of them (such as BCM-3277) were derived from luminal tumors. Lack of ER expression in these PDXs has been reported previously by the donor laboratories (MT Lewis, C Caldas, A Bruna and ours). As requested by the referee, we confirmed the lack of ER expression by WB and IHC in the three PDXs used for in vivo experiments, BCM-3277, STG139-M and AB521-X. The $ER^+$ MCF7 cell line was used as a positive control in the WB. ER expression was detected by IHC in the mammary glands of NSG mice as the ER antibody we used recognizes both mouse and human ER. These results have not been included in the revised manuscript to keep it focused.*

*We agree that addressing the potential regulation of RANK by ER or vice versa would be interesting. To explore this possibility we have analyzed ER expression in different $ER^+$ breast cancer cell lines where we have overexpressed RANK. As shown below, we*

*did not find any association between RANK and ER expression levels. These results are not included in the revised version of the manuscript to keep it focused.*

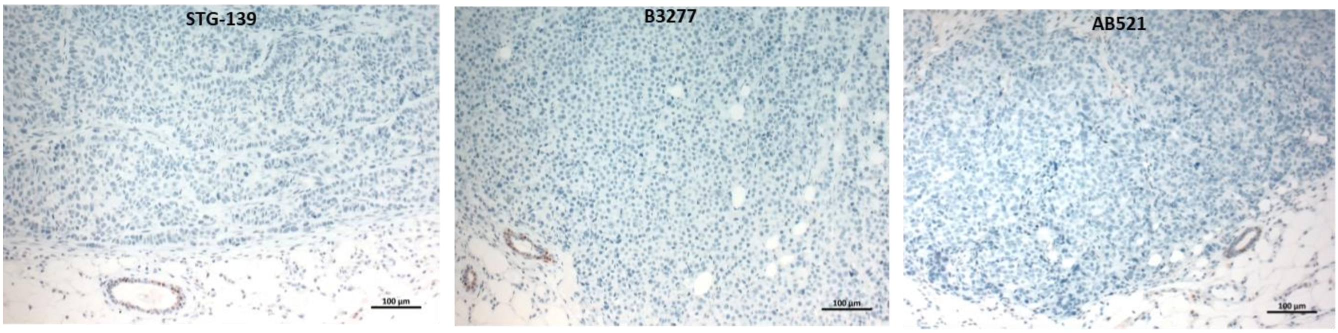


*Fig R1. ERalpha protein expression in the indicated PDXs determined by IHC.*

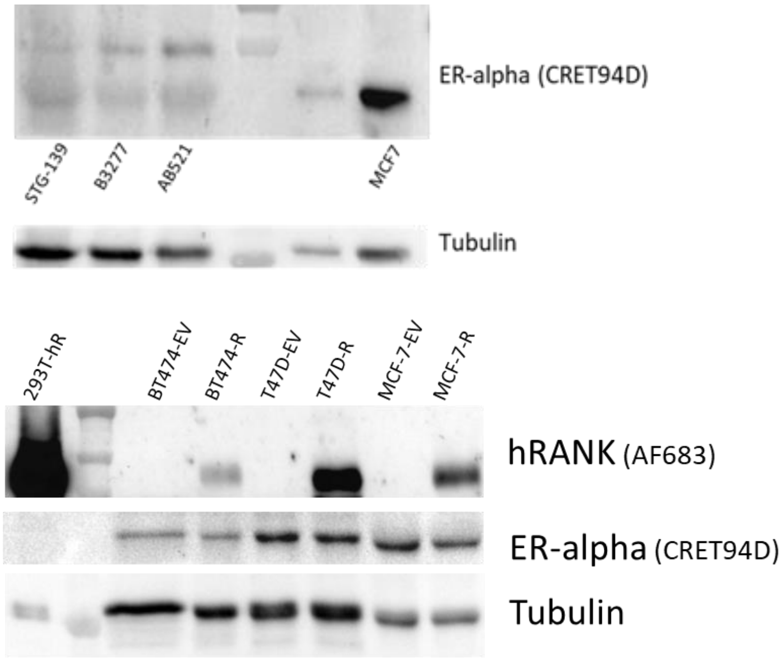


*Fig R2. hRANK and ERalpha expression determined by western blot in the indicated PDXs and hRANK-overexpressing breast cancer cell lines.*

- Why ER- BC cells respond to ovariectomy?

*The most plausible explanation is that the "response" is indirect. The drop in systemic estrogen levels has multiple effects, changes in systemic metabolism, inflammation, bone resorption and osteoporosis (due to increased RANK signaling in the bone) (Khosla et al.Trends Endocrinol. Metab. 2012, 23: 576); enhanced RANK signaling is also observed in the ER$^-$ tumors after menopause (Fig 3D). Moreover, in ER$^-$ tumors the pathways associated with RANK expression are different between pre and postmenopausal conditions (Fig 3D).*

- Postmenopausal ER+ breast cancer is treated with aromatase inhibitors. Would such inhibitors further enhance the effect of ovariectomy?

*Indeed, aromatase inhibitors (AIs) block E1 (estrone), the main source of estrogens after menopause and are efficient in postmenopausal $ER^+$ BC. Previous studies have demonstrated that the AIs letrozole and exemestane suppress tumor growth in ovariectomized mice transplanted with the $ER^+$ MCF7 breast cancer cell line (Jelovac et al. Clin Cancer Res 2004,10:7375; Nuñez et al. Clin Cancer Res 2004,10:5375).*

*As the PDX models used in this study are ER-, it is not expected that AIs will show therapeutic value. As the current manuscript is focused on the prognostic and therapeutic potential of RANK signaling in ER- BC, addressing the therapeutic benefit of AIs after ovariectomy is not in the scope of this study.*

- Does RANK-shRNA have similar effect as RANKL-inhibitors

*While shRANK will inhibit RANK signaling on the tumor cells, RANKL inhibitors will have a systemic effect, inhibiting RANK signaling not only on the tumor cells, but also in any other RANK+ cell; thus, the effects may not be similar. Ongoing research in the laboratory aims to dissect the contribution of each compartment to tumorigenesis using tissue-specific genetic cre/loxp approaches. We previously showed that RANK loss specifically in tumor cells changes the immune microenvironment (Gomez-Aleza, Nat Comm 2020) and ongoing unpublished results evidence that myeloid RANK signaling also modulates tumor growth. In this manuscript, given its translational nature, we chose to work with RANK-Fc/denosumab, as it is the current therapeutic treatment, and PDX models, given its superior clinical relevance.*

- Fig 6 should be expanded to include additional PDXs

*We have now performed the RANK-Fc treatments in ovariectomized mice bearing the BCM-3277 model where attenuation of tumor growth is also observed. Results are now included in Fig 3E and EV5C-D from the revised manuscript.*

*Figures for reviewers removed*

Other issues:

• The Abstract is a bit sloppy going back and forth about the correlation of RANK expression with poor prognosis in ER-negative BC (first) and then with poor prognosis in postmenopausal BC (Second). These two may be combined.

*We decided to keep both messages separated in the abstract. Our results support that RANK is a factor of poor prognosis and response to chemotherapy in $ER^-$ breast cancer. The chemotherapy experiments in the PDXs were performed in premenopausal conditions. On the other hand, RANK is associated with poor prognosis in postmenopausal patients from IDIBELL and NPS heterogeneous cohorts (Fig 3A and EV5A of the revised manuscript). As most of the samples in these cohorts are ER+ tumors, RANK is an independent factor of poor prognosis after menopause irrespectively of ER expression. Of course, when both conditions are met, ER- and postmenopause, the prognostic value of RANK is stronger.*

"Our results demonstrate that. RANK protein expression..." - a full stop in the middle of a sentence. One sentence is interrupted and another line starts as a new paragraph. *Thanks for noticing the mistake. It is now corrected.*

Abstract ends with no full stop.

*This is now corrected.*

•Figure 1 is not very informative; it interrupts the flow. It may be combined with Fig 3 or moved to supplemental data, and Fig 3 presented as Fig 1.

*The revised manuscript is now formatted as a Report with three main figures. We selected the most relevant findings from the previous Fig 1 to highlight the large number of samples analyzed (> 1500 from two independent collections) and the PDX models selected for functional studies.*

• Fig 1d shows distant metastasis-free survival (DMFS) of all patients is worse for RANK+ patients but not significantly. The Hazard ratio should be provided.

*A new panel including the hazard ratios is now provided for this (Fig 1E revised manuscript), but also for the other relevant findings (Fig 2D, Fig 3C). Complete information is included in Table EV1.*

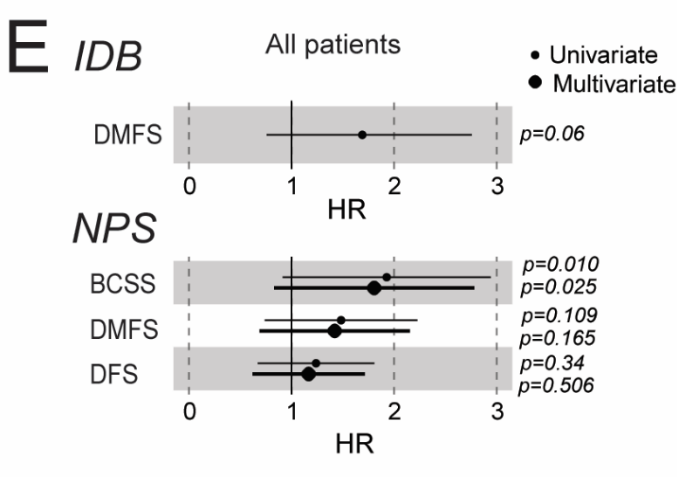


*Fig 1E*

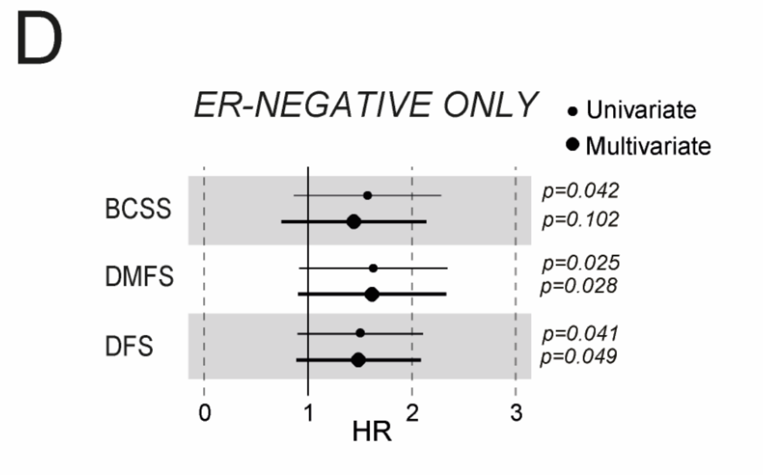


*Fig 2D*

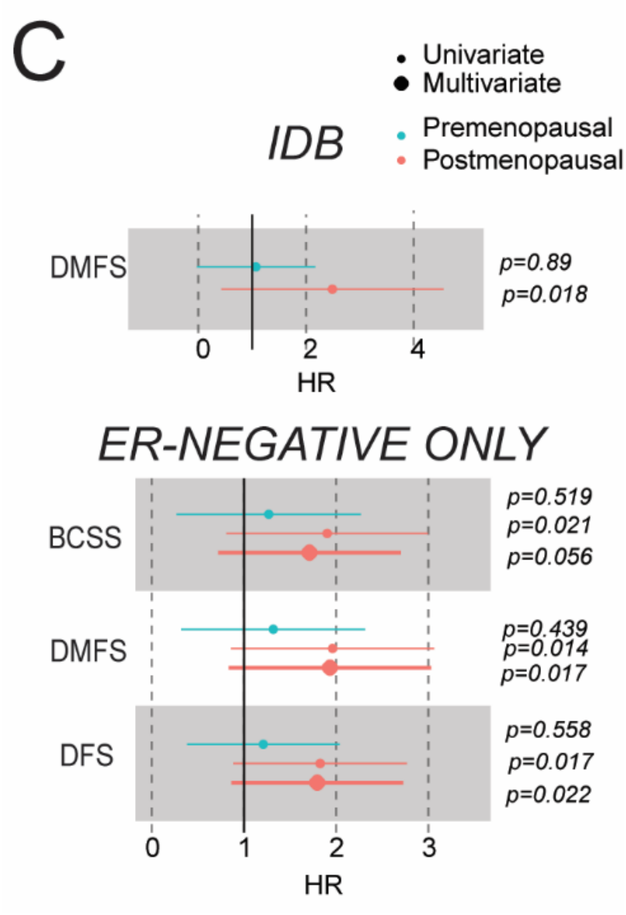


*Fig 3C*

As indicated by the authors, the worse OS of RANK+ patients (the only statistically significant cohort), and the poor DMFS may reflect the fact that RANK is a marker for TNBCs which have poor prognosis. If only TNBC patients are stratified based on RANK expression - is there any effect on clinical outcome? This analysis is shown later, in Fig 3b bottom, where high RANK is associated with a moderate but not significant worse prognosis. Overall, the differences are not great, may be subtype-related, and are not entirely clear.

*Results in Fig 1D support that RANK$^+$ patients from NPS have worse BCSS (p=0.009), which, together with the trends observed in poor DMFS in NPS and IDB cohorts, corroborate previous findings by Pfitzner GeparTrio (Blohmer et al. JAMA Oncol. 2022 8:1010).*

*In addition, Cox regression analyses in the whole NPS cohort indicate that RANK is a marker of poor BCSS (5 years) in BC, as indicated by univariate (BCSS, HR 1.928 (1.157-3.185) p=0.01) but also by multivariate (BCSS, HR 1.806 (1.076-3.028) p=0.025) analyses, independently of tumor size, grade, stage and ER status. However, not significant differences were found in DMFS or DFS (Fig 1E and Table EV1 of the revised manuscript).*

*As the referee highlighted, RANK is more frequently expressed in ER$^-$ BC, therefore we analyzed separately the ER$^+$ and ER$^-$ BC subsets of the NPS collection. In Fig EV3A of the revised manuscript (prior Fig 3) we showed that RANK expression does not associate*

*with survival in the ER$^+$ subset of NPS, whereas in the ER$^-$ subset (NPS) RANK$^+$ patients showed a trend to poorer survival (DMFS p= 0.15; BCSS p=0.08). Despite the limitations of RANK IHC in the NPS cohort (discussed in the manuscript),* ***we validated this finding in an independent collection of more than 300 ER$^-$ patients*** *(this third collection is called ER-NEGATIVE ONLY in the manuscript), where 35% of the tumors are RANK$^+$ (expected frequency for this subtype). Results from this third collection are shown in revised Fig 2C-D. Patients with RANK$^+$ tumors (113 out of 337) showed worse DMFS (p=0.023), BCSS (p=0.039), and DFS (p=0.039). Moreover, in an independent* ***fourth*** *collection of TNBC patients generated at the CNIO, patients with RANK$^+$ tumors (19 out of 56) also showed a trend for poor survival (DFS p=0.09). Thus, tumor RANK expression associated with poor prognosis in three independent ER$^-$ collections with 277 (NPS ER- subset), 337 (ER-NEGATIVE ONLY) and 56 (TNBC (CNIO)) patients. Univariate Cox regression analyses performed in the ER-NEGATIVE ONLY collection confirmed that RANK expression associated with poor BCSS (HR 1.573 p=0.042), poor DMFS (HR 1.616, p=0.025) and DFS (HR 1.503, p= 0.041). Furthermore, using multivariate analyses we found that RANK expression associated with poor DMFS (HR 1.616, p= 0.028) and DFS (HR 1.485, p=0.049), independently of tumor size, grade or stage (Fig 2C-D and Table EV1 of the revised manuscript).*

***This is solid evidence that RANK protein expression in the tumor is a biomarker of poor prognosis in ER$^-$ BC.***

Notably, a KM-Plotter analysis (https://kmplot.com) shows no significant effect of RANK expression in TN - Basal-breast cancer, based on RNA expression. When protein levels are used - high RANK expression correlates with better prognosis. Can the authors confirm and discuss the discrepancy between this site vs their IHC data?

*Several reasons may contribute to the discrepancies between this and other studies using RNA or protein data:*

1. *The levels of RANK mRNA and protein expression do not necessarily correlate, as supported by the PDX analyses. Moreover, RANK protein expression is frequently found in the stroma (Fig 1A-B), which misleads the results. Our study adds a differential analysis of RANK expression by IHC in either the tumor or the stroma using the most specific and sensitive antibody in the field.*
2. *Limitations of RANK IHC. Multiple evidence indicate that most commercial RANK antibodies are not specific (aggravated by the fact that appropriate RANK negative controls are missing in the literature). RANK is a protein with complex and fragile epitopes difficult to detect. The antibody that we used is the best recognized antibody for RANK detection in paraffin-embedded human samples. It is unclear that protein data from KM plotter discriminates tumor/stroma.*
3. *We have used a high number of ER$^-$ samples from independent collections. Previous studies including our own initial analyses included limited ER$^-$ tumors.*
4. *Quality of the survival data. It is widely accepted that the survival annotations in TCGA are not very accurate. An important value of our study is the exhaustive and detailed follow up of the large collections we used.*

• "Tumors with the highest levels of RANK mRNA expression were found in the ER- subtype. Meanwhile".

- A correlation analysis between RANK and ER should be provided.

*We have now rephrased the sentence: "RANK mRNA expression was detected in the 52 BC PDX models tested, mean levels being higher in PDX derived from $ER^-$ tumors".*

*As requested by the referee, we have analyzed ESR1 mRNA expression levels in most PDXs shown in Fig EV2A, and no correlation was found*. *Moreover, we confirmed this result using gepia, a tool to test correlation between genes in cancer* http://gepia.cancer-pku.cn/detail.php?clicktag=correlation. *No correlation between TNFRSF11A and ESR1 mRNA expression was found in a cohort of invasive breast carcinomas. Data is shown below but not included in the revised manuscript to keep the focus.*

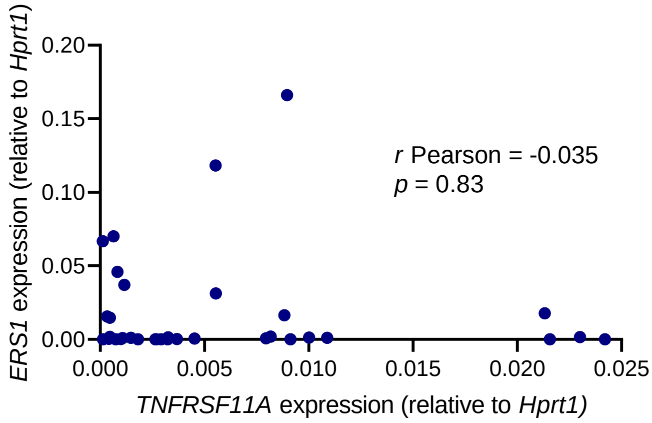


*Fig R3. TNFRSF11A and ERS1 gene expression determined by RT-PCR. It is shown the Pearson´s correlation coefficient (r) and the p value.*

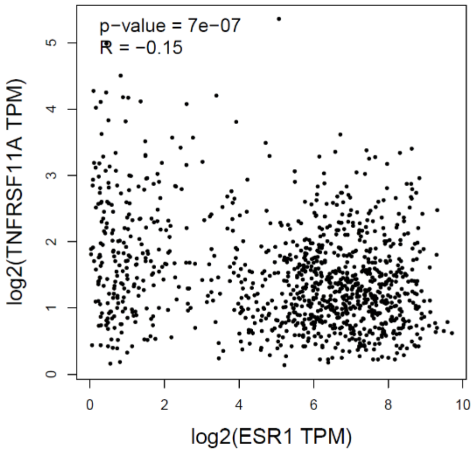


Fig R4. Correlation between TNFRSF11A and ERS1 in breast cancer using the Gene Expression Profiling Interactive Analysis (GEPIA).

• page 6 "In all cohorts, RANK expression (H > 0) was significantly associated with ER/PR negativity and TNBC subtype, but not HER2, age, tumor size or stage. In the NPS collection, RANK expression was also associated with a higher mitosis rate and grade (Fig 1d, Table S1). Shouldn't this be Fig 1C?

*The revised manuscript has been re-organized to meet the requirement of a Report.*

•Fig 2C -subtypes should be indicated.

*Table EV2 indicates the subtype of the human original tumors from which PDX models were established. However, ER expression was not detected in BCM-3277 and*

*BB3RC32, both derived from $ER^+$ tumors, in which we analyzed RANK by IHC. Subtypes are not indicated in Fig 1G and Fig EV2B for simplification.*

• Fig 4 AB521-x - P=0.005 even though the curves are very close to each other compared to BCM3277 - P=0.024. - Is this correct?

*Yes, we have compared the curves with linear regression.*

• Fig 6 shows the results in ovariectomized NSG mice. To demonstrate specificity, the authors should show side-by-side the effect of RANKL inhibition alone in normal mice as shown in Fig 4a.

*We decided to maintain the results from premenopausal and postmenopausal conditions in separate figures to facilitate the comprehension of the manuscript.*

Referee #2 (Comments on Novelty/Model System for Author):

RANK has been extensively studied in breast cancer.

*We acknowledge that since our initial discovery of RANK signaling being the main mediator of the protumorigenic role of progesterone in the mammary gland (Gonzalez-Suarez, Nature 2010), numerous studies by our laboratory and others have provided additional insights on the relevance of this pathway using cell lines, mouse models and clinical samples (most of them cited in the manuscript). However, the controversial results of the two large adjuvant clinical trials highlight the need of additional biomarkers for the selection of breast cancer patients who can benefit from denosumab.*

The conclusions are drawn from very few patient samples.

*Respectfully, we do not agree with this statement. We analyzed RANK/RANKL protein expression in more than* ***2000 breast cancer samples*** *derived from* ***four independent collections****, IDB, NPS, ER-NEGATIVE ONLY and TNBC (CNIO), which include approximately* ***700 ER$^-$*** *tumors. 35% of the ER- samples in the IDB, ER-NEGATIVE ONLY and TNBC (CNIO) cohorts express RANK (similar frequency as shown in previous studies (Palafox et al. Cancer Res. 2012, 72:2879; Pfitzner et al. Breast Cancer Res. Treat. 2014, 145:307).*

*The analyses from three independent cohorts (NPS -277 tumors-, ER-NEGATIVE ONLY -377 tumors- and TNBC (CNIO) -56 tumors-) strongly support that* ***RANK is a marker of poor prognosis in ER$^-$ BC****. Cox regression analyses in the ER-NEGATIVE ONLY cohort reinforce this conclusion.*

*The importance of RANK as a marker of poor survival in postmenopausal BC is proven using data from three independent collections (IDB -117-, NPS -618- and ER-NEGATIVE ONLY -189- tumors). Cox regression analyses supports that* ***RANK is an independent poor survival marker in postmenopausal ER$^-$ BC.***

Referee #2 (Remarks for Author):

In this manuscript, Ciscar et al. present evidence that RANK is a poor prognosis and a potential target in ER-negative breast cancer. Denosumab (DNS) a RANK ligand (RANKL) inhibitor has been used clinically in breast cancer (BC) for decades, in patients who have bone metastasis to prevent further loss of bone mass, most often associated with ER-positive breast cancers. DNS is also extensively used in patients with osteoporosis to prevent further bone density loss. Both RANK and the receptor of RANKL activity have been heavily published in BC, including by the authors. The authors present data from two independent TMA collections for all BC subtypes from the IDIBELL (IDB) and the Nottingham series (NPS) s and a subset of the NPS which were part of the METRABIC study.

The IHC from these data was not impressive for RANK protein expression. The number of RANK-positive was highest in the NPS than IDB study with The result for TNBC in both IRB and NPS subtypes was correlating with the highest expression of RANK. RANK expression has been shown to be a prognostic and predictive marker in breast cancer subtypes.

*Beyond the heterogeneous IDB (n= 404) and NPS (n= 1895) collections, we provide data of two additional collections containing exclusively ER$^-$ samples: ER-NEGATIVE ONLY, which includes 359 ER$^-$ samples and TNBC (CNIO), with 66 samples, to confirm findings*

*in ER$^-$ BC. Table EV1 includes detailed results from all collections, identified by colored tabs.*

*Regarding RANK IHC, the frequency and H-score for RANK positivity in the IDB, ER$^-$ NEGATIVE ONLY and TNBC (CNIO) collections were similar to those reported in previous studies using the N1H8 antibody for human RANK, the best and more specific antibody known for RANK in paraffin-embedded samples**: 35% of ER$^-$ tumors and 18% of ER$^+$ tumors are positive for RANK expression**. In the NPS collection (not in IDB) the frequency and intensity of RANK$^+$ tumors were lower than previously reported (Fig 1A). We always use several positive and negative controls for RANK staining, based on human samples and our well characterized PDXs, where we have demonstrated RANK expression by RNA/protein and, more importantly, activation upon RANKL stimulation (representative pictures of the positive/negative controls are included in Sanz-Moreno et al., BCR, 2021). Since we are using TMAs generated decades ago, we are probably underestimating the RANK$^+$ tumors.*

*Previous studies (Pfitzner et al., Breast Cancer Res Treat, 2014) concluded that RANK associated with poor survival in heterogeneous cohorts, but it was not an independent marker, as this was associated with its prevalence in TNBC, as the referee points out. Importantly, in this manuscript we demonstrated that RANK associates with poor survival specifically in ER$^-$ BC. These conclusions are solid, as they were validated in 3 independent cohorts (RANK being an independent marker of poor survival), but additional studies will be required to draw final conclusions in the ER$^+$ subtypes.*

*Moreover, based on Cox multivariate analyses our study is the first to show that:*

1. *In BC, RANK positivity is a marker of poor BCSS (HR=1.806 (1.076-3.028) p=0.025), independently of tumor size, grade, stage and ER status (NPS).*
2. *In ER$^-$ BC, RANK positivity is a marker of poor DMFS (HR=1.616, p= 0.028) and poor DFS (HR=1.485, p=0.049), independently of tumor size, grade or stage.*
3. *In postmenopausal BC, RANK positivity is a marker of poor DMFS (HR=2.046, p= 0.025) and poor BCSS (HR=2.313, p=0.013), independently of tumor size, grade, stage and ER status, in contrast to ER expression, which was not associated with any survival parameter in this collection.*
4. *In postmenopausal ER$^-$ BC, RANK positivity is a marker of poor DMFS (HR= 1.933, p= 0.017) and poor DFS (HR=1.795, p=0.022), independently of tumor size, grade, stage and ER status.*

Functional, validation for RANK was done by using several PDX models, the authors show that RANK is mostly restricted to ER-negative PDX tumors. Exposure to hRANKL activated the TNF/NFKB signaling pathway in three PDX models in vitro ER-negative. And In vivo PDX ER-negative tumor treated with RANK inhibitor (RANK-Fc) alone or in combination with docetaxel inhibition showed moderate effects on tumor growth. Nonetheless, combination with docetaxel decreases tumor growth.

Comparisons using expression profiling with generated GSEA pathways utilizing: 1) METABRIC, PDX derived tumors inhibited for RANK activity and clinical trials for early breast cancer using denosumab concluded the RANK signature prevails in ER-negative BC above the ER-positive BC.

*GSEA analyses in METABRIC evidence that the biology of RANK is distinct between ER$^+$ BC, where it negatively associates with proliferation (in agreement with Benitez et al.), and ER$^-$ BC, where it associates with tumor cell metabolism. RNAseq on PDX ER$^-$*

*tumors treated with RANKL and RANK-Fc provides functional evidence that modulation of RANK signaling impacts several biological processes, mainly metabolic pathways. Together, our data provide strong evidence of the prognostic and therapeutic value of RANK in $ER^-$ BC. Regarding $ER^+$ BC, analyses in additional cohorts and functional studies in $ER^+$ models will be required to draw conclusions in this subtype.*

The GSEA pathways /data sets/ survival outcome(s), along with premenopausal and postmenopausal status were integrated to further provide evidence that RANK is most prominently predictive in premeopsaul ER-negative BC. Suggesting that clinical work should target RANK as a precision target in premenopausal ER-negative breast cancer a very important aspect to meet clinic needs.

*Our data in 3 independent collections, IDB, NPS and ER-NEGATIVE ONLY, supports that RANK expression is a marker of poor prognosis in BC and $ER^-$ BC in* ***postmenopausal women****.*

*GSEA analyses in METABRIC also demonstrate that RANK protein expression strongly associates with NFKB activation in postmenopausal BC but not in premenopausal BC, which may be indicative of an enhanced RANK signaling and/or inflammation in postmenopausal tumors.*

In general: This is an extensive study that provide evidence to convince us that RANK is important in ER-negative BC from a leader in the field. Yet several areas of concern as to the virtuosity of the study as to how widely to define ER-negative breast cancer subtypes, that is within such a category you also may harbor other subtypes, e.g., TNBC, HER2, BRCA etc... The nomenclature used to draw conclusion between ER-negative and ER-positive BC cause concerns due to having analysis on limited sample size, that de-arms datas statistically insignificant. In the present form the manuscript is not acceptable and or will require major revisions.

*We agree that $ER^-$ BC is heterogeneous and can be further sub-classified according to HER2 expression, BRCA1 mutations, and many other parameters. The value of our study is that it provides an additional parameter, RANK expression, independently associated with prognosis in $ER^-$ BC. More importantly, we provide evidence that women, particularly postmenopausal women with $RANK^+$ $ER^-$ BC, may benefit from denosumab treatments.*

*To our knowledge this is the first study that concludes that RANK is a biomarker of poor prognosis, based on results from more than* ***700 $ER^-$ BC*** *from three independent cohorts:*

*- ER- subset of NPS: 278 ER- patients, 36 being RANK+.*

*- ER-NEGATIVE ONLY collection: 396 ER- patients, 113 being RANK+.*

*- TNBC CNIO collection: 66 patients, 19 being RANK+.*

***This is solid evidence that RANK protein expression in the tumor is a biomarker of poor prognosis in $ER^-$ BC.*** *We agree with the referee that additional analyses are required to draw any conclusions about the predictive/prognostic value of RANK in $ER^+$ BC. See below the additional analyses we have performed according to HER2, BRCA1 and basal markers.*

Major/Minor issues:

1) The authors are well verse and know that BC subtypes is a heterogenous disease. ER+, PR+, ER+/PR+ HER2, Luminal A, Luminal B , Basal-like, BRCA, TNBC etc...

2) ER-negative can be reflective of several subtype for example, PR+ or HER2 or a TNBC they are all ER-negative so is RANK. As example of this in the Nottingham Series were histological grade is provide to be significant for RANK expression the data reflect only 61 patient samples out of 1,054.
3) Similarly with the "vascular invasion" significance is based on 4 positive patients to make ana argument for RANK expression to be important paler in vascular invasion.

*We do not draw solid conclusions about associations of RANK expression with histological grade or vascular invasion, as they are based on few cases and observed in only one of the cohorts. These results are reported but would need to be validated in additional cohorts. The conclusions of the manuscript are based on results derived from at least two/three independent collections.*

*We are aware of the heterogeneity of breast cancer and implications for prognosis. For this reason and because RANK was more frequently found in $ER^-$ tumors, which have poor prognosis, we analyzed $ER^+$/$ER^-$ tumors separately (see Fig 2 and Fig EV3 of the revised manuscript). Gene expression data is not available, therefore, it is not possible to classify the samples by molecular subtype.*

*Despite we did not find any association between RANK and HER2, we have now performed the survival analyses separately in $HER2^+$ BC as requested by the referee, but also in $ER^-$ BRCA1-mutated tumors and $ER^-$ tumors with basal markers.*

*In the NPS cohort (all patients), RANK positivity associated with poor BCSS, DMFS and DFS in HER2+ (7 RANK+ out of 125) tumors. The association of RANK with poor survival in HER2+ tumors were also observed in postmenopausal but not in premenopausal tumors. However, this conclusion is based on only 7 $RANK^+$ $HER2^+$ tumors. These analyses have been included in Table EV1.*

| NOTTINGHAM PRIMARY SERIES (HER2+) | | | | |
|---|---|---|---|---|
| **Parameters** | **Total N** | **N of events** | **Time** | ***p*-value (Log Rank (Mantel-Cox))** |
| **BCSS** | 125 | 38 | 60 months | 0.001 |
| No RANK expression | 118 | 33 | | |
| RANK expression | 7 | 5 | | |
| **BCSS** | 108 | 54 | 180 months | 0.002 |
| No RANK expression | 102 | 49 | | |
| RANK expression | 6 | 5 | | |
| **DMFS** | 124 | 47 | 60 months | 0.009 |
| No RANK expression | 117 | 42 | | |
| RANK expression | 7 | 5 | | |
| **DMFS** | 124 | 60 | 240 months | 0.025 |
| No RANK expression | 117 | 55 | | |
| RANK expression | 7 | 5 | | |
| **DFS** | 125 | 54 | 60 months | 0.05 |
| No RANK expression | 118 | 49 | | |
| RANK expression | 7 | 5 | | |
| **DFS** | 125 | 68 | 240 months | 0.106 |
| No RANK expression | 118 | 63 | | |
| RANK expression | 7 | 5 | | |

| NOTTINGHAM PRIMARY SERIES (Premenopausal HER2+) | | | | |
|---|---|---|---|---|
| **Parameters** | **Total N** | **N of events** | **Time** | ***p*-value (Log Rank (Mantel-Cox))** |
| **BCSS** | 55 | 16 | 60 months | no |
| No RANK expression | 55 | 16 | | |
| RANK expression | | | | |
| **BCSS** | 46 | 28 | 180 months | no |
| No RANK expression | 46 | 28 | | |
| RANK expression | | | | |
| **DMFS** | 56 | 22 | 60 months | no |
| No RANK expression | 56 | 22 | | |
| RANK expression | | | | |
| **DMFS** | 56 | 31 | 240 months | no |
| No RANK expression | 56 | 31 | | |
| RANK expression | | | | |
| **DFS** | 56 | 26 | 60 months | no |
| No RANK expression | 56 | 26 | | |
| RANK expression | | | | |
| **DFS** | 56 | 34 | 240 months | no |
| No RANK expression | 56 | 34 | | |
| RANK expression | | | | |

| NOTTINGHAM PRIMARY SERIES (Postmenopausal HER2+) | | | | |
|---|---|---|---|---|
| **Parameters** | **Total N** | **N of events** | **Time** | ***p*-value (Log Rank (Mantel-Cox))** |
| **BCSS** | 69 | 22 | 60 months | 0.006 |
| No RANK expression | 62 | 17 | | |
| RANK expression | 7 | 5 | | |
| **BCSS** | 61 | 26 | 180 months | 0.004 |
| No RANK expression | 55 | 21 | | |
| RANK expression | 6 | 5 | | |
| **DMFS** | 68 | 25 | 60 months | 0.019 |
| No RANK expression | 61 | 20 | | |
| RANK expression | 7 | 5 | | |
| **DMFS** | 68 | 29 | 240 months | 0.036 |
| No RANK expression | 61 | 24 | | |
| RANK expression | 7 | 5 | | |
| **DFS** | 68 | 28 | 60 months | 0.059 |
| No RANK expression | 61 | 23 | | |
| RANK expression | 7 | 5 | | |
| **DFS** | 471 | 187 | 240 months | 0.284 |
| No RANK expression | 449 | 180 | | |
| RANK expression | 22 | 7 | | |

*In the ER-NEGATIVE ONLY collection, a total of 69 patients were HER2$^+$, defined by IHC/FISH, and 19 of them RANK$^+$. RANK expression associated with poor DFS at 5 and 10 years. The association of RANK with poor DMFS in HER2$^+$ patients was observed in postmenopausal (11 RANK$^+$ out of 41) but not in premenopausal (8 RANK$^+$ out of 28) patients. However, it is important to consider that these HER2$^+$ patients received chemotherapy alone or in combination with HER2-targeted therapies, making it more difficult to draw conclusions. We previously reported that RANK expression was not associated with response to anti-HER2 therapies. However, RANK expression increased after anti-HER2 treatment and may be involved in resistance to anti-HER2 therapies (Sanz-Moreno et al. BCR, 2021).*

| ER-NEGATIVE ONLY (HER2+) | | | | |
|---|---|---|---|---|
| **Parameters** | **Total N** | **N of events** | **Time** | ***p*-value (Log Rank (Mantel-Cox))** |
| **BCSS** | 69 | 20 | 60 months | 0.428 |
| No RANK expression | 50 | 13 | | |
| RANK expression | 19 | 7 | | |
| **BCSS** | 69 | 22 | 120 months | 0.135 |
| No RANK expression | 50 | 13 | | |
| RANK expression | 19 | 9 | | |
| **DMFS** | 69 | 24 | 60 months | 0.05 |
| No RANK expression | 50 | 14 | | |
| RANK expression | 19 | 10 | | |
| **DMFS** | 69 | 24 | 120 months | 0.05 |
| No RANK expression | 50 | 14 | | |
| RANK expression | 19 | 10 | | |
| **DFS** | 69 | 27 | 60 months | 0.033 |
| No RANK expression | 50 | 16 | | |
| RANK expression | 19 | 11 | | |
| **DFS** | 69 | 28 | 120 months | 0.044 |
| No RANK expression | 50 | 17 | | |
| RANK expression | 19 | 11 | | |

| ER-NEGATIVE ONLY (Premenopausal HER2+) | | | | |
|---|---|---|---|---|
| **Parameters** | **Total N** | **N of events** | **Time** | ***p*-value (Log Rank (Mantel-Cox))** |
| **BCSS** | 28 | 10 | 60 months | 0.952 |
| No RANK expression | 20 | 7 | | |
| RANK expression | 8 | 3 | | |
| **BCSS** | 28 | 10 | 120 months | 0.952 |
| No RANK expression | 20 | 7 | | |
| RANK expression | 8 | 3 | | |
| **DMFS** | 28 | 12 | 60 months | 0.805 |
| No RANK expression | 20 | 8 | | |
| RANK expression | 8 | 4 | | |
| **DMFS** | 28 | 12 | 120 months | 0.805 |
| No RANK expression | 20 | 8 | | |
| RANK expression | 8 | 4 | | |
| **DFS** | 28 | 13 | 60 months | 0.289 |
| No RANK expression | 20 | 8 | | |
| RANK expression | 8 | 5 | | |
| **DFS** | 28 | 13 | 120 months | 0.289 |
| No RANK expression | 20 | 8 | | |
| RANK expression | 8 | 5 | | |

| ER-NEGATIVE ONLY (Postmenopausal HER2+) | | | | |
|---|---|---|---|---|
| **Parameters** | **Total N** | **N of events** | **Time** | ***p*-value (Log Rank (Mantel-Cox))** |
| **BCSS** | 41 | 10 | 60 months | 0.26 |
| No RANK expression | 30 | 6 | | |
| RANK expression | 11 | 4 | | |
| **BCSS** | 41 | 12 | 120 months | 0.031 |
| No RANK expression | 30 | 6 | | |
| RANK expression | 11 | 6 | | |
| **DMFS** | 41 | 12 | 60 months | 0.018 |
| No RANK expression | 30 | 6 | | |
| RANK expression | 11 | 6 | | |
| **DMFS** | 41 | 12 | 120 months | 0.018 |
| No RANK expression | 30 | 6 | | |
| RANK expression | 11 | 6 | | |
| **DFS** | 41 | 14 | 60 months | 0.064 |
| No RANK expression | 30 | 8 | | |
| RANK expression | 11 | 6 | | |
| **DFS** | 41 | 15 | 120 months | 0.087 |
| No RANK expression | 30 | 9 | | |
| RANK expression | 11 | 6 | | |

*In the ER-NEGATIVE ONLY collection we also analyzed the prognostic value of RANK in the "Basal" tumors (CK5$^+$); there are 44 RANK$^+$ tumors out of 140 (24/41 premenopausal and 20/75 postmenopausal). No significant associations were found, although there was a trend with poor DFS at 5 and 10 years in postmenopausal patients (p=0.06).*

| ER-NEGATIVE ONLY (Basal) | | | | |
|---|---|---|---|---|
| **Parameters** | **Total N** | **N of events** | **Time** | ***p*-value (Log Rank (Mantel-Cox))** |
| **BCSS** | 140 | 25 | 60 months | 0.88 |
| No RANK expression | 96 | 17 | | |
| RANK expression | 44 | 8 | | |
| **BCSS** | 140 | 30 | 120 months | 0.461 |
| No RANK expression | 96 | 19 | | |
| RANK expression | 44 | 11 | | |
| **DMFS** | 140 | 27 | 60 months | 0.709 |
| No RANK expression | 96 | 18 | | |
| RANK expression | 44 | 9 | | |
| **DMFS** | 140 | 29 | 120 months | 0.601 |
| No RANK expression | 96 | 19 | | |
| RANK expression | 44 | 10 | | |
| **DFS** | 140 | 32 | 60 months | 0.587 |
| No RANK expression | 96 | 21 | | |
| RANK expression | 44 | 11 | | |
| **DFS** | 140 | 37 | 120 months | 0.74 |
| No RANK expression | 96 | 25 | | |
| RANK expression | 44 | 12 | | |

| ER-NEGATIVE ONLY (Premenopausal basal) | | | | |
|---|---|---|---|---|
| **Parameters** | **Total N** | **N of events** | **Time** | ***p*-value (Log Rank (Mantel-Cox))** |
| **BCSS** | 65 | 9 | 60 months | 0.865 |
| No RANK expression | 41 | 6 | | |
| RANK expression | 24 | 3 | | |
| **BCSS** | 65 | 9 | 120 months | 0.865 |
| No RANK expression | 41 | 6 | | |
| RANK expression | 24 | 3 | | |
| **DMFS** | 65 | 7 | 60 months | 0.666 |
| No RANK expression | 41 | 5 | | |
| RANK expression | 24 | 2 | | |
| **DMFS** | 65 | 7 | 120 months | 0.666 |
| No RANK expression | 41 | 5 | | |
| RANK expression | 24 | 2 | | |
| **DFS** | 65 | 9 | 60 months | 0.364 |
| No RANK expression | 41 | 7 | | |
| RANK expression | 24 | 2 | | |
| **DFS** | 65 | 11 | 120 months | 0.207 |
| No RANK expression | 41 | 9 | | |
| RANK expression | 24 | 2 | | |

| ER-NEGATIVE ONLY (Postmenopausal basal) | | | | |
|---|---|---|---|---|
| **Parameters** | **Total N** | **N of events** | **Time** | ***p*-value (Log Rank (Mantel-Cox))** |
| **BCSS** | 75 | 16 | 60 months | 0.561 |
| No RANK expression | 55 | 11 | | |
| RANK expression | 20 | 5 | | |
| **BCSS** | 75 | 21 | 120 months | 0.169 |
| No RANK expression | 55 | 13 | | |
| RANK expression | 20 | 8 | | |
| **DMFS** | 75 | 20 | 60 months | 0.257 |
| No RANK expression | 55 | 13 | | |
| RANK expression | 20 | 7 | | |
| **DMFS** | 75 | 22 | 120 months | 0.186 |
| No RANK expression | 55 | 14 | | |
| RANK expression | 20 | 8 | | |
| **DFS** | 75 | 23 | 60 months | 0.066 |
| No RANK expression | 55 | 14 | | |
| RANK expression | 20 | 9 | | |
| **DFS** | 75 | 26 | 120 months | 0.062 |
| No RANK expression | 55 | 16 | | |
| RANK expression | 20 | 10 | | |

*Finally, in the ER-NEGATIVE ONLY collection, 24 tumors were BRCA1 mutant, and 11 of them RANK$^{+}$. In BRCA1 mutants RANK expression was clearly associated with poor DFS (p=0.006), while a trend with poor BCSS (p=0.106) and poor DMFS (p=0.115) was observed.*

| ER-NEGATIVE ONLY (BRCA1 WT- all patients) | | | | |
|---|---|---|---|---|
| **Parameters** | **Total N** | **N of events** | **Time** | ***p*-value (Log Rank (Mantel-Cox))** |
| **BCSS** | 239 | 50 | 60 months | 0.714 |
| No RANK expression | 163 | 33 | | |
| RANK expression | 76 | 17 | | |
| **BCSS** | 239 | 59 | 120 months | 0.199 |
| No RANK expression | 163 | 36 | | |
| RANK expression | 76 | 23 | | |
| **DMFS** | 239 | 58 | 60 months | 0.376 |
| No RANK expression | 163 | 37 | | |
| RANK expression | 76 | 21 | | |
| **DMFS** | 239 | 64 | 120 months | 0.24 |
| No RANK expression | 163 | 40 | | |
| RANK expression | 76 | 24 | | |
| **DFS** | 239 | 66 | 60 months | 0.29 |
| No RANK expression | 163 | 42 | | |
| RANK expression | 76 | 24 | | |
| **DFS** | 239 | 73 | 120 months | 0.321 |
| No RANK expression | 163 | 47 | | |
| RANK expression | 76 | 26 | | |

| ER-NEGATIVE ONLY (BRCA1-mutated all patients) | | | | |
|---|---|---|---|---|
| **Parameters** | **Total N** | **N of events** | **Time** | ***p*-value (Log Rank (Mantel-Cox))** |
| **BCSS** | 24 | 2 | 60 months | 0.106 |
| No RANK expression | 13 | 0 | | |
| RANK expression | 11 | 2 | | |
| **BCSS** | 24 | 2 | 120 months | 0.106 |
| No RANK expression | 13 | 0 | | |
| RANK expression | 11 | 2 | | |
| **DMFS** | 24 | 2 | 60 months | 0.115 |
| No RANK expression | 13 | 0 | | |
| RANK expression | 11 | 2 | | |
| **DMFS** | 24 | 2 | 120 months | 0.115 |
| No RANK expression | 13 | 0 | | |
| RANK expression | 11 | 2 | | |
| **DFS** | 24 | 5 | 60 months | 0.006 |
| No RANK expression | 13 | 0 | | |
| RANK expression | 11 | 5 | | |
| **DFS** | 24 | 5 | 120 months | 0.006 |
| No RANK expression | 13 | 0 | | |
| RANK expression | 11 | 5 | | |

*However, as the referee highlights, sample size in these comparisons is small and validations in independent cohorts are required to get solid conclusions. Results are included in Table EV1 but not in the main figures.*

4) Additionally, BCSS and DMFS significance is driving by few sample number the ER-negative Nottingham Series 8 and 7.

*Total N is the total number of samples included in each group and N of events is the number of patients that reach the event, for example, patients that develop distant metastasis in the case of DMFS. In the NPS $ER^-$ subset, there are 36 $RANK^+$ out of 277, and 13 $RANK^+$ patients reach the BCSS (orange tabs in Table EV1, Fig EV3A). Importantly, the conclusion that RANK expression associates with poor survival in the ER-negative BC, is confirmed in an independent collection: ER-NEGATIVE ONLY cohort (**113 $RANK^+$ out of 337**) (Fig 2C, green tabs Table EV1).*

5) The author never reflect the true number in the text they are significance but in very few samples.

*To facilitate the reading and to adjust to the journal guidelines we decided not to cite the numbers in the text, but they are shown in the figures and the Tables.*

6) The menopausal status for RANK expression the Nottingham Series for ER+ BC was significant with only 24 driving the postmenopausal significance. Yet the ER-negative cohort BC postmenopausal was not statistically significance. Is not the argument that RANK is important in ER-negative postmenopausal women? Please clarify.

*RANK expression was not associated with survival in $ER^+$ BC (Fig EV3A), so we did not analyze associations with menopause in the $ER^+$ samples. However, additional studies on ER+ tumors will be required to draw conclusions, given the limitation of RANK detection in the NPS cohort.*

*Data in Fig 3A (IDB) show that RANK expression associated with poor DMFS in postmenopausal patients (33 $RANK^+$ out of 117 (p=0.01)) (pink tabs in Table EV1). This was validated in the NPS (35 $RANK^+$ out of 618) (Fig EV5A and orange tabs in Table EV1).*

*In the NPS $ER^-$ subset, RANK expression associated with poor DMFS (p= 0.009) and BCSS (p=0.004) in postmenopausal patients (13 $RANK^+$ out of 136), while no association was found in premenopausal patients (23 $RANK^+$ out of 132): DMFS (p=0.85), BCSS (0.75) (Fig EV5A, orange tabs in Table EV1).*

*These findings were validated in an independent collection containing only $ER^-$ tumors, where the frequency of RANK expression was 33%, in line with previous reports (ER-NEGATIVE ONLY collection) (Fig 3B, green tabs in Table EV1). In this collection, RANK expression associated with poor DMFS (p=0.01) and BCSS (p= 0.02) in postmenopausal patients (56 $RANK^+$ 133 RANK-). In contrast, no association with survival was found in premenopausal patients (57 $RANK^+$ and 91 RANK-; DMFS p=0.43; BCSS p=0.51).*

*We included a new panel with the Cox regression analyses (Fig 3C) to highlight these findings.*

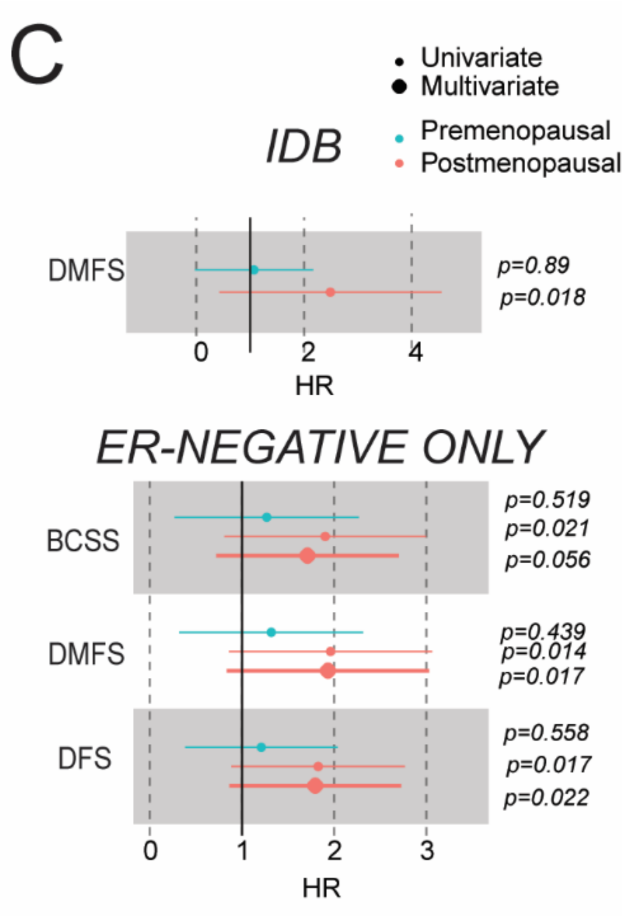


*Fig 3C*

7) On occasion this reviewer was not sure if the authors were speaking describing correct clinical data when this reviewer was looking at in supplemental tables. A possible suggestion a "red highlight" in the excel table sheet would facilitate finds the discussed results.

*Thanks for the suggestion, to facilitate the reading and comprehension, we have improved Table EV1: highlighting in red the p value of the significant associations, which are discussed in the text, including each collection in a different tab with different colors, and improving the index in the first page.*

8) Figure 1: IHC for stroma vs tumor staining for RANK was not impressive as the staining looks like it is mostly stroma and no tumor. One sample of IHC does not represent the IDB and or NPS study to draw these conclusions. None of these results are in ER negative subtypes. SFS1A/B/C has no statistical significance shown.

*In the revised manuscript we have included new pictures of positive staining for RANK/RANKL (Fig 1B). These pictures show representative positive samples to prove the specificity of the staining. We have included pictures from ER-negative tumors in Fig 1B, as requested.*

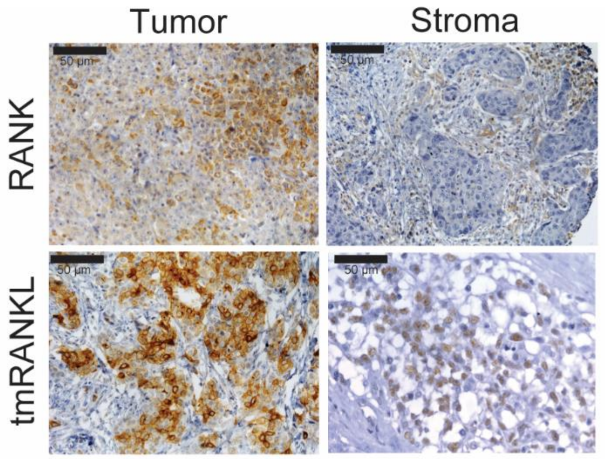


*Fig 1B*

*The IHC in the four collections have been analyzed and scored (H-scores) by an experienced researcher and three independent pathologists, blind for the clinicopathological parameters (CP) and survival annotations.*

*H-scores for tumor RANK and RANKL in the three collections are shown in Fig EV1B (previous S1a-c). We do not intend to "compare" these variables, so no statistical analyses are performed.*

9) Figure 2: The authors state that there is no "functionality of RANK in human BC..." there are at minimum 256 published articles on RANK in BC, and more than one has done functionality studies of RANK. Fig2a qPCR is of poor resolution.
*Most functional studies rely on breast cancer cell lines or mouse models. To our knowledge, our study is the first to provide functional studies on $RANK^{+}$ BC samples derived from patients (PDXs). We have now rephrased to clarify: "Despite encouraging results in BC mouse models and cell lines (Yoldi et al. Cancer Res. 2016, 76:5857), the functional relevance of RANK signaling in clinical breast cancer remains poorly studied". Resolution of Fig EV2A (prior S2a) has been improved.*

10) Figure 2D why was no RANK western run of the PDX tumors? because IHC on the PDX not impressive and is the BCM3277 least impressive by IHC, yet is very responsive to hRANKL activation of TNF/NFKB signaling. SFS2B RANK in the STG139M is low vs that of BCM-3277 is high yet IHC is the reverse.
*We and others have shown that cells with low levels of RANK, detected by IHC, can be responsive to RANKL, while others with similar levels are not (see WB in Palafox et al Cancer Res 2012, Sanz-Moreno et al 2021). For this reason, selection the PDX models for the in vivo experiments was based on RANKL responsiveness (downstream RANK targets and NFKB activation) and not only on RANK expression (Fig 1 and Fig EV2).*
*As requested by the referee, we have now analyzed RANK protein expression by WB using the AF683 antibody from R&D. RANK protein expression is detected in the AB521-X PDX, which shows the highest RANK expression by IHC. This is in accordance with our experience indicating that IHC using the N1H8 is the most sensitive and specific manner to detect RANK.*

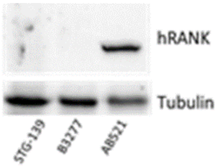


Fig R5. hRANK protein expression in the indicated PDX models using the AF683 antibody (R&D Systems), determined by western blot.

11) If BCM3277 was original an ER positive as stated then become a ER negative how do the authors know if PR or HER2 is not there as well? Or if it's truly TNBC? Same goes for the other PDX models used.

*BCM-3277 was provided by Mile T Lewis (BCM). They have characterized ER, PR, HER2 expression in the human sample of origin, as well as in the PDX, confirming that the PDX was a TNBC. In addition, PAM50 analyses confirmed the subtype was basal-like subtype.*

*Both STG139-M and AB521-X were derived from TNBC tumors; and PDXs from these tumors remained TN.*

12) Rationale not provide as to why RNA seq was done on the PDX model with exposure to RANKL for one month.

*We have now explained in the text that the goal was to confirm the impact of constitutive activation of RANK signaling in tumor biology.*

13) Fig3E is followed by "Tumor stage independently associated with three survival parameters analyzed.." refer data to Table S1. Which specific comparisons where done did the authors use the COX NPS vs CP NPS tabs? Please clarify.

*Table EV1 includes a tab (CP NPS) showing the association between RANK and clinicopathologic parameters. Survival analyses (BCSS, DFS and DMFS) were performed using the log Rank test. In some cases, Cox regression analyses (univariate and multivariate) were performed. A detailed index is shown in the first tab of Table EV1.*

*We concluded that patients with $RANK^{+}$ tumors are associated with poor survival. Multivariate Cox regression analysis show that RANK is an independent biomarker of poor BCSS, DMFS and DFS at 10 years of follow-up, independently of the tumor size, stage or grade. With this sentence we wanted to highlight that only tumor stage, but not tumor grade or size, was an independent biomarker of poor prognosis (tab Cox ER-NEGATIVE ONLY). RANK expression is a stronger biomarker of prognosis than tumor grade or size in $ER^{-}$ BC. Results from the Cox regression analyses are now included in the main figures (Figs 1E, 2D, 3C).*

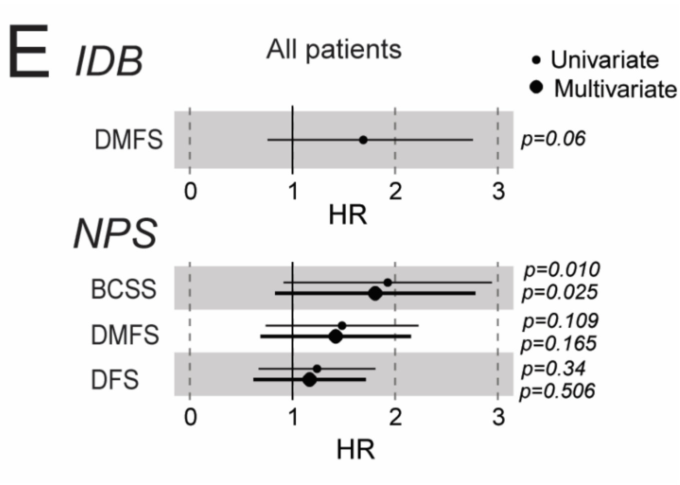
E
IDB
All patients
Univariate
Multivariate
DMFS
p=0.06
0
1
2
3
HR
NPS
BCSS
DMFS
DFS
p=0.010
p=0.025
p=0.109
p=0.165
p=0.34
p=0.506
0
1
2
3
HR

*Fig 1E*

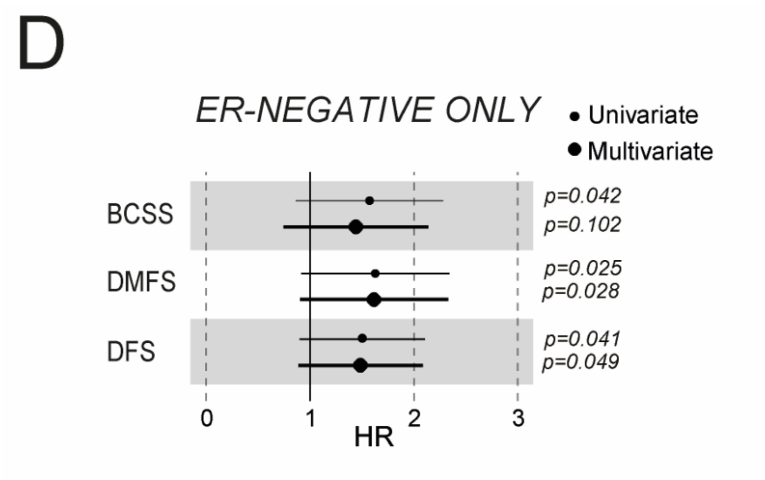
D
ER-NEGATIVE ONLY
Univariate
Multivariate
BCSS
DMFS
DFS
p=0.042
p=0.102
p=0.025
p=0.028
p=0.041
p=0.049
0
1
2
3
HR

*Fig 2D*

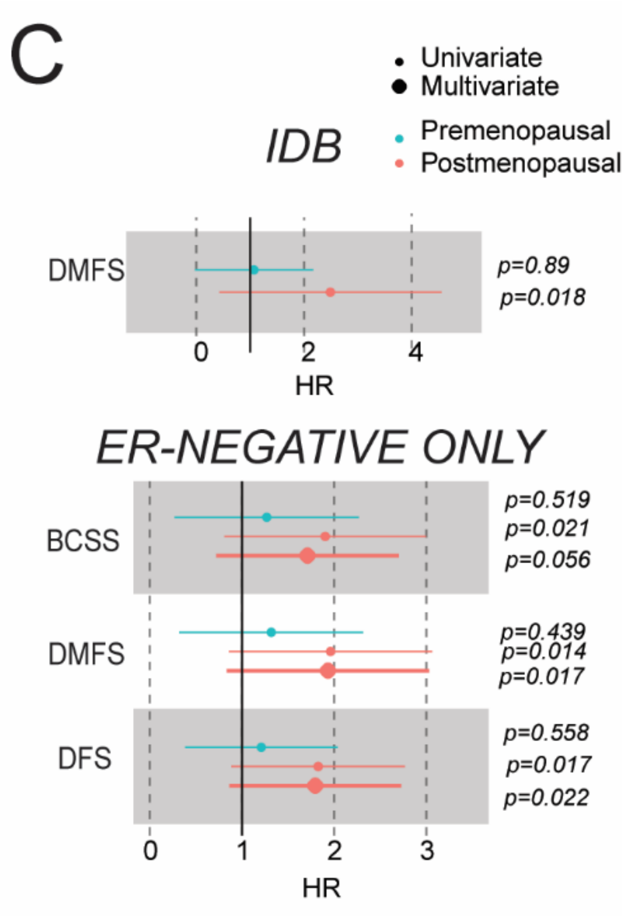


*Fig 3C*

14) Fig4. In Fig 1 IHC show that PDX AB521-X has more RANK that STG139-M, both responded to hRANKL yest these same PDX is never treated with DNS why? Same goes for BCM-3277.

*DNS only binds to human RANKL, for that, we use it exclusively in tumors that express RANKL, as in the case of the STG139-M PDX model.*

15) Similar concerns arise in the combinatorial DTX studies. No rationale is given.

*Rationale was provided in the text (Page 11), DNS is a human monoclonal antibody specific for human RANKL, but it does not recognize mouse RANKL. STG139-M expresses both hRANK and hRANKL and for that was treated with denosumab. AB521-X and BCM-3277 do not express hRANKL, thus, they were only treated with RANK-Fc, which binds to both mouse and human RANKL. As expected, RANK-Fc inhibits bone remodeling (driven by mouse RANKL), revealed by the decrease in Trap5b levels, while DNS does not (Fig EV4A).*

16) If you block with RANK-Fc why did the tumor proliferate? This is never discussed.

*RANKL inhibitors (RANK-Fc and DNS) reduced tumor cell proliferation in the STG139-M PDX (which expresses RANK and RANKL), but not in the other two PDXs (Fig EV4C). These results suggest that the reduction of tumor cell proliferation is driven in part by the inhibition of tumor RANKL. This is in line with our previous findings (Gonzalez-Suarez,*

*Nature 2010), showing that RANKL inhibition decreased proliferation in mammary epithelial cells and preneoplasic lesions (where RANKL is expressed) but not in established tumors (where RANKL is not expressed). This is stated in the manuscript (page 10).*

17) Section on pathways the authors state "PDX GSEA demonstrates....200 pathways differentially expresses. TS3 has allot dozens of tabs was not easy to identify, which it is please clarify and identify.

18) FigS5A TS3 "Immunity" pathways? Which comparison again dozen of tabs multiple comparison which one are the authors referring to? S5B RANKL inhibition in three PDX models which three is not clear.

*We have now included a Venn diagram and a classification of the main pathways in Fig 1H of the revised manuscript. A detailed index is included in the first tab of the Table EV3. We have assigned a letter for each tab and included a color coding. The name in the tab indicates whether it includes differentially expressed genes or GSEA. The pathways that are differentially expressed are indicated in bold.*

*In vivo treatments with RANKL inhibitors and gene expression analyses have been done in three independent PDX models, BCM-3277, STG139-M and AB521-X.*

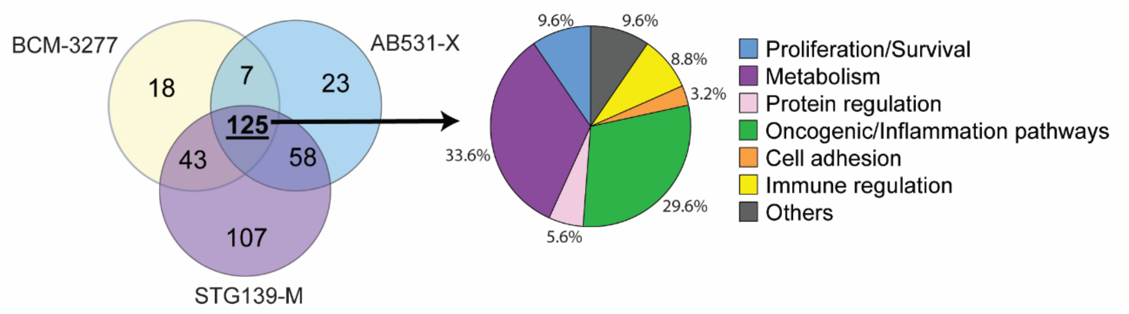


*Fig 1H.*

19) RANKL is regulated by progesterone in mammary gland homeostasis whereas estradiol/opg is the inhibitor of RANK/RANKL signaling (reference 30). Should state that is in bone please clarify inference make it sound like it does so in mammary gland.

*Thanks for pointing this out. OPG is a general physiological inhibitor of RANK signaling that binds to RANKL acting as a dominant negative, so it will inhibit RANKL in all the tissues. The sentence has been rewritten to clarify (page 14).*

20) Fig5a Table S1 it was not clear if RANK was predicting DMFS and BCSS not clear from the data if it was refereeing to ER- or the ER+, please clarify.

*Data have been reorganized to fit the guidelines of a Report. Fig 3A and Fig EV5A in the revised manuscript include results from the heterogeneous cohorts IDB and NPS, according to menopause status. Results for the NPS $ER^-$ subset are in Fig EV5B, while results for the ER-NEGATIVE ONLY collection are in Fig 3B. Figures are labelled accordingly.*

21) Following Fig5B, data from Table S1 was sued to refence "survival of 15/20 years" yet on the data CNIO data was only for 12 years, please clarify, similarly please clarify which chemoresistance data is been represented, not clear which one if been used from the text or the Tables S1.

*In prior Fig 5b (now Fig 3 and Fig EV5), there is not data of the TNBC (CNIO) collection, 5-year survival is represented for samples of the $ER^-$ subset of NPS, while in the ER-NEGATIVE ONLY collection 10-year survival is represented. In Table EV1 survival data for other time-points are shown. Time of follow-up varies between different collections but it is always indicated in the figures or table.*

*The chemotherapy regimens used in each collection are included in the manuscript text, Methods section, page 16, in the TNBC (CNIO) cohort are shown in Fig EV3D (group 1: CMF (cyclophosphamide, methotrexate, 5-fluorouracil), group 2: FAC (5-fluorouracil, doxorubicin, cyclophosphamide) or FEC (5-fluorouracil, epirubicin, cyclophosphamide) and group 3: CMF or FAC or FEC plus taxanes). In the ER-NEGATIVE ONLY cohort the chemotherapy regimen used was CMF and after 2000 anthracyclines plus taxanes.*

22) The rationale to compare PDX data (Fig5C Table S4) with that of the postmenopausal METABRIC data sets is not clear stated at all. How does a PDX in a NSG mice comparison work?

*We believe that the referee may be misinterpreting the data from Table EV4. In Table EV4 we indicate the pathways associated with RANK expression in $ER^+$ and $ER^-$ tumors (Fig 2A). In the PDXs, we identified pathways directly regulated by RANKL/RANK-Fc in each of the tumors. In Table EV4 (tabs H and I) we show the results of the comparison of pathways directly regulated by RANK signaling in the PDXs with those associated with RANK in $ER^-$ and $ER^+$ tumors. The observed overlapping reinforces the hypothesis that RANK directly modulates these pathways in clinical BC and highlights the clinical significance of the PDX models for this study. We only performed the comparisons with the $ER^+$/$ER^-$ cohorts of METABRIC, not with the menopausal cohorts, but the same rationale will apply.*

23) Fig6C data set not significant, why not use denosumab in the AB 521?

*See explanation above, denosumab only binds human RANKL, not mouse.*

Referee #3 (Remarks for Author):

The manuscript is rather interesting as it looks at the role of RANK in breast cancer and leverages the large datasets to do so - but importantly the authors have followed up with wet work to test the hypotheses that they generated. They should be commended for this. Only minor concerns are listed that may aid in the manuscript:

1) Figure 1D - can you split this to the PAM50 subtypes and show that this is not simply a function of basal vs all other subtypes. It would be good to show here that RANK status within a subtype can show altered outcomes.

*Gene expression analyses is not available for these > 2000 samples, and therefore we do not know the PAM50 classification. Microarray data is only available for a subset of NPS collection that were included in METABRIC but the sample size is too small to provide solid conclusions. We agree with the referee that it is important to show the relevance of RANK as a biomarker in the different breast cancer subtypes, particularly as RANK is more frequently expressed in ER-negative BC. The analyses on the $ER^-$ and $ER^+$ cohorts were included in Fig 2A and Fig EV3 of the revised manuscript and Table EV1. We found that RANK associates with poor prognosis in ER-negative breast cancer but not in ER-positive breast cancer. These findings were validated in an independent cohort including only ER-negative tumors, the ER-NEGATIVE ONLY collection.*

*As suggested by the referee, we have also performed the analyses attending to HER2 expression. In the NPS cohort (all patients), RANK+ associated with poor BCSS and DMFS in $HER2^+$ tumors. In the ER-NEGATIVE ONLY cohort, again we found that RANK associates with poor DFS (not BCSS or DMFS) in $ER^-$ $HER2^+$ tumors. As the sample size is small, no solid conclusions can be drawn and it will be essential to confirm these findings in an independent $HER2^+$ cohort, taking into account HER2-targeted therapies, as in our manuscript Sanz-Moreno et al., BCR, 2021.*

*Moreover, we also analyzed RANK prognostic value in BRCA1-mutated tumors and $ER^-$ tumors with basal markers, but again sample size is small to get solid conclusions.*

*Results from these analyses are now included in Table EV1.*

| NOTTINGHAM PRIMARY SERIES (HER2+) | | | | |
|---|---|---|---|---|
| **Parameters** | **Total N** | **N of events** | **Time** | ***p*-value (Log Rank (Mantel-Cox))** |
| **BCSS** | 125 | 38 | 60 months | 0.001 |
| No RANK expression | 118 | 33 | | |
| RANK expression | 7 | 5 | | |
| **BCSS** | 108 | 54 | 180 months | 0.002 |
| No RANK expression | 102 | 49 | | |
| RANK expression | 6 | 5 | | |
| **DMFS** | 124 | 47 | 60 months | 0.009 |
| No RANK expression | 117 | 42 | | |
| RANK expression | 7 | 5 | | |
| **DMFS** | 124 | 60 | 240 months | 0.025 |
| No RANK expression | 117 | 55 | | |
| RANK expression | 7 | 5 | | |
| **DFS** | 125 | 54 | 60 months | 0.05 |
| No RANK expression | 118 | 49 | | |
| RANK expression | 7 | 5 | | |
| **DFS** | 125 | 68 | 240 months | 0.106 |
| No RANK expression | 118 | 63 | | |
| RANK expression | 7 | 5 | | |

| NOTTINGHAM PRIMARY SERIES (Premenopausal HER2+) | | | | |
|---|---|---|---|---|
| **Parameters** | **Total N** | **N of events** | **Time** | ***p*-value (Log Rank (Mantel-Cox))** |
| **BCSS** | 55 | 16 | 60 months | no |
| No RANK expression | 55 | 16 | | |
| RANK expression | | | | |
| **BCSS** | 46 | 28 | 180 months | no |
| No RANK expression | 46 | 28 | | |
| RANK expression | | | | |
| **DMFS** | 56 | 22 | 60 months | no |
| No RANK expression | 56 | 22 | | |
| RANK expression | | | | |
| **DMFS** | 56 | 31 | 240 months | no |
| No RANK expression | 56 | 31 | | |
| RANK expression | | | | |
| **DFS** | 56 | 26 | 60 months | no |
| No RANK expression | 56 | 26 | | |
| RANK expression | | | | |
| **DFS** | 56 | 34 | 240 months | no |
| No RANK expression | 56 | 34 | | |
| RANK expression | | | | |

| NOTTINGHAM PRIMARY SERIES (Postmenopausal HER2+) | | | | |
|---|---|---|---|---|
| **Parameters** | **Total N** | **N of events** | **Time** | ***p*-value (Log Rank (Mantel-Cox))** |
| **BCSS** | 69 | 22 | 60 months | 0.006 |
| No RANK expression | 62 | 17 | | |
| RANK expression | 7 | 5 | | |
| **BCSS** | 61 | 26 | 180 months | 0.004 |
| No RANK expression | 55 | 21 | | |
| RANK expression | 6 | 5 | | |
| **DMFS** | 68 | 25 | 60 months | 0.019 |
| No RANK expression | 61 | 20 | | |
| RANK expression | 7 | 5 | | |
| **DMFS** | 68 | 29 | 240 months | 0.036 |
| No RANK expression | 61 | 24 | | |
| RANK expression | 7 | 5 | | |
| **DFS** | 68 | 28 | 60 months | 0.059 |
| No RANK expression | 61 | 23 | | |
| RANK expression | 7 | 5 | | |
| **DFS** | 471 | 187 | 240 months | 0.284 |
| No RANK expression | 449 | 180 | | |
| RANK expression | 22 | 7 | | |

| ER-NEGATIVE ONLY (HER2+) | | | | |
|---|---|---|---|---|
| **Parameters** | **Total N** | **N of events** | **Time** | ***p*-value (Log Rank (Mantel-Cox))** |
| **BCSS** | 69 | 20 | 60 months | 0.428 |
| No RANK expression | 50 | 13 | | |
| RANK expression | 19 | 7 | | |
| **BCSS** | 69 | 22 | 120 months | 0.135 |
| No RANK expression | 50 | 13 | | |
| RANK expression | 19 | 9 | | |
| **DMFS** | 69 | 24 | 60 months | 0.05 |
| No RANK expression | 50 | 14 | | |
| RANK expression | 19 | 10 | | |
| **DMFS** | 69 | 24 | 120 months | 0.05 |
| No RANK expression | 50 | 14 | | |
| RANK expression | 19 | 10 | | |
| **DFS** | 69 | 27 | 60 months | 0.033 |
| No RANK expression | 50 | 16 | | |
| RANK expression | 19 | 11 | | |
| **DFS** | 69 | 28 | 120 months | 0.044 |
| No RANK expression | 50 | 17 | | |
| RANK expression | 19 | 11 | | |

| ER-NEGATIVE ONLY (Premenopausal HER2+) | | | | |
|---|---|---|---|---|
| **Parameters** | **Total N** | **N of events** | **Time** | ***p*-value (Log Rank (Mantel-Cox))** |
| **BCSS** | 28 | 10 | 60 months | 0.952 |
| No RANK expression | 20 | 7 | | |
| RANK expression | 8 | 3 | | |
| **BCSS** | 28 | 10 | 120 months | 0.952 |
| No RANK expression | 20 | 7 | | |
| RANK expression | 8 | 3 | | |
| **DMFS** | 28 | 12 | 60 months | 0.805 |
| No RANK expression | 20 | 8 | | |
| RANK expression | 8 | 4 | | |
| **DMFS** | 28 | 12 | 120 months | 0.805 |
| No RANK expression | 20 | 8 | | |
| RANK expression | 8 | 4 | | |
| **DFS** | 28 | 13 | 60 months | 0.289 |
| No RANK expression | 20 | 8 | | |
| RANK expression | 8 | 5 | | |
| **DFS** | 28 | 13 | 120 months | 0.289 |
| No RANK expression | 20 | 8 | | |
| RANK expression | 8 | 5 | | |

| ER-NEGATIVE ONLY (Postmenopausal HER2+) | | | | |
|---|---|---|---|---|
| **Parameters** | **Total N** | **N of events** | **Time** | ***p*-value (Log Rank (Mantel-Cox))** |
| **BCSS** | 41 | 10 | 60 months | 0.26 |
| No RANK expression | 30 | 6 | | |
| RANK expression | 11 | 4 | | |
| **BCSS** | 41 | 12 | 120 months | 0.031 |
| No RANK expression | 30 | 6 | | |
| RANK expression | 11 | 6 | | |
| **DMFS** | 41 | 12 | 60 months | 0.018 |
| No RANK expression | 30 | 6 | | |
| RANK expression | 11 | 6 | | |
| **DMFS** | 41 | 12 | 120 months | 0.018 |
| No RANK expression | 30 | 6 | | |
| RANK expression | 11 | 6 | | |
| **DFS** | 41 | 14 | 60 months | 0.064 |
| No RANK expression | 30 | 8 | | |
| RANK expression | 11 | 6 | | |
| **DFS** | 41 | 15 | 120 months | 0.087 |
| No RANK expression | 30 | 9 | | |
| RANK expression | 11 | 6 | | |

| ER-NEGATIVE ONLY (Basal) | | | | |
|---|---|---|---|---|
| **Parameters** | **Total N** | **N of events** | **Time** | ***p*-value (Log Rank (Mantel-Cox))** |
| **BCSS** | 140 | 25 | 60 months | 0.88 |
| No RANK expression | 96 | 17 | | |
| RANK expression | 44 | 8 | | |
| **BCSS** | 140 | 30 | 120 months | 0.461 |
| No RANK expression | 96 | 19 | | |
| RANK expression | 44 | 11 | | |
| **DMFS** | 140 | 27 | 60 months | 0.709 |
| No RANK expression | 96 | 18 | | |
| RANK expression | 44 | 9 | | |
| **DMFS** | 140 | 29 | 120 months | 0.601 |
| No RANK expression | 96 | 19 | | |
| RANK expression | 44 | 10 | | |
| **DFS** | 140 | 32 | 60 months | 0.587 |
| No RANK expression | 96 | 21 | | |
| RANK expression | 44 | 11 | | |
| **DFS** | 140 | 37 | 120 months | 0.74 |
| No RANK expression | 96 | 25 | | |
| RANK expression | 44 | 12 | | |

| ER-NEGATIVE ONLY (Premenopausal basal) | | | | |
|---|---|---|---|---|
| **Parameters** | **Total N** | **N of events** | **Time** | ***p*-value (Log Rank (Mantel-Cox))** |
| **BCSS** | 65 | 9 | 60 months | 0.865 |
| No RANK expression | 41 | 6 | | |
| RANK expression | 24 | 3 | | |
| **BCSS** | 65 | 9 | 120 months | 0.865 |
| No RANK expression | 41 | 6 | | |
| RANK expression | 24 | 3 | | |
| **DMFS** | 65 | 7 | 60 months | 0.666 |
| No RANK expression | 41 | 5 | | |
| RANK expression | 24 | 2 | | |
| **DMFS** | 65 | 7 | 120 months | 0.666 |
| No RANK expression | 41 | 5 | | |
| RANK expression | 24 | 2 | | |
| **DFS** | 65 | 9 | 60 months | 0.364 |
| No RANK expression | 41 | 7 | | |
| RANK expression | 24 | 2 | | |
| **DFS** | 65 | 11 | 120 months | 0.207 |
| No RANK expression | 41 | 9 | | |
| RANK expression | 24 | 2 | | |

| ER-NEGATIVE ONLY (Postmenopausal basal) | | | | |
|---|---|---|---|---|
| **Parameters** | **Total N** | **N of events** | **Time** | ***p*-value (Log Rank (Mantel-Cox))** |
| **BCSS** | 75 | 16 | 60 months | 0.561 |
| No RANK expression | 55 | 11 | | |
| RANK expression | 20 | 5 | | |
| **BCSS** | 75 | 21 | 120 months | 0.169 |
| No RANK expression | 55 | 13 | | |
| RANK expression | 20 | 8 | | |
| **DMFS** | 75 | 20 | 60 months | 0.257 |
| No RANK expression | 55 | 13 | | |
| RANK expression | 20 | 7 | | |
| **DMFS** | 75 | 22 | 120 months | 0.186 |
| No RANK expression | 55 | 14 | | |
| RANK expression | 20 | 8 | | |
| **DFS** | 75 | 23 | 60 months | 0.066 |
| No RANK expression | 55 | 14 | | |
| RANK expression | 20 | 9 | | |
| **DFS** | 75 | 26 | 120 months | 0.062 |
| No RANK expression | 55 | 16 | | |
| RANK expression | 20 | 10 | | |

| ER-NEGATIVE ONLY (BRCA1 WT- all patients) | | | | |
|---|---|---|---|---|
| **Parameters** | **Total N** | **N of events** | **Time** | ***p*-value (Log Rank (Mantel-Cox))** |
| **BCSS** | 239 | 50 | 60 months | 0.714 |
| No RANK expression | 163 | 33 | | |
| RANK expression | 76 | 17 | | |
| **BCSS** | 239 | 59 | 120 months | 0.199 |
| No RANK expression | 163 | 36 | | |
| RANK expression | 76 | 23 | | |
| **DMFS** | 239 | 58 | 60 months | 0.376 |
| No RANK expression | 163 | 37 | | |
| RANK expression | 76 | 21 | | |
| **DMFS** | 239 | 64 | 120 months | 0.24 |
| No RANK expression | 163 | 40 | | |
| RANK expression | 76 | 24 | | |
| **DFS** | 239 | 66 | 60 months | 0.29 |
| No RANK expression | 163 | 42 | | |
| RANK expression | 76 | 24 | | |
| **DFS** | 239 | 73 | 120 months | 0.321 |
| No RANK expression | 163 | 47 | | |
| RANK expression | 76 | 26 | | |

| ER-NEGATIVE ONLY (BRCA1-mutated all patients) | | | | |
|---|---|---|---|---|
| **Parameters** | **Total N** | **N of events** | **Time** | ***p*-value (Log Rank (Mantel-Cox))** |
| **BCSS** | 24 | 2 | 60 months | 0.106 |
| No RANK expression | 13 | 0 | | |
| RANK expression | 11 | 2 | | |
| **BCSS** | 24 | 2 | 120 months | 0.106 |
| No RANK expression | 13 | 0 | | |
| RANK expression | 11 | 2 | | |
| **DMFS** | 24 | 2 | 60 months | 0.115 |
| No RANK expression | 13 | 0 | | |
| RANK expression | 11 | 2 | | |
| **DMFS** | 24 | 2 | 120 months | 0.115 |
| No RANK expression | 13 | 0 | | |
| RANK expression | 11 | 2 | | |
| **DFS** | 24 | 5 | 60 months | 0.006 |
| No RANK expression | 13 | 0 | | |
| RANK expression | 11 | 5 | | |
| **DFS** | 24 | 5 | 120 months | 0.006 |
| No RANK expression | 13 | 0 | | |
| RANK expression | 11 | 5 | | |

2) Figure 2 - I would suggest bringing some of the supplemental data for a non-responsive line as a control into the main figure (2D).

*The manuscript has been reorganized to fit the format of a Report, as requested by the editors. Attending to the suggestion of the referee, the WB to test NFKB activation upon RANKL stimulation in all the models are shown together in Fig EV2C. As only three main figures are allowed they could not be included in the main figure.*

3) RNAseq data - I was unable to review the data deposited to GeoDatasets. Please make a reviewer token available so that the data can be reviewed PRIOR to publication. An embargo until publication is fine, but please provide a reviewer link and token.

*We apologize for the inconvenience, the token was provided in the cover letter but not in the manuscript. RNAseq results have been deposited in GEO: GSE185513 study (https://www.ncbi.nlm.nih.gov/geo/query/acc.cgi?acc=GSE185513).*
*Token for reviewers is izcjgemgxrwrbkj.*

4) GSEA - again, I'd suggest bringing this into the main portion of the manuscript. Cut some of Figure 2C and bring some of the data from table S3 in as a GSEA based figure showing the random walk.

*Thanks for the suggestion. Fig 1H now includes results from GSEA analyses in the PDXs.*

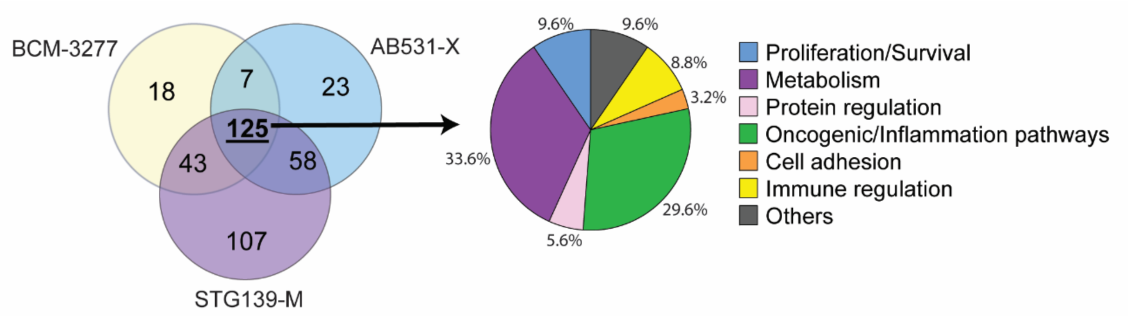


*Fig 1H.*

5) FIgure 3B does not indicate in the legend or on the figure which is RANK +ve or -ve

*Thanks for noticing this; it has now been corrected (Fig EV3A).*

6) Figure 5 - again, split out the subtypes and do the appropriate statistical tests.

*Data has been reorganized to fit the guidelines of a Report. Menopausal findings are now shown in Fig 3 and Fig EV5 of the revised manuscript. Fig 3A and Fig EV5A show data from the heterogeneous IDB and NPS cohorts, while Fig 3B and EV5B include data of the ER$^-$ cohorts, the ER-NEGATIVE ONLY collection and NPS ER$^-$ subset. In all cases RANK associates with poor survival in postmenopausal patients. Figures are labelled accordingly. All results are included in Table EV1.*

*As the referee suggested in the revised manuscript, we have included survival data according to HER2 and basal marker expression and we have observed that RANK associates with poor survival in postmenopausal HER2$^+$ patients, but again sample size is too small for the findings to be conclusive.*

16th Jan 2023

Dear Dr. Gonzalez-Suarez,

Thank you for the submission of your revised manuscript to EMBO Molecular Medicine, and please accept my apologies for the delay in getting back to you following this very busy time of the year. We have now received the enclosed reports from the three initial referees. As you will see below, while referees #2 and #3 are satisfied with the revision, referee #1 still raises a few concerns that should be addressed in a last round of revisions. In particular, the point raised by the referee regarding differences between post-menopausal vs. pre-menopausal conditions should be either addressed experimentally, convincingly discussed, or as suggested by the reviewer, panel 3E should be removed. Other concerns might be addressed in writing.

Moreover, please address the following editorial points:

1/ Main manuscript text:
- Please address the queries (figure legends) from our data editors in the related Data Edited file in track changes mode. Please keep in track changes mode any new modification in the manuscript text.
- We can accommodate a maximum of 5 keywords. Am I correct to assume your keywords are: 1/ breast cancer patients-derived xenografts; 2/ER- Breast cancer; 3/menopause; 4/pharmacological RANKL inhibitors; 5/RANK-RANKL?
- Material and methods:
o Human samples: Please include a statement confirming that informed consent was obtained from all subjects and that the experiments conformed to the principles set out in the WMA Declaration of Helsinki and the Department of Health and Human Services Belmont Report.
o Animal experiments: please indicate the origin of the mice.
o Please provide the antibody dilutions.
o Statistics: please include a statement about blinding, randomization and exclusion criteria.
- Data Availability Section: Thank you for depositing your datasets in a public repository. Please note that the data must be publicly available before acceptance of the manuscript.
- Please merge the Acknowledgements and Funding sections, and make sure that the information provided in the manuscript matches the information provided in the submission system.
- Please update "Conflict of interest" by "Disclosure statement and competing interests". (We updated our journal's competing interests policy in January 2022 and request authors to consider both actual and perceived competing interests. Please review the policy https://www.embopress.org/competing-interests and update your competing interests if necessary.)

2/ Figures:
You currently have 4 EV tables: please add the legends to the tables that should be renamed Datasets EV1-4 (please update the callouts in the manuscript text accordingly).

3/ At EMBO Press we encourage authors to provide source data for the main figures. Please see:
https://www.embopress.org/page/journal/17574684/authorguide#sourcedata

4/ In the checklist, please indicate if relevant guidelines (i.e. ARRIVE) have been followed or provided.
You also filled out the section about human clinical and genomic datasets deposited in public repositories, please confirm that this is correct.

5/ Thank you for providing The Paper Explained. I added minor modifications, please amend as you see fit:
Problem
The search for new prognostic factors and therapeutic targets has become an essential task for the individualization of breast cancer therapy. RANK signaling pathway has emerged a new target for breast cancer based on compelling preclinical evidence. RANKL inhibition prevents or attenuates mammary tumor initiation and induces tumor cell differentiation and an anti-tumorigenic immune response in established tumors. However, in clinical trials the therapeutic benefit of the RANKL inhibitor denosumab in breast cancer, beyond its bone related effects, is unclear. Given the heterogeneity of breast cancer, a better understanding of RANK biology is needed to identify the patients who may benefit from denosumab.
Results
Here, we report the expression patterns of RANK and RANKL proteins in more than 2000 breast tumor samples from independent collections, together with functional studies in breast cancer patient-derived xenografts (PDXs). Our results demonstrate that RANK in tumors cells constitutes a new independent biomarker of poor prognosis in patients with ER- tumors and in postmenopausal women. Accordingly, RANKL inhibition improves response to chemotherapy in ER- BC PDXs, reducing recurrence, and show a greater therapeutic effect in ER- BC tumors growing in postmenopausal conditions. The distinct biology of RANK signaling according to ER expression and menopause enlighten these paradoxical results: RANK activation increases in tumors after menopause and regulates tumor cell metabolism in ER- disease.
Impact

Our findings identify RANK as a new biomarker of poor prognosis in postmenopausal women with ER- breast tumors. These results will help to identify breast cancer patients who can benefit from denosumab in a personalized therapeutic strategy.

6/ As part of the EMBO Publications transparent editorial process initiative (see our Editorial at http://embomolmed.embopress.org/content/2/9/329), EMBO Molecular Medicine will publish online a Review Process File (RPF) to accompany accepted manuscripts.
This file will be published in conjunction with your paper and will include the anonymous referee reports, your point-by-point response and all pertinent correspondence relating to the manuscript. Let us know whether you agree with the publication of the RPF and as here, if you want to remove or not any figures from it prior to publication.
Please note that the Authors checklist will be published at the end of the RPF.

I look forward to receiving your revised manuscript.

Yours sincerely,

Lise Roth

Lise Roth, PhD
Senior Editor
EMBO Molecular Medicine

***** Reviewer's comments *****

Referee #1 (Remarks for Author):

In this revised manuscript, the authors changed format to a Report, so as to bypass the need for mechanistic insight required for a full paper.

The authors show that RANKL inhibitions cooperate with chemotherapy (docetaxel) to suppress ER-negative/RANK+ PDX growth and that RANK tumor expression associates with poor survival in postmenopausal patients.

They addressed some concerns but not others:

First, and most importantly, the authors show that RANKL inhibition attenuates tumor growth of a RANK+ ER- BC PDX in ovariectomized mice (postmenopausal conditions - Fig. 3E in revised manuscript) - and that this inhibition is stronger than that seen in a similar experiment in pre-menopausal mice ( Fig. 2F ). However, these experiments were not done side-by-side and may therefore reflect experiment-to-experiment (batch-to-batch) variations rather than a qualitative difference. Notably, in AB521-X cells - treatment accelerated growth in Fig 2F. Is this reproducible in independent biological replicas? How do the authors explain this response in these cells as opposed to the other lines? Or are the two groups (red - blue) switched?

The Reviewer noted in the initial review:
"Fig. 6 shows the results in ovariectomized NSG mice. To demonstrate specificity, the authors should show side-by-side the effect of RANKL inhibition alone in normal mice as shown in Fig. 4a." The authors response "We decided to maintain the results from premenopausal and postmenopausal conditions in separate figures to facilitate the comprehension of the manuscript." - is beside the point as the request was to show the post-menopausal experiment side-by-side with a (new/additional) pre-menopausal experiment. This is critical because while the difference for AB521-X cells is dramatic - but is it real (see above)? - the difference for the other line - BCM-3277 - is moderate (P=0.003 vs P=0.011) and may be due to differences in other

experimental conditions/variables (e.g. drug activity, number of cells injected etc).

These are tough experiments and I appreciate the difficulty in repeating them as this stage. However, can the authors justify why they are convinced the differences between post-menopausal vs pre-menopausal conditions are real without performing the experiments side-by-side? If not, they may remove Fig. 3E - and highlight the other results in this manuscript.

- The authors response to the question " Does RANK-shRNA have similar effect as RANKL-inhibitors?" - that "RANKL inhibitors will have a systemic effect", may be correct - but still the question whether denosumab or denosumab - DTX inhibits growth of cells in vitro - and whether such inhibition is seen with shRNA or RNAi (or CRISPR/CAS) are very informative. If RANKL-inhibitors (plus/minus DTX) do not have any effects in vitro - that would strengthen the idea that they act on the microenvironment. If, on the other hand - these drugs do suppress growth in vitro - the question is whether shRNA/RNAi-depletion (or crispr/cas9 KO) would have a similar effect - because otherwise, these inhibitors may have off target effects.

However - this analysis may be performed as part of future followup. The issue above regarding post-menopausal sensitivity is more critical to this manuscript.

Other issues

- Fig 2G bottom right - what cells are these? How is it different than bottom left?

- Abstract
In the revised Abstract - the definition of denosumab has been deleted from the first sentence (which appeared in the original Abstract). It should be defined again as "Despite strong preclinical data, the therapeutic benefit of the RANKL inhibitor, denosumab, in breast cancer patients is unclear, ...".

Second sentece should start with "Aiming to select patients who may benefit from
denosumab, we hereby analyzed ...."

Second/subsequent sentences - when referring to "RANK and RANKL expression" - the author should specify what they mean - e.g. "RANK and RANKL expression by immunostaining"; "RANK and RANKL protein expression"(as per last sentence)

The Abstract starts with the therapeutic benefit of denosumab - but this drug is not mentioned in the rest of the Abstract. It should be used in the middle of the Abstract and surely in the end - or more specifically state "RANKL inhibitors RANK-Fc or denosumab....."

Referee #2 (Comments on Novelty/Model System for Author):

EVERY CONCERNS WAS ADDRESSED.

Referee #2 (Remarks for Author):

The authors have worked extremely hard to revise the manuscript, and have addressed the multiple concerns that were raised in the earlier version. Moreover, the revised version reads much clearer and will be received well by the general readership. Well done!

Referee #3 (Comments on Novelty/Model System for Author):

Sufficient novelty and medical impact for publication.

Referee #3 (Remarks for Author):

Thank you for addressing all the concerns.

***** Reviewer's comments *****

Referee #1 (Remarks for Author):

In this revised manuscript, the authors changed format to a Report, so as to bypass the need for mechanistic insight required for a full paper.

The authors show that RANKL inhibitions cooperate with chemotherapy (docetaxel) to suppress ER-negative/RANK+ PDX growth and that RANK tumor expression associates with poor survival in postmenopausal patients.

They addressed some concerns but not others:

First, and most importantly, the authors show that RANKL inhibition attenuates tumor growth of a RANK+ ER- BC PDX in ovariectomized mice (postmenopausal conditions - Fig. 3E in revised manuscript) - and that this inhibition is stronger than that seen in a similar experiment in pre-menopausal mice ( Fig. 2F ). However, these experiments were not done side-by-side and may therefore reflect experiment-to-experiment (batch-to-batch) variations rather than a qualitative difference. Notably, in AB521-X cells - treatment accelerated growth in Fig 2F. Is this reproducible in independent biological replicas? How do the authors explain this response in these cells as opposed to the other lines? Or are the two groups (red - blue) switched?

The Reviewer noted in the initial review:
"Fig. 6 shows the results in ovariectomized NSG mice. To demonstrate specificity, the authors should show side-by-side the effect of RANKL inhibition alone in normal mice as shown in Fig. 4a." The authors response "We decided to maintain the results from premenopausal and postmenopausal conditions in separate figures to facilitate the comprehension of the manuscript." - is beside the point as the request was to show the post-menopausal experiment side-by-side with a (new/additional) pre-menopausal experiment. This is critical because while the difference for AB521-X cells is dramatic - but is it real (see above)? - the difference for the other line - BCM-3277 - is moderate (P=0.003 vs P=0.011) and may be due to differences in other experimental conditions/variables (e.g. drug activity, number of cells injected etc).

These are tough experiments and I appreciate the difficulty in repeating them as this stage. However, can the authors justify why they are convinced the differences between post-menopausal vs pre-menopausal conditions are real without performing the experiments side-by-side? If not, they may remove Fig. 3E - and highlight the other results in this manuscript.

Per request of the referee, we have eliminated panel 3E, as well as EV5 C and EV5 D. In future work, we aim to address the functional mechanisms underlying the differences between pre and postmenopausal conditions.
We confirmed that in Figure 2F the two groups (red-blue) are not switched but the difference is so small that it is most probably due to tumor-to-tumor variability.

- The authors response to the question " Does RANK-shRNA have similar effect as RANKL-inhibitors?" - that "RANKL inhibitors will have a systemic effect", may be correct - but still the question whether denosumab or denosumab - DTX inhibits growth of cells in vitro - and whether such inhibition is seen with shRNA or RNAi (or CRISPR/CAS) are very informative. If RANKL-inhibitors (plus/minus DTX) do not have any effects in vitro - that would strengthen the idea that they act on the microenvironment. If, on the other hand - these drugs do suppress

growth in vitro - the question is whether shRNA/RNAi-depletion (or crispr/cas9 KO) would have a similar effect - because otherwise, these inhibitors may have off target effects.
However - this analysis may be performed as part of future followup. The issue above regarding post-menopausal sensitivity is more critical to this manuscript.

We agree with the referee that it will be informative to compare inhibition of the receptor and the ligand. However, as explained in the previous point by point, denosumab is a monoclonal antibody against human RANKL. Therefore would only act in vitro in models where RANKL is expressed, such as STG139-M. The other models and most breast cancer cell lines express the receptor but not the ligand. It cannot be discarded that the culture media/serum may act as a source of RANKL (most probably not human RANKL).

Other issues

- Fig 2G bottom right - what cells are these? How is it different than bottom left?

It is the same model, STG139, and the same experiment. Bottom left shows tumor growth/regression during DTX/RANKL-inhibitor treatment. Bottom right shows the tumor relapse in these same mice after interruption of the combined treatment. We have included a sentence in the methods explaining that treatment was interrupted when tumors regress below 3 mm of diameter. In the docetaxel-only arm, despite treatment could not be interrupted tumors continued growing.

- Abstract
In the revised Abstract - the definition of denosumab has been deleted from the first sentence (which appeared in the original Abstract). It should be defined again as "Despite strong preclinical data, the therapeutic benefit of the RANKL inhibitor, denosumab, in breast cancer patients is unclear, ...".

Second sentece should start with "Aiming to select patients who may benefit from denosumab, we hereby analyzed ...."

Second/subsequent sentences - when referring to "RANK and RANKL expression" - the author should specify what they mean - e.g. "RANK and RANKL expression by immunostaining"; "RANK and RANKL protein expression"(as per last sentence)

The Abstract starts with the therapeutic benefit of denosumab - but this drug is not mentioned in the rest of the Abstract. It should be used in the middle of the Abstract and surely in the end - or more specifically state "RANKL inhibitors RANK-Fc or denosumab....."

We have followed the recommendations of the referee.

Referee #2 (Comments on Novelty/Model System for Author):

EVERY CONCERNS WAS ADDRESSED.

Referee #2 (Remarks for Author):

The authors have worked extremely hard to revise the manuscript, and have addressed the multiple concerns that were raised in the earlier version. Moreover, the revised version reads much clearer and will be received well by the general readership. Well done!

Referee #3 (Comments on Novelty/Model System for Author):

Sufficient novelty and medical impact for publication.

Referee #3 (Remarks for Author):

Thank you for addressing all the concerns.

8th Feb 2023

Dear Dr. Gonzalez-Suarez,

Thank you for the submission of your revised manuscript to EMBO Molecular Medicine. We have now received the report from the referee who assessed the final revisions. As you will see, this referee is now supportive of publication, and I am therefore pleased to inform you that your manuscript is accepted for publication and is now being sent to our publisher to be included in the next available issue of EMBO Molecular Medicine.

Please read below for additional IMPORTANT information regarding your article, its publication and the production process.

Congratulations on your interesting work,

With kind regards,

Lise Roth

Lise Roth, Ph.D
Senior Editor
EMBO Molecular Medicine

Follow us on Twitter @EmboMolMed
Sign up for eTOCs at embopress.org/alertsfeeds

***** Reviewer's comments *****

Referee #2 (Comments on Novelty/Model System for Author):

The authors have revised the manuscript in response to critic adequately

Referee #2 (Remarks for Author):

The author have address all the concerns raised.

*** *** *** IMPORTANT INFORMATION *** *** ***

SPEED OF PUBLICATION
The journal aims for rapid publication of papers, using using the advance online publication "Early View" to expedite the process: A properly copy-edited and formatted version will be published as "Early View" after the proofs have been corrected. Please help the Editors and publisher avoid delays by providing e-mail address(es), telephone and fax numbers at which author(s) can be contacted.

Should you be planning a Press Release on your article, please get in contact with embomolmed@wiley.com as early as possible, in order to coordinate publication and release dates.

LICENSE AND PAYMENT:

All articles published in EMBO Molecular Medicine are fully open access: immediately and freely available to read, download and share.

EMBO Molecular Medicine charges an article processing charge (APC) to cover the publication costs. You, as the corresponding author for this manuscript, should have already received a quote with the article processing fee separately. Please let us know in case this quote has not been received.

Once your article is at Wiley for editorial production you will receive an email from Wiley's Author Services system, which will ask you to log in and will present you with the publication license form for completion. Within the same system the publication fee can be paid by credit card, an invoice, pro forma invoice or purchase order can be requested.

Payment of the publication charge and the signed Open Access Agreement form must be received before the article can be published online.

PROOFS

You will receive the proofs by e-mail approximately 2 weeks after all relevant files have been sent o our Production Office. Please return them within 48 hours and if there should be any problems, please contact the production office at embopressproduction@wiley.com.

Please inform us if there is likely to be any difficulty in reaching you at the above address at that time. Failure to meet our deadlines may result in a delay of publication.

All further communications concerning your paper proofs should quote reference number EMM-2022-16715-V3 and be directed to the production office at embopressproduction@wiley.com.

## EMBO Press Author Checklist

| | |
|---|---|
| Corresponding Author Name: Eva Gonzalez-Suarez | |
| Journal Submitted to: EMBO Molecular Medicine | |
| Manuscript Number: EMM-2022-16715 | |

**USEFUL LINKS FOR COMPLETING THIS FORM**
The EMBO Journal - Author Guidelines
EMBO Reports - Author Guidelines
Molecular Systems Biology - Author Guidelines
EMBO Molecular Medicine - Author Guidelines

**Reporting Checklist for Life Science Articles (updated January 2022)**
This checklist is adapted from Materials Design Analysis Reporting (MDAR) Checklist for Authors. MDAR establishes a minimum set of requirements in transparent reporting in the life sciences (see Statement of Task: 10.31222/osf.io/9sm4x). Please follow the journal's guidelines in preparing your manuscript.
**Please note that a copy of this checklist will be published alongside your article.**

**Abridged guidelines for figures**
**1. Data**
The data shown in figures should satisfy the following conditions:
- the data were obtained and processed according to the field's best practice and are presented to reflect the results of the experiments in an accurate and unbiased manner.
- ideally, figure panels should include only measurements that are directly comparable to each other and obtained with the same assay.
- plots include clearly labeled error bars for independent experiments and sample sizes. Unless justified, error bars should not be shown for technical replicates.
- if n<5, the individual data points from each experiment should be plotted. Any statistical test employed should be justified.
- Source Data should be included to report the data underlying figures according to the guidelines set out in the authorship guidelines on Data Presentation.

**2. Captions**
Each figure caption should contain the following information, for each panel where they are relevant:
- a specification of the experimental system investigated (eg cell line, species name).
- the assay(s) and method(s) used to carry out the reported observations and measurements.
- an explicit mention of the biological and chemical entity(ies) that are being measured.
- an explicit mention of the biological and chemical entity(ies) that are altered/varied/perturbed in a controlled manner.
- the exact sample size (n) for each experimental group/condition, given as a number, not a range;
- a description of the sample collection allowing the reader to understand whether the samples represent technical or biological replicates (including how many animals, litters, cultures, etc.).
- a statement of how many times the experiment shown was independently replicated in the laboratory.
- definitions of statistical methods and measures:
  - common tests, such as t-test (please specify whether paired vs. unpaired), simple $\chi^2$ tests, Wilcoxon and Mann-Whitney tests, can be unambiguously identified by name only, but more complex techniques should be described in the methods section;
  - are tests one-sided or two-sided?
  - are there adjustments for multiple comparisons?
  - exact statistical test results, e.g., P values = x but not P values < x;
  - definition of 'center values' as median or average;
  - definition of error bars as s.d. or s.e.m.

**Please complete ALL of the questions below.**
**Select "Not Applicable" only when the requested information is not relevant for your study.**

**Materials**

| Newly Created Materials | Information included in the manuscript? | In which section is the information available? (Reagents and Tools Table, Materials and Methods, Figures, Data Availability Section) |
|---|---|---|
| New materials and reagents need to be available; do any restrictions apply? | Not Applicable | |

| Antibodies | Information included in the manuscript? | In which section is the information available? (Reagents and Tools Table, Materials and Methods, Figures, Data Availability Section) |
|---|---|---|
| For **antibodies** provide the following information:<br>- Commercial antibodies: RRID (if possible) or supplier name, catalogue number and or/clone number<br>- Non-commercial: RRID or citation | Yes | Methods section |

| DNA and RNA sequences | Information included in the manuscript? | In which section is the information available? (Reagents and Tools Table, Materials and Methods, Figures, Data Availability Section) |
|---|---|---|
| **Short novel DNA or RNA including primers, probes**: provide the sequences. | Yes | Methods section |

| Cell materials | Information included in the manuscript? | In which section is the information available? (Reagents and Tools Table, Materials and Methods, Figures, Data Availability Section) |
|---|---|---|
| **Cell lines:** Provide species information, strain. Provide accession number in repository **OR** supplier name, catalog number, clone number, and/**OR** RRID. | Not Applicable | |
| **Primary cultures:** Provide species, strain, sex of origin, genetic modification status. | Not Applicable | |
| Report if the cell lines were recently **authenticated** (e.g., by STR profiling) and tested for mycoplasma contamination. | Not Applicable | |

| Experimental animals | Information included in the manuscript? | In which section is the information available? (Reagents and Tools Table, Materials and Methods, Figures, Data Availability Section) |
|---|---|---|
| **Laboratory animals or Model organisms:** Provide species, strain, sex, age, genetic modification status. Provide accession number in repository **OR** supplier name, catalog number, clone number, **OR** RRID. | Yes | Methods section |
| **Animal observed in or captured from the field:** Provide species, sex, and age where possible. | Not Applicable | |
| Please detail **housing and husbandry conditions**. | Yes | Methods section |

| Plants and microbes | Information included in the manuscript? | In which section is the information available? (Reagents and Tools Table, Materials and Methods, Figures, Data Availability Section) |
|---|---|---|
| **Plants:** provide species and strain, ecotype and cultivar where relevant, unique accession number if available, and source (including location for collected wild specimens). | Not Applicable | |
| **Microbes:** provide species and strain, unique accession number if available, and source. | Not Applicable | |

| Human research participants | Information included in the manuscript? | In which section is the information available? (Reagents and Tools Table, Materials and Methods, Figures, Data Availability Section) |
|---|---|---|
| If collected and within the bounds of privacy constraints report on age, sex and gender or ethnicity for all study participants. | Yes | Table1 and Methods Section |

| Core facilities | Information included in the manuscript? | In which section is the information available? (Reagents and Tools Table, Materials and Methods, Figures, Data Availability Section) |
|---|---|---|
| If your work benefited from core facilities, was their service mentioned in the acknowledgments section? | Yes | Acknowledgements |

**Design**

| Study protocol | Information included in the manuscript? | In which section is the information available? (Reagents and Tools Table, Materials and Methods, Figures, Data Availability Section) |
|---|---|---|
| If study protocol has been **pre-registered, provide DOI in the manuscript**. For clinical trials, provide the trial registration number **OR** cite DOI. | Not Applicable | |
| Report the **clinical trial registration number** (at ClinicalTrials.gov or equivalent), where applicable. | Not Applicable | |

| Laboratory protocol | Information included in the manuscript? | In which section is the information available? (Reagents and Tools Table, Materials and Methods, Figures, Data Availability Section) |
|---|---|---|
| Provide DOI OR other citation details if **external detailed step-by-step protocols** are available. | Yes | Methods section |

| Experimental study design and statistics | Information included in the manuscript? | In which section is the information available? (Reagents and Tools Table, Materials and Methods, Figures, Data Availability Section) |
|---|---|---|
| Include a statement about **sample size** estimate even if no statistical methods were used. | Yes | Methods section |
| Were any steps taken to minimize the effects of subjective bias when allocating animals/samples to treatment (e.g. **randomization procedure**)? If yes, have they been described? | Yes | Methods section: Statistics |
| Include a statement about **blinding** even if no blinding was done. | Yes | Methods section: Statistics |
| Describe **inclusion/exclusion criteria** if samples or animals were excluded from the analysis. Were the criteria pre-established?<br><br>If sample or data points were omitted from analysis, report if this was due to attrition or intentional exclusion and provide justification. | Yes | Methods section: Statistics. The criteria of exclusion were pre-established |
| For every figure, are **statistical tests** justified as appropriate? Do the data meet the assumptions of the tests (e.g., normal distribution)? Describe any methods used to assess it. Is there an estimate of variation within each group of data? Is the variance similar between the groups that are being statistically compared? | Yes | Methods section and Figure legends |

| Sample definition and in-laboratory replication | Information included in the manuscript? | In which section is the information available? (Reagents and Tools Table, Materials and Methods, Figures, Data Availability Section) |
|---|---|---|
| In the figure legends: state number of times the experiment was **replicated** in laboratory. | Yes | Figure legends, Figures and Methods section |
| In the figure legends: define whether data describe **technical or biological replicates**. | Yes | Figure legends and Methods section |

## Ethics

| Ethics | Information included in the manuscript? | In which section is the information available? (Reagents and Tools Table, Materials and Methods, Figures, Data Availability Section) |
|---|---|---|
| Studies involving **human participants**: State details of **authority granting ethics approval** (IRB or equivalent committee(s), provide reference number for approval. | Yes | Study approval section |
| Studies involving **human participants**: Include a statement confirming that **informed consent** was obtained from all subjects and that the experiments conformed to the principles set out in the WMA Declaration of Helsinki and the Department of Health and Human Services Belmont Report. | Yes | Study approval section |
| Studies involving **human participants:** For publication of **patient photos**, include a statement confirming that consent to publish was obtained. | Not Applicable | |
| Studies involving experimental **animals**: State details of **authority granting ethics approval** (IRB or equivalent committee(s), provide reference number for approval. Include a statement of compliance with ethical regulations. | Yes | Study approval section |
| Studies involving **specimen and field samples:** State if relevant **permits** obtained, provide details of authority approving study; if none were required, explain why. | Not Applicable | |

| Dual Use Research of Concern (DURC) | Information included in the manuscript? | In which section is the information available? (Reagents and Tools Table, Materials and Methods, Figures, Data Availability Section) |
|---|---|---|
| Could your study fall under dual use research restrictions? Please check biosecurity documents and list of **select agents and toxins** (CDC): https://www.selectagents.gov/sat/list.htm | Not Applicable | |
| If you used a select agent, is the security level of the lab appropriate and reported in the manuscript? | Not Applicable | |
| If a study is subject to dual use research of concern regulations, is the name of the **authority granting approval and reference number** for the regulatory approval provided in the manuscript? | Not Applicable | |

## Reporting

The MDAR framework recommends adoption of discipline-specific guidelines, established and endorsed through community initiatives. Journals have their own policy about requiring specific guidelines and recommendations to complement MDAR.

| Adherence to community standards | Information included in the manuscript? | In which section is the information available? (Reagents and Tools Table, Materials and Methods, Figures, Data Availability Section) |
|---|---|---|
| State if relevant guidelines or checklists (e.g., **ICMJE, MIBBI, ARRIVE, PRISMA**) have been followed or provided. | Not Applicable | We have not followed any specific guideline |
| For **tumor marker prognostic studies,** we recommend that you follow the **REMARK** reporting guidelines (see link list at top right). See author guidelines, under 'Reporting Guidelines'. Please confirm you have followed these guidelines. | Yes | Results. Significant results are confirmed in independent datasets. |
| For **phase II and III randomized controlled trials**, please refer to the **CONSORT** flow diagram (see link list at top right) and submit the CONSORT checklist (see link list at top right) with your submission. See author guidelines, under 'Reporting Guidelines'. Please confirm you have submitted this list. | Not Applicable | |

## Data Availability

| Data availability | Information included in the manuscript? | In which section is the information available? (Reagents and Tools Table, Materials and Methods, Figures, Data Availability Section) |
|---|---|---|
| Have **primary datasets** been deposited according to the journal's guidelines (see 'Data Deposition' section) and the respective accession numbers provided in the Data Availability Section? | Yes | Methods section (RNA sequencing) |
| Were **human clinical and genomic datasets** deposited in a public access-controlled repository in accordance to ethical obligations to the patients and to the applicable consent agreement? | Yes | Methods section and Study approval |
| Are **computational models** that are central and integral to a study available without restrictions in a machine-readable form? Were the relevant accession numbers or links provided? | Not Applicable | |
| If publicly available data were reused, provide the respective **data citations in the reference list**. | Yes | Methods and References sections |
